# Optimal duration of DAPT after second-generation drug-eluting stent in acute coronary syndrome

**DOI:** 10.1371/journal.pone.0207386

**Published:** 2018-11-26

**Authors:** Ji-Yong Jang, Dong-Ho Shin, Jung-Sun Kim, Sung-Jin Hong, Chul-Min Ahn, Byeong-Keuk Kim, Young-Guk Ko, Donghoon Choi, Myeong-Ki Hong, Kyung Woo Park, Hyeon-Cheol Gwon, Hyo-Soo Kim, Yangsoo Jang

**Affiliations:** 1 Division of Cardiology, Chungju Medical Center, Chungju, South Korea; 2 Division of Cardiology, Severance Cardiovascular Hospital, Yonsei University Health System, Seoul, South Korea; 3 Cardiovascular Research Institute, Yonsei University College of Medicine, Seoul, South Korea; 4 Division of Cardiology, Samsung Medical Center, Sungkyunkwan University College of Medicine, Seoul, South Korea; 5 Division of Cardiology, Seoul National University Hospital School of Medicine, Seoul National University College of Medicine, Seoul, South Korea; Universita degli Studi Magna Graecia di Catanzaro, ITALY

## Abstract

**Background:**

We evaluated optimal duration of dual antiplatelet therapy (DAPT) after second-generation drug-eluting stent (DES) implantation in acute coronary syndrome (ACS).

**Material and methods:**

From pooled analysis of three randomized clinical trials (EXCELLENT, IVUS-XPL, RESET), a total of 2,216 patient with ACS undergoing second-generation DES implantation were selected. Each study randomized patients to a short-duration DAPT arm (n = 1119; ≤6 months) or a standard-duration DAPT arm (n = 1097; ≥12 months). Two-thirds of patients were male, and their mean age was 63 years. Mean DAPT durations were 164 ±76 and 359 ±68 days, respectively. The primary endpoint was composite of cardiac death, myocardial infarction, stent thrombosis, stroke or major bleeding during the first 12 months after implantation, analyzed according to the intention-to-treat population.

**Results:**

Demographic characteristics were balanced between groups. Mean DAPT duration was 164 and 359 days, respectively. Primary endpoint occurred in 22 patients with short-DAPT and 21 patients with standard-DAPT (2.0% versus 1.9%; hazard ratio [HR] 1.03; 95% confidence interval [CI] 0.56–1.86; p = 0.94). Landmark analysis after six-months, no significant difference in primary endpoint between short and standard duration DAPT (1.0% versus 0.8%; HR 1.22; 95% CI 0.51–2.95; p = 0.66).

**Conclusions:**

Short-duration DAPT (≤6 months) demonstrated a similar incidence of net adverse cardiovascular and clinical events at 12 months after second-generation DES in ACS compared with standard duration DAPT (≥12 months).

**Clinical trial registration:**

EXCELLENT (ClinicalTrials.gov, NCT00698607), RESET (ClinicalTrials.gov, NCT01145079), IVUS-XPL (ClinicalTrials.gov, NCT01308281)

## Introduction

Dual antiplatelet therapy (DAPT) after drug-eluting stent (DES) implantation has become standard therapy. [1] The optimal duration of DAPT recommended for first-generation DESs was at least 12 months, but recent studies demonstrated short-duration (≤6 months) DAPT show non-inferior efficacy and safety compared to longer-duration (≥12 months) DAPT in patients with second-generation DESs. [2–6] Based on these studies, 6 months of DAPT is now considered a reasonable approach in patients with stable angina receiving second-generation DESs.

By contrast, recommendations for the optimal duration of DAPT have not changed for patients with acute coronary syndrome (ACS), even with the use of second-generation DESs, as data to support recommendation modifications are lacking. ACS is well known to increase the risk of recurrent ischemic events, and a standard duration of DAPT (≥12 months) may help prevent these events. A recent meta-analysis suggested that the net clinical benefit of different DAPT durations might vary according to the clinical presentation. [7] However, second-generation DESs were reported to attenuate the benefit of a standard duration of DAPT compared to short-duration (≤6 months) DAPT. [8]

Data are currently limited regarding differences in clinical outcome between short-duration and standard-duration DAPT after second-generation DES implantation in the ACS population. Therefore, we performed pooled analysis of three randomized trials to investigate the efficacy and safety of short-duration (≤6 months) compared with standard-duration (≥12 months) of DAPT after second-generation DES implantation in patients with ACS.

## Materials and methods

### Study design

This is a patient-level pooled analysis of data from three multicenter, prospective, open-label, randomized trials comparing short-duration (≤6 months) and standard-duration (≥12 months) DAPT. Each study protocol was previously described. [5, 6, 9] Briefly, the EXCELLENT trial enrolled 1,443 patients with at least 1 *de novo* lesion treated with an everolimus or first-generation sirolimus DES. [5] The RESET trial enrolled 2,117 patients who were randomly assigned in a 1:1 ratio to receive either an Endeavor zotarolimus DES with 3 months of DAPT or a Resolute zotarolimus DES, everolimus DES, or first-generation sirolimus DES with 12 months of DAPT. [6] The IVUS-XPL trial included 1,400 patients who underwent everolimus DES implantation for long coronary lesions (implanted stent length ≥28 mm). [9] The patients were randomly assigned to treatments using a 2 x 2 factorial design according to DAPT duration (6-month vs. 12-month) and whether intravascular ultrasound was used.

In the three trials, ACS was defined as a clinical syndrome consisting of unstable angina, non-ST-elevation myocardial infarction (MI), or ST-elevation MI, diagnosed by clinical presentation, electrocardiogram, or cardiac enzymes. The second-generation DESs were the Resolute zotarolimus DES, everolimus DES, or Endeavor zotarolimus DES. Clopidogrel was the only P2Y12 receptor antagonist used in the DAPT regimens in all studies.

#### Study population

**Fig 1:**
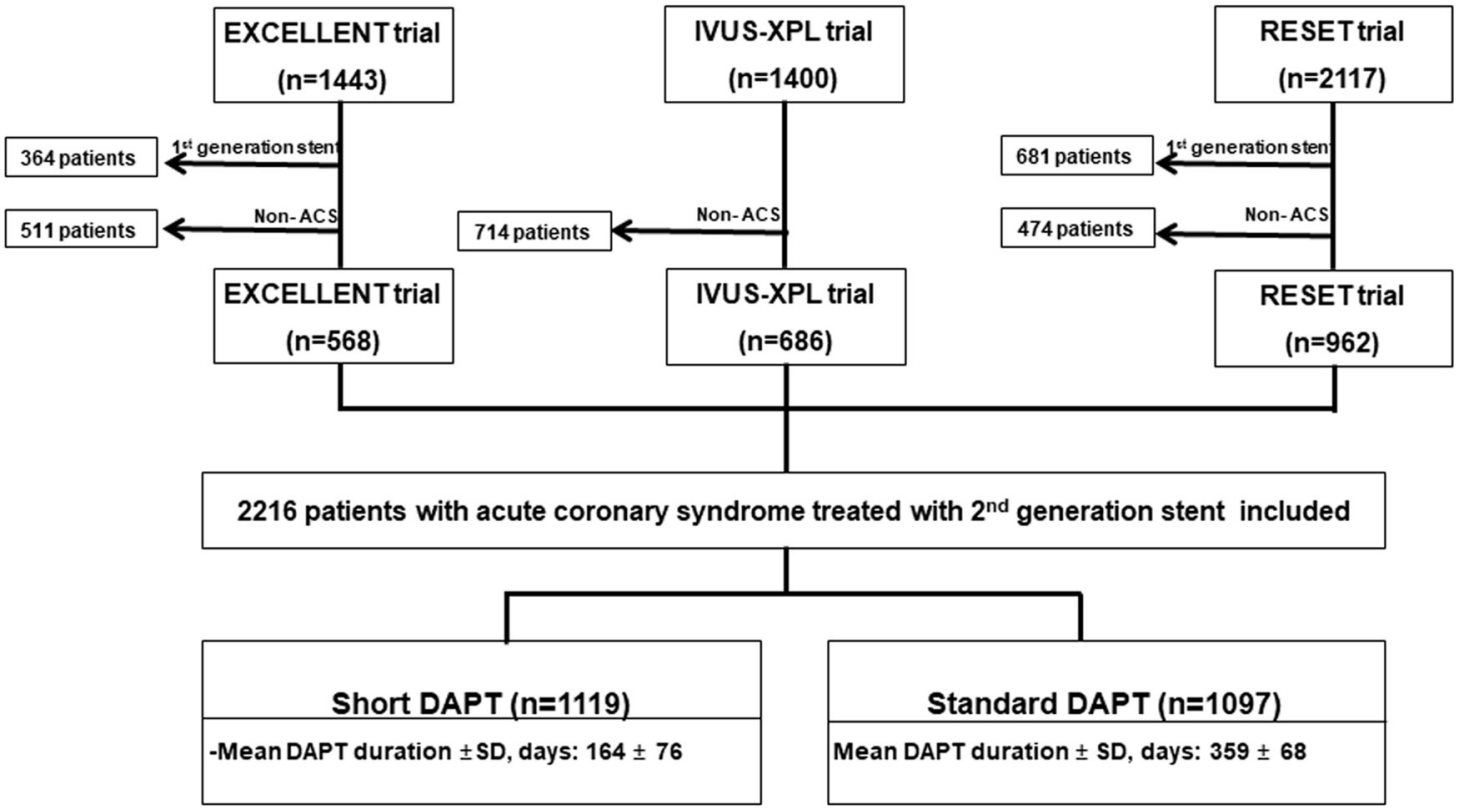
Study profile. From three multicenter, prospective, open-label, randomized trials, a total of 2,216 patients with ACS undergoing second-generation DES implantation divided into short-duration (n = 1119; ≤6 months) and standard-duration (n = 1097; ≥12 months).

The study population included 2,216 patients with ACS: 568 patients from the EXCELLENT trial, 686 patients from the IVUS-XPL trial, and 962 patients from the RESET trial. There were 1,119 patients in the short-duration DAPT arm and 1,097 in the standard-duration DAPT arm (Fig 1).

### Study endpoints

The primary endpoint was composite of cardiac death, MI, stent thrombosis, stroke or major bleeding as net adverse cardiovascular and clinical events during the 12-month period after randomization. Secondary endpoints were composite of cardiac death, MI or stent thrombosis and each component of primary endpoint. Clinical events were defined according to recommendations of the Academic Research Consortium. [10] All deaths were considered cardiac deaths unless a definite non-cardiac cause was established. MI was defined as the presence of clinical signs of MI plus a creatine kinase-MB fraction or troponin T or I increase to above the upper limit of normal. Target vessel revascularization (TVR) was defined as ischemic driven repeat revascularization of the treated vessel by percutaneous coronary intervention (PCI) or bypass surgery. Stent thrombosis was defined as definite or probable stent thrombosis according to the Academic Research Consortium classification. According to the Thrombolysis in Myocardial Infarction (TIMI) criteria, major bleeding was defined as overt clinical bleeding. Stroke was defined according to the definition used in each trial.

### Statistical analysis

Patient-level data were obtained from the principal investigators of each trial. Individual patient data were pooled in a single dataset and analyzed with a single-stage approach. We used the intention-to-treat population for the analyses, with all patients considered according to the treatment arm to which they were randomly assigned. Continuous variables are presented as mean (standard deviation [SD]) and compared using the Student’s t-test. Categorical variables are presented as count (percentage) and compared using χ^2^ test for contingency table with at least five expected cases for cell, or by Fisher exact test for contingency table with fewer than five expected cases for cell. Cumulative event rates were estimated using the Kaplan-Meier method, and survival curves were compared using the log-rank test. Hazard ratios (HRs) with 95% confidence intervals (CIs) were estimated using the Cox proportional hazards method. To minimize bias by including events in the early post-implantation period, landmark analysis for primary endpoint was performed with a landmark of clopidogrel discontinuation at 6 months among patients who were event-free at 6 months (3 months in the RESET trial) with intention to treated analysis.

The consistency of treatment effect in pre-specified subgroups was assessed using Cox regression models with tests for interaction. We applied the DAPT score [11], PRECISE DAPT score [12] and Creatinine Clearance Estimate by Cockcroft-Gault Equation to the study population: DAPT score ≥2, Risk groups based on PRECISE DAPT score, and Creatinine clearance ≤ 60ml/min. A two-sided P-value <0.05 was considered significant. All analyses were performed using SAS version 9.2 (SAS Institute, Cary, NC).

## Results

### Baseline characteristics

Of the 2,216 patients, 1,119 were allocated to receive short-duration DAPT and 1,097 to receive standard-duration DAPT. Mean DAPT durations were 164 ±76 and 359 ±68 days for the two groups. Baseline clinical characteristics of the groups were well balanced (Table 1). Mean age of the entire population was 62.5 ±10.1 years. One-third of the study population presented with an acute MI. The standard-duration DAPT group had a higher number of stents per patient and greater total stent length than the short-duration DAPT group.

**Table 1:** Clinical and Angiographic characteristics.

Characteristics	Short DAPT (n = 1119)	Standard DAPT (n = 1097)	*P*
**Clinical variables**			
DAPT duration, day	164 ± 76	359 ± 68	<0.001
Age, years	62.7 ± 9.7	62.3 ± 10.5	0.349
Male sex	728 (65.1)	741 (67.5)	0.225
Diabetes mellitus	363 (32.4)	360 (32.8)	0.856
Hypertension	705 (63.0)	675 (61.5)	0.483
Dyslipidemia	721 (64.4)	712 (64.9)	0.824
Congestive heart failure	79 (7.1)	76 (6.9)	0.950
Current smoker	320 (28.6)	294 (26.8)	0.481
Clinical presentation at index procedure			0.629
Unstable angina	815 (72.8)	791 (72.1)	
Non ST-elevation MI	262 (23.4)	271 (24.7)	
ST-elevation MI	42 (3.8)	35 (3.2)	
Prior PCI	72 (6.4)	77 (7.0)	0.611
Prior MI	51 (4.6)	34 (3.1)	0.077
LVEF, %	61.2 ± 10.3	61.7 ± 9.8	0.245
DAPT score ≥2*	335 (29.9)	325 (29.6)	0.889
PRECISE DAPT score^†^ groups (n = 2181)			0.181
Very low	343 (31.2)	377 (34.9)	
Low	398 (36.1)	357 (33.1)	
Moderate	215 (19.5)	194 (18.0)	
High	145 (13.2)	152 (14.1)	
**Angiographic variables**			
No. of diseased vessels			0.951
1	526 (47.0)	510 (46.5)	
2	350 (31.3)	343 (31.3)	
3	243 (21.7)	244 (22.2)	
Stent type			
Everolimus-eluting stents	630 (56.3)	749 (68.3)	
Endeavor zotarolimus-eluting stents	489 (43.7)	0 (0)	
Resolute zotarolimus-eluting stents	0 (0)	348 (31.7)	
ACC/AHA class B2/C	809 (72.7)	805 (73.8)	0.564
IVUS-guided implantation	524 (46.8)	512 (46.7)	0.966
No. of stents per patient	1.46 ± 0.72	1.54 ± 0.79	0.015
Stent diameter <3.0 mm	413 (37.1)	406 (37.2)	0.965
Long lesion (≥28 mm)	435 (38.9)	483 (44.1)	0.016
Total stent length, mm	34.2 ±18.1	36.9 ± 20.3	0.001
**Medications at discharge**			
Aspirin	1105 (98.7)	1078 (98.3)	0.572
Clopidogrel	1089 (97.3)	1076 (98.1)	0.201
Beta blocker	770 (68.8)	767 (69.9)	0.676
ACE inhibitor	431 (38.5)	439 (40.0)	0.725
Angiotensin II receptor blocker	330 (29.5)	291 (26.5)	0.258
Statin	1006 (89.9)	958(87.3)	0.154

Data are number (%) or mean ± standard deviation. ACC indicates American College of Cardiology; ACE, angiotensin-converting enzyme; AHA, American Heart Association; CHF, congestive heart failure; IVUS, intravascular ultrasound; LVEF, left ventricular ejection fraction; MI, myocardial infarction; PCI, percutaneous coronary intervention.

*DAPT score was based on the DAPT trial. [11]

^†^ PRECISE DAPT score based on web calculator of PRECISE DAPT. [12]

### Primary and secondary endpoints

**Fig 2:**
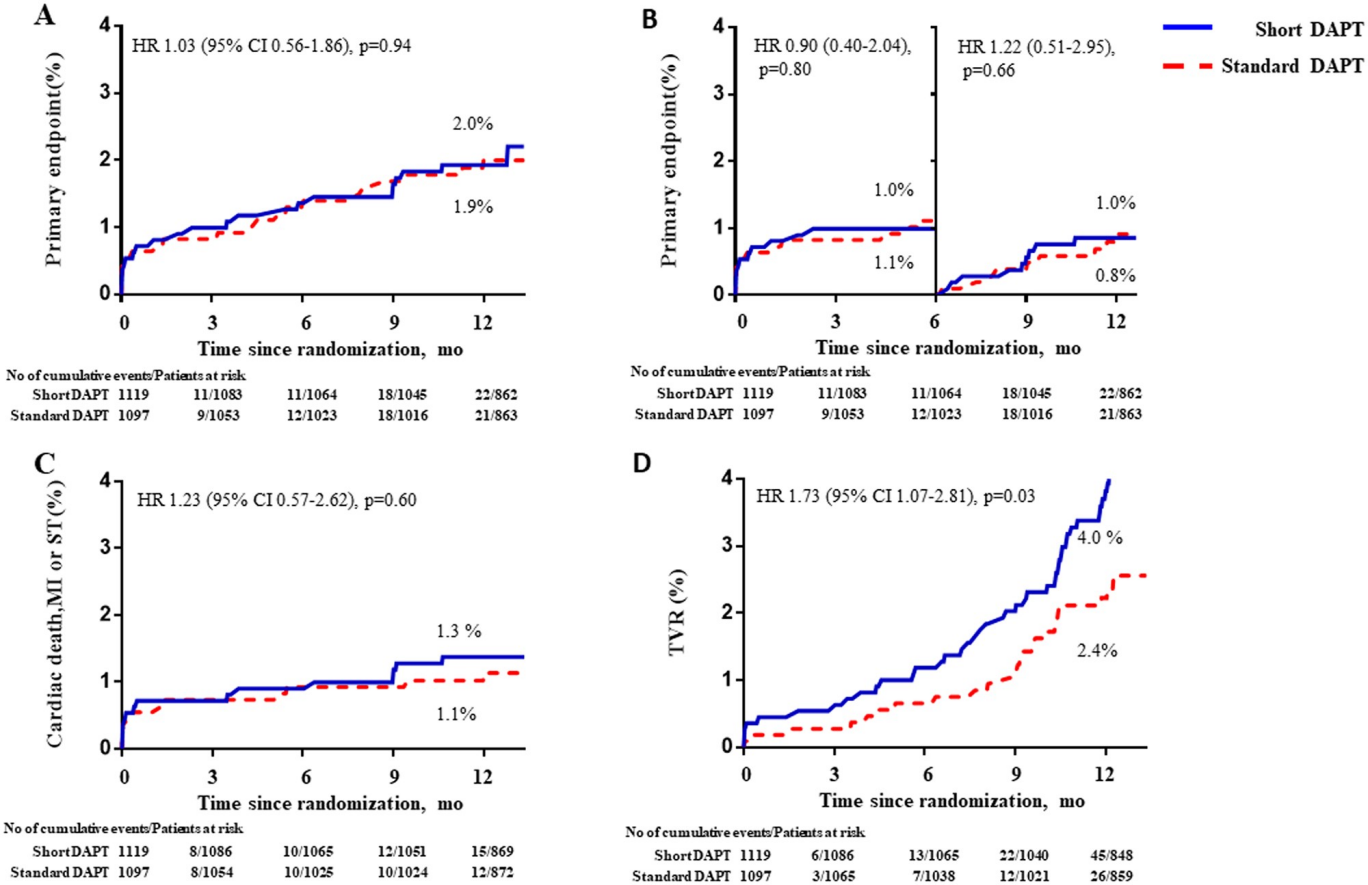
Kaplan-Meier survival curves for the primary and secondary endpoint. **(A)** primary endpoints (Composite of death, MI, stent thrombosis, stroke or major bleeding) in patient with ACS. **(B)** landmark analysis after 6 months. (3 months for RESET). **(C)** Composite of death, MI, stent thrombosis. **(D)** Target vessel revascularization.

Primary endpoints occurred in 22 patients with short-duration DAPT group and 21 patients in standard duration DAPT group (2.0% vs. 1.9%; HR for short-duration DAPT 1.03; 95% CI 0.56–1.86; p = 0.94; Table 2, Fig 2). This result appeared consistently through each trial without heterogeneity (S1 Fig). Landmark analysis at 6 months showed no difference in event rate within 6-month (1.0% vs. 1.1%; HR 0.90; 95% CI 0.40–2.04; p = 0.80) and after 6-months from randomization (1.0% vs. 0.8%; HR 1.22; 95% CI 0.51–2.95; p = 0.66). Composite of cardiac death, MI or stent thrombosis also did not differ according to DAPT duration (1.3% vs. 1.1%; HR 1.23; 95% CI 0.57–2.62; p = 0.60).

**Table 2:** Clinical outcomes during the first 12 months.

	Short DAPT (n = 1119)	Standard DAPT (n = 1097)	HR (95% CI) *	*P*
Primary endpoint^†^	22 (2.0)	21(1.9)	1.03 (0.56–1.86)	0.937
<6 months	11 (1.0)	12 (1.1)	0.90 (0.40–2.04)	0.798
≥6 months	11 (1.0)	9 (0.8)	1.22 (0.51–2.95)	0.657
Cardiac death, MI, or ST	15 (1.3)	12 (1.1)	1.23 (0.57–2.62)	0.600
<6 months	8 (0.7)	10 (0.9)	0.78 (0.31–1.99)	0.609
≥6 months	7 (0.7)	2 (0.2)	3.43 (0.71–16.5)	0.120
All-cause death	9 (0.8)	11 (1.0)	0.80 (0.33–1.92)	0.610
Cardiac death	5 (0.4)	6 (0.5)	0.82 (0.25–2.67)	0.736
MI	7 (0.6)	5 (0.5)	1.37 (0.44–4.32)	0.591
ST	6 (0.5)	4 (0.4)	1.47 (0.42–5.22)	0.549
TVR	45 (4.0)	26 (2.4)	1.73 (1.07–2.81)	0.026
Stroke	3 (0.3)	5 (0.5)	0.58 (0.14–2.44)	0.461
Minor or major bleeding	9 (0.8)	14 (1.3)	0.63 (0.27–1.45)	0.274
Major Bleeding ^‡^	4 (0.4)	6 (0.5)	0.65 (0.18–2.30)	0.503

Data are number (%). CI indicates confidence interval; HR, hazard ratio; ST, stent thrombosis; TVR, target vessel revascularization

* HRs are for short-duration DAPT vs. standard-duration DAPT groups

^†^ Primary endpoint was composite of Cardiac death, MI, ST, stroke or major bleeding.

^‡^ Major bleeding according to Thrombolysis in Myocardial Infarction criteria.

For individual endpoints, the percentages of death, MI, and stent thrombosis were 0.8%, 0.6%, and 0.5% for the short-duration DAPT arm and 1.0%, 0.5%, and 0.4% for the standard-duration DAPT arm (Table 2). Ischemia-driven TVR was required in 45 patients (4.0%) in the short-duration DAPT arm and 26 patients (2.4%) in the standard-duration DAPT arm (HR 1.73; 95% CI 1.07–2.81; p = 0.03). There was no difference in major bleeding between groups (0.4% vs. 0.5%; HR 0.65; 95% CI 0.18–2.30; p = 0.50).

### Subgroup analyses

In patients with MI, although overall incidence of primary endpoints was higher than total group, there was also no difference in primary endpoint (2.6% vs. 2.6%; HR 1.01; 95% CI 0.38–2.69; p = 0.98) and each individual endpoint between two groups (S1 Table). Landmark analysis at 6 months showed no difference (0.7% vs. 0.7%; HR 1.01; 95% CI 0.14–7.19; p = 0.99).

**Fig 3:**
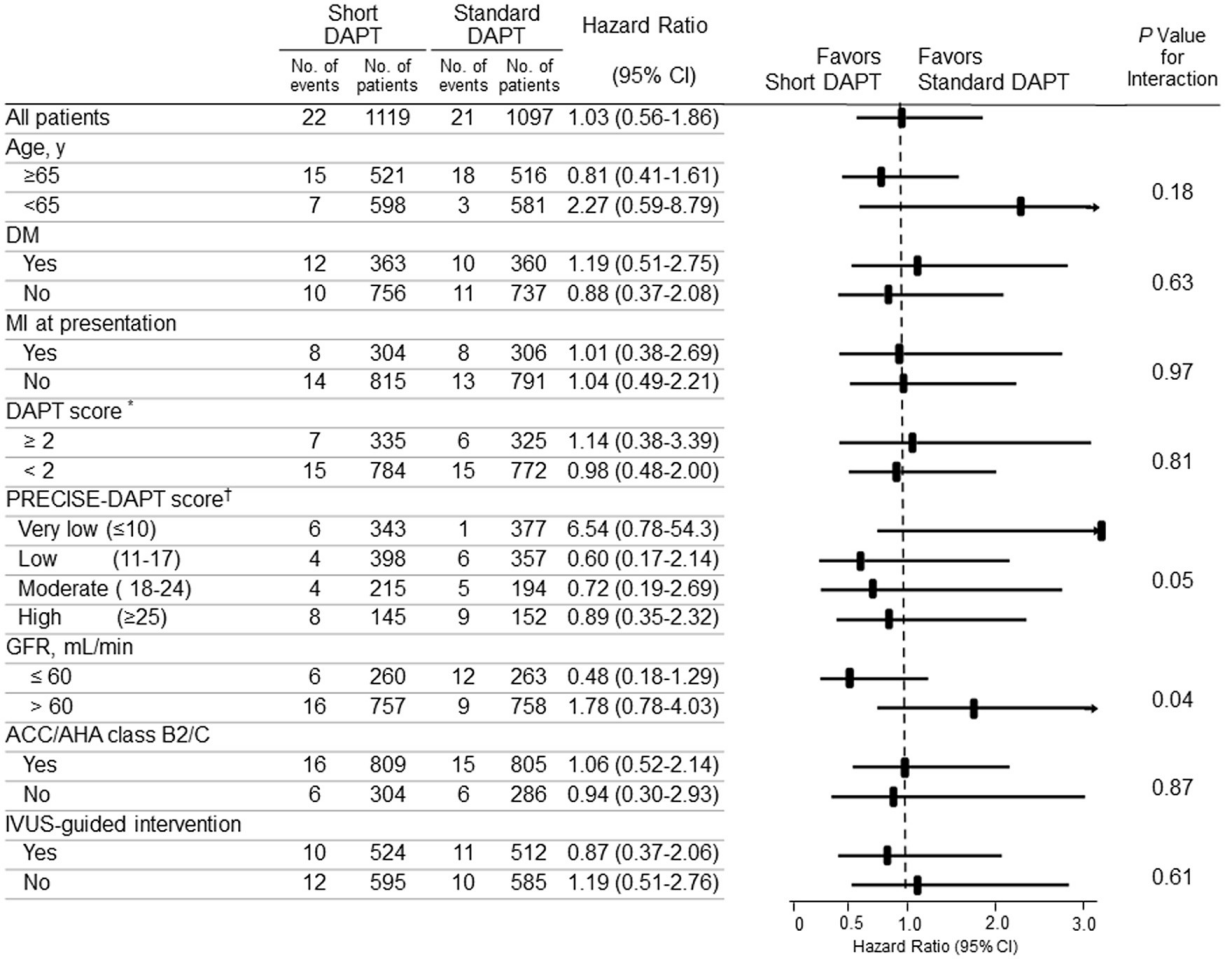
Subgroup analyses of the primary endpoint. ^*^ DAPT score was from the DAPT trial. [11] ^†^ PRECISE DAPT score based on web calculator of PRECISE DAPT;http://www.precisedaptscore.com/ [12]. DM, diabetes mellitus; GFR, glomerular filtration rate.

In pre-specified subgroup analyses, standard duration of DAPT therapy increased primary endpoint within chronic kidney disease (creatinine clearance ≤ 60mL/min) group compared to short duration of DAPT. there was a significant interaction for the chronic kidney disease and duration of DAPT (p for interaction = 0.04, Fig 3).

By contrast, in ‘Very low’ risk group based on PRECISE-DAPT score, standard DAPT therapy showed lower primary endpoint than short DAPT therapy. Other PRECISE-DAPT risk group did not showed any difference. There was a trend of interaction for PRECISE-DAPT risk groups and duration of DAPT. (Fig 3, S2 Table).

## Discussion

In this pooled analysis involving patients with ACS, there is no difference in incidence of net adverse cardiovascular and clinical events (composite of cardiac death, MI, stent thrombosis, stroke or major bleeding) at 12 months after second-generation DES implantation between short (≤6 months) and standard-duration DAPT (≥12 months). However, TVR increased with short-duration DAPT compared with standard duration DAPT.

### DAPT duration in DES area

Although early DESs reduced in-stent restenosis rate compared with bare metal stents, the concern arose that DESs may have a higher incidence of late and very late stent thrombosis. [13] Early discontinuation of DAPT was associated with thrombotic complications in observational studies of first-generation DESs. [14] These concerns reinforced recommendations for longer-duration DAPT after DES implantation.

However, second-generation DESs, with their reduced strut thickness, biocompatible polymer, and appropriate drug dose, has been shown to improve stent strut coverage, reducing stent thrombosis. [15] In the DAPT (Dual Antiplatelet Therapy) trial, a significant interaction was observed between DAPT duration (30 vs. 12 months) and DES type for major adverse cardiovascular and cerebrovascular events (p = 0.048). [16] Moreover, a previous meta-analysis demonstrated that the higher risk of stent thrombosis with short-term DAPT was attenuated with second-generation DESs. [8]

### Previous studies

Several randomized studies compared the safety and efficacy of short-term (≤6 months) versus long-term (≥12 months) DAPT after second-generation DES implantation. The ITALIC trial showed no difference in the primary endpoint (composite of cardiac death, MI, stroke, TVR, or major bleeding) at 12 months between 6-month and 24-month DAPT after everolimus eluting DES implantation. [17] The TVR was higher with 6-month DAPT, although the difference did not reach statistical significance. Notable, the ITALIC trial enrolled primarily low-risk patients; most had stable angina, and only a limited number had ACS. The OPTIMIZE trial demonstrated that 3-month DAPT in patients with stable angina and low-risk ACS was associated with a similar rate of major adverse cardiac events (composite of all-cause death, MI or TVR) compared with 12-month DAPT. [4] It also showed non-inferiority of short-duration DAPT compared with 12-month DAPT for firm endpoints, such as the composite of all-cause death, MI, stroke, or major bleeding. Recently, the SECURITY trial tested the non-inferiority of 6-month DAPT in patients with stable angina and unstable angina following the implantation of various second-generation DESs. [3] The composite of cardiac death, MI, stroke, stent thrombosis, or major bleeding did not differ between groups. Although previous studies showed consistent non-inferiority of short-duration (≤6 months) compared to standard duration (≥12 months) DAPT with second-generation DESs, these results may not be applicable to high-risk populations because most of the studies included patients with stable angina and a low percentage with ACS.

### Short DAPT duration in ACS with second generation DES

ACS itself is a risk factor for future thrombotic cardiovascular events, and 12 months of DAPT has been recommended in practice. [1] However, potent antiplatelet therapy increases the risk of bleeding complications. A recent meta-analysis of 11,473 patients receiving a DES indicated that the benefits of different DAPT durations varied according to clinical presentation. [10] In ACS patients, short-duration (≤6 months) DAPT showed a higher trend in 1-year rate of MI or stent thrombosis compared with standard duration (≥12 months) DAPT (2.43% vs. 1.67%; p = 0.06). Conversely, MI or stent thrombosis rates were comparable between DAPT groups in patients with stable angina (1.67% vs. 1.79%; p = 0.72). Recent SMART-DATE trial evaluating short-duration DAPT after second-generation DES implantation in ACS showed non-inferiority of short-duration DAPT in composite of all-cause death, MI or stroke at 18 months. [18] However, there was an increased incidence of MI in short-duration DAPT. These results suggested that an individualized DAPT regimen and duration are needed based on the risk of ischemia and bleeding in clinical setting. Furthermore, the ischemic and bleeding risk may be different among patients with ACS. Therefore, decision regarding DAPT duration are more complicated and need a shared decision to achieve better net clinical outcome. [19]

Our study demonstrated that short-duration (≤6 months) DAPT was associated with a similar rate of net adverse cardiovascular and clinical events in patients with ACS (including MI) treated with second-generation DESs. Landmark analysis showed no divergence of the curves for primary endpoint after 6 months following stent implantation between short-duration and standard-duration DAPT. The cumulative low incidence of stent thrombosis or MI after second-generation DES implantation might have contributed to these results, suggesting that short-duration (≤6 months) DAPT might be applied for selected patients with low ischemic risk of ACS after second-generation DESs. Although some discrepant results were observed regarding the efficacy and safety of long-term DAPT after DES implantation, recent PCI guidelines indicate that 6-month DAPT is recommended after second-generation DES implantation for patients with ACS and those with a higher bleeding risk. [1] The current pooled analysis provides useful information regarding optimal DAPT duration in patients with ACS after second-generation DES implantation, although our results should be interpreted with caution according to each clinical situation.

In a subgroup analysis, significant interaction was shown between chronic kidney disease and duration of DAPT. Chronic kidney disease is well known to be a risk factor for both ischemic and bleeding clinical events. [20] For net clinical and cardiovascular benefit, standard duration of DAPT could be inferior compared to short-duration of DAPT.

Based on PRECISE-DAPT score, ‘very low’ bleeding risk group showed lower net clinical and cardiovascular events in standard-duration of DAPT compared to short-duration of DAPT in our study. These results might suggest longer DAPT for low bleeding risk with high ischemic risk group such as ACS. But, overall borderline interaction with the PRECISE-DAPT score indicated that a personalized approach might be the best option to optimize a clinical outcome.

Current guideline recommended to decide duration of DAPT based on bleeding risk score system that is not enough to say ‘personalized DAPT’. [1] To develop a prediction model more accurately, in additional to traditional risk factors such as diabetes, complexity of coronary disease, epigenetic novel biomarkers may play a role to differentiate an inter-individual platelet reactivity, disease susceptibility or response to therapy. [21] For an association between a platelet activity and diabetes, several studies indicated an interaction between DAPT duration and diabetes in terms of clinical outcomes in first-generation DESs. [22] However, this interaction was not shown in second-generation DESs. [3] In our subgroup analysis, there was a no significant difference in clinical event between patient with diabetes and non-diabetes. Although diabetes is well known to be associated with an increased platelet activity, [23] more investigation is needed to prove whether diabetes itself could interact with DAPT duration for clinical events after second-generation DES implantation.

Coronary artery disease is complex and broad-spectrum disease that many factors should be taken into consideration. However, prediction model based on traditional mathematical model is too complex to apply in clinical practice. Artificial neural networks and sustainable machine learning systems might be helpful in solving dilemmas of optimal DAPT duration raised by our meta-analysis. [24] However, more advanced development and validation are need to apply for clinical practice.

### Study limitation

This study has limitations. First, sample size of the study population might be not sufficient to evaluate the safety of short-duration DAPT compared to longer-duration DAPT in terms of hard clinical endpoints such as death, MI, or stent thrombosis. Second, all 3 trials were open-label and not placebo-controlled studies; however, the possibility of bias was minimized in each trial by using precisely-defined criteria for the primary endpoint, blinding the adjudication by event-adjudication committee members, and analyzing the data using intention-to-treat methodology. Third, short-duration DAPT was 3 months (RESET) or 6 months (Excellent and XPL-IVUS), and the Endeavor zotarolimus-eluting stent was included in the 3-month DAPT group in the RESET study. Fourth, clopidogrel was used with aspirin as DAPT in all 3 trials, so we could not evaluate the effects of DAPT duration for other antiplatelet agents, such as ticagrelor or prasugrel. Fifth, 1 year of clinical follow-up may not be sufficient to assess late outcomes, especially very late stent thrombosis. Nevertheless, only a few existing clinical studies have been performed with adequate number of populations for optimal duration of DAPT in ACS patients. In this perspective, our pooled patient-level analysis may provide an insight to how to apply an optimal duration of DAPT in current DES era with reasonable number of patient population with ACS.

### Conclusions

In patient-level pooled analysis, short-duration (≤6 months) DAPT showed no difference in incidence of net adverse cardiovascular and clinical events, including cardiac death, MI, stent thrombosis, stroke or major bleeding at 12 months after second-generation DES implantation in patients with ACS compared with standard-duration (≥12 months) DAPT. Composite of cardiac death, MI or stent thrombosis or each component also did not differ according to DAPT duration. These findings should be confirmed with large randomized clinical trials.

## Supporting information

S1 Fig Forest plot for primary endpoint with random effect model. (TIF)

S1 Table Clinical outcomes during the first 12 months in acute myocardial infarction. (PDF)

S2 Table Clinical outcomes during the first 12 months according to PRECSISE DAPT score groups. (PDF)

S1 File Original article of RESET trial. (PDF)

S2 File Original article of EXCELLENT trial. (PDF)

S3 File Original article of XPL-IVUS trial. (PDF)

S4 File Pooled analysis data. (CSV)

## References

[1] Valgimigli M, Bueno H, Byrne RA, Collet JP, Costa F, Jeppsson A, et al 2017 ESC focused update on dual antiplatelet therapy in coronary artery disease developed in collaboration with EACTS: The Task Force for dual antiplatelet therapy in coronary artery disease of the European Society of Cardiology (ESC) and of the European Association for Cardio-Thoracic Surgery (EACTS). Eur Heart J. 2017:00:1–48

[2] Palmerini T, Benedetto U, Biondi-Zoccai G, Della Riva D, Bacchi-Reggiani L, Smits PC, et al Long-Term Safety of Drug-Eluting and Bare-Metal Stents: Evidence From a Comprehensive Network Meta-Analysis. J Am Coll Cardiol. 2015;65:2496–2507. 10.1016/j.jacc.2015.04.017 26065988 10.1016/j.jacc.2015.04.017

[3] Colombo A, Chieffo A, Frasheri A, Garbo R, Masotti-Centol M, Salvatella N, et al Second-generation drug-eluting stent implantation followed by 6- versus 12-month dual antiplatelet therapy: the SECURITY randomized clinical trial. J Am Coll Cardiol. 2014;64:2086–2097. 10.1016/j.jacc.2014.09.008 25236346 10.1016/j.jacc.2014.09.008

[4] Feres F, Costa RA, Abizaid A, Leon MB, Marin-Neto JA, Botelho RV, et al Three vs twelve months of dual antiplatelet therapy after zotarolimus-eluting stents: the OPTIMIZE randomized trial. JAMA 2013;310:2510–2522. 10.1001/jama.2013.282183 24177257 10.1001/jama.2013.282183

[5] Gwon HC, Hahn JY, Park KW, Song YB, Chae IH, Lim DS, et al Six-month versus 12-month dual antiplatelet therapy after implantation of drug-eluting stents: the Efficacy of Xience/Promus Versus Cypher to Reduce Late Loss After Stenting (EXCELLENT) randomized, multicenter study. Circulation. 2012;125:505–513. 10.1161/CIRCULATIONAHA.111.059022 22179532 10.1161/CIRCULATIONAHA.111.059022

[6] Kim BK, Hong MK, Shin DH, Nam CM, Kim JS, Ko YG, et al A new strategy for discontinuation of dual antiplatelet therapy: the RESET Trial (REal Safety and Efficacy of 3-month dual antiplatelet Therapy following Endeavor zotarolimus-eluting stent implantation). J Am Coll Cardiol. 2012;60:1340–1348. 10.1016/j.jacc.2012.06.043 22999717 10.1016/j.jacc.2012.06.043

[7] Palmerini T, Della Riva D, Benedetto U, Bacchi Reggiani L, Feres F, Abizaid A, et al Three, six, or twelve months of dual antiplatelet therapy after DES implantation in patients with or without acute coronary syndromes: an individual patient data pairwise and network meta-analysis of six randomized trials and 11 473 patients. Eur Heart J. 2017;38:1034–1043. 10.1093/eurheartj/ehw627 28110296 10.1093/eurheartj/ehw627 PMC5837418

[8] Giustino G, Baber U, Sartori S, Mehran R, Mastoris I, Kini AS, et al Duration of dual antiplatelet therapy after drug-eluting stent implantation: a systematic review and meta-analysis of randomized controlled trials. J Am Coll Cardiol. 2015;65:1298–1310. 10.1016/j.jacc.2015.01.039 25681754 10.1016/j.jacc.2015.01.039

[9] Hong SJ, Shin DH, Kim JS, Kim BK, Ko YG, Choi D, et al 6-Month Versus 12-Month Dual-Antiplatelet Therapy Following Long Everolimus-Eluting Stent Implantation: The IVUS-XPL Randomized Clinical Trial. JACC Cardiovasc Interv. 2016;9:1438–1446. 10.1016/j.jcin.2016.04.036 27212028 10.1016/j.jcin.2016.04.036

[10] Cutlip DE, Windecker S, Mehran R, Boam A, Cohen DJ, van Es GA, et al Clinical end points in coronary stent trials: a case for standardized definitions. Circulation. 2007;115:2344–2351. 10.1161/CIRCULATIONAHA.106.685313 17470709 10.1161/CIRCULATIONAHA.106.685313

[11] Kereiakes DJ, Yeh RW, Massaro JM, Cutlip DE, Steg PG, Wiviott SD, et al DAPT Score Utility for Risk Prediction in Patients With or Without Previous Myocardial Infarction. J Am Coll Cardiol. 2016;67:2492–2502. 10.1016/j.jacc.2016.03.485 27046159 10.1016/j.jacc.2016.03.485

[12] Costa F, van Klaveren D, James S, Heg D, Raber L, Feres F, et al Derivation and validation of the predicting bleeding complications in patients undergoing stent implantation and subsequent dual antiplatelet therapy (PRECISE-DAPT) score: a pooled analysis of individual-patient datasets from clinical trials. Lancet 2017;389:1025–34 10.1016/S0140-6736(17)30397-5 28290994 10.1016/S0140-6736(17)30397-5

[13] Stone GW, Moses JW, Ellis SG, Schofer J, Dawkins KD, Morice MC, et al Safety and efficacy of sirolimus- and paclitaxel- eluting coronary stents. N Engl J Med. 2007;356: 998–1008. 10.1056/NEJMoa067193 17296824 10.1056/NEJMoa067193

[14] Pfisterer M, Brunner-La Rocca HP, Rickenbacher P, Hunziker P, Mueller C, et al Late clinical events after clopidogrel discontinuation may limit the benefit of drug-eluting stents: an observational study of drug-eluting versus bare-metal stents. J Am Coll Cardiol. 2006;48:2584–2591. 10.1016/j.jacc.2006.10.026 17174201 10.1016/j.jacc.2006.10.026

[15] Raber L, Magro M, Stefanini GG, Kalesan B, van Domburg RT, Onuma Y, et al Very late coronary stent thrombosis of a newer-generation everolimus-eluting stent compared with early-generation drug-eluting stents: a prospective cohort study. Circulation. 2012;125:1110–1121. 10.1161/CIRCULATIONAHA.111.058560 22302840 10.1161/CIRCULATIONAHA.111.058560

[16] Mauri L, Kereiakes DJ, Yeh RW, Driscoll-Shempp P, Cutlip DE, Steg PG, et al Twelve or 30 Months of Dual Antiplatelet Therapy after Drug-Eluting Stents. N Engl J Med. 2014;371:2155–2166. 10.1056/NEJMoa1409312 25399658 10.1056/NEJMoa1409312 PMC4481318

[17] Gilard M, Barragan P, Noryani AA, Noor HA, Majwal T, Hovasse T, et al 6- versus 24-month dual antiplatelet therapy after implantation of drug-eluting stents in patients nonresistant to aspirin: the randomized, multicenter ITALIC trial. J Am Coll Cardiol. 2015; 65:777–786. 10.1016/j.jacc.2014.11.008 25461690 10.1016/j.jacc.2014.11.008

[18] Hahn JY, Song YB, Oh JH, Cho DK, Lee JB, Doh JH, et al 6-month versus 12-month or longer dual antiplatelet therapy after percutaneous coronary intervention in patients with acute coronary syndrome (SMART-DATE): a randomised, open-label, non-inferiority trial. Lancet. 2018;391:1274–1284 10.1016/S0140-6736(18)30493-8 29544699 10.1016/S0140-6736(18)30493-8

[19] Chaturvedula S, Diver D, Vashist A. Antiplatelet Therapy in Coronary Artery Disease: A Daunting Dilemma. J Clin Med. 2018;7(4). 10.3390/jcm7040074 PMC5920448 29642547

[20] Baber U, Mehran R, Giustino G, Cohen DJ, Henry TD, Sartori S, et al Coronary Thrombosis and Major Bleeding After PCI With Drug-Eluting Stents: Risk Scores From PARIS. J Am Coll Cardiol. 2016; 67:2224–2234 10.1016/j.jacc.2016.02.064 27079334 10.1016/j.jacc.2016.02.064

[21] Pordzik J, Pisarz K, De Rosa S, Jones AD, Eyileten C, Indolfi C, et al Malek L, Postula M. The Potential Role of Platelet-Related microRNAs in the Development of Cardiovascular Events in High-Risk Populations, Including Diabetic Patients: A Review. Front Endocrinol. 2018;9:74. 10.3389/fendo.2018.00074 PMC5869202 29615970

[22] Thukkani AK, Agrawal K, Prince L, Smoot KJ, Dufour AB, Cho K, et al Long-Term Outcomes in Patients With Diabetes Mellitus Related to Prolonging Clopidogrel More Than 12 Months After Coronary Stenting. J Am Coll Cardiol. 2015;66(10):1091–1101. 10.1016/j.jacc.2015.06.1339 26337986 10.1016/j.jacc.2015.06.1339 PMC4578293

[23] Pechlivani N, Ajjan RA. Thrombosis and Vascular Inflammation in Diabetes: Mechanisms and Potential Therapeutic Targets. Front Cardiovasc Med. 2018;5:1 10.3389/fcvm.2018.00001 29404341 10.3389/fcvm.2018.00001 PMC5780411

[24] Podda GM, Grossi E, Palmerini T, Buscema M, Femia EA, Della Riva D, et al Prediction of high on-treatment platelet reactivity in clopidogrel-treated patients with acute coronary syndromes. Int J cardiol. 2017;240:60–65. 10.1016/j.ijcard.2017.03.074 28343766 10.1016/j.ijcard.2017.03.074

