# Supplementary figures and images for: Optimal duration of DAPT after second-generation drug-eluting stent in acute coronary syndrome
Source: PLoS One. 2018 Nov 26;13(11):e0207386. doi: 10.1371/journal.pone.0207386 (PMC6261023; doi:10.1371/journal.pone.0207386)

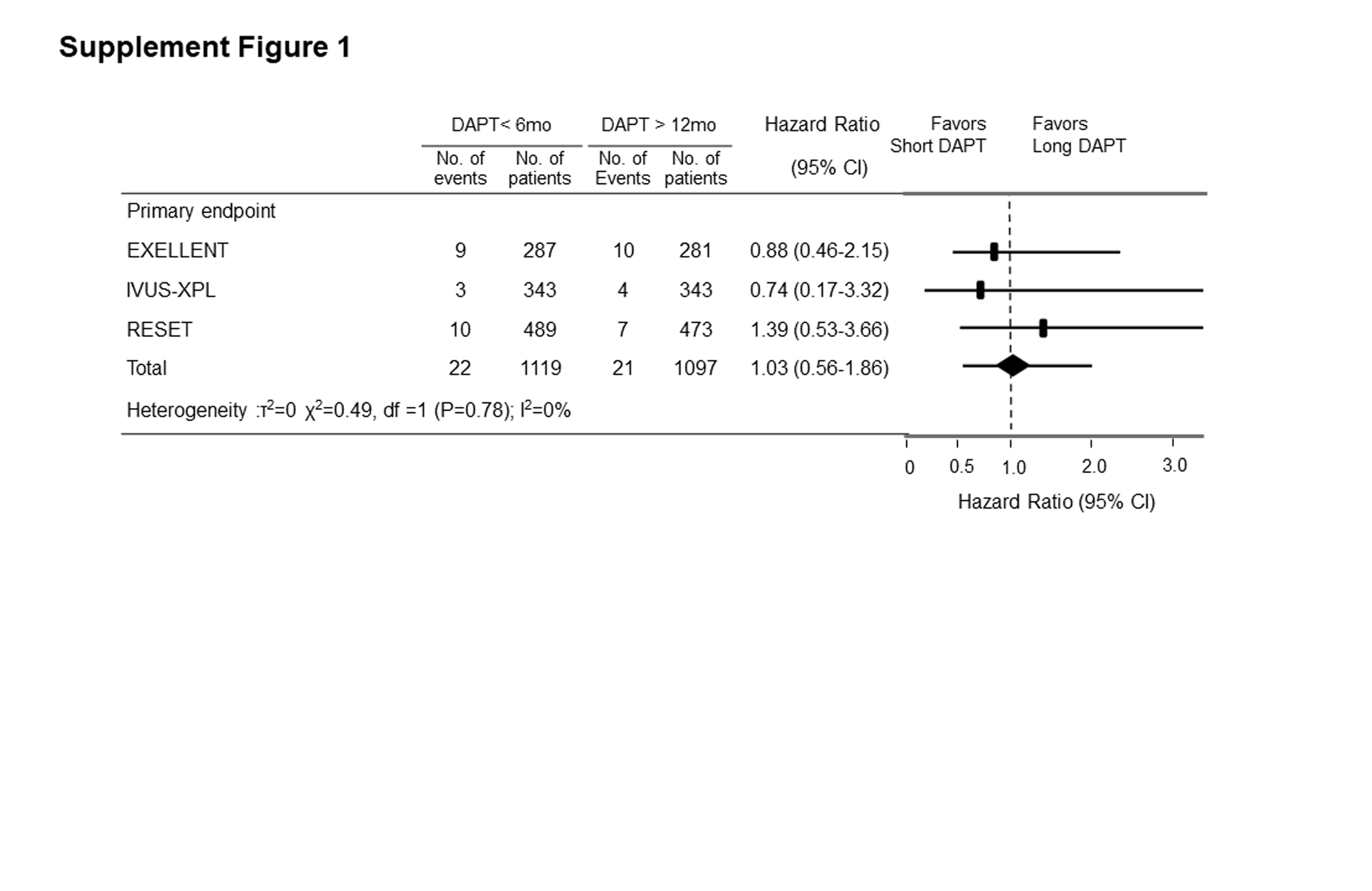

Supplement: S1 Fig — (TIF) [file pone.0207386.s001.tif]
