# Supplementary material for: Optimal duration of DAPT after second-generation drug-eluting stent in acute coronary syndrome
Source: PLoS One. 2018 Nov 26;13(11):e0207386. doi: 10.1371/journal.pone.0207386 (PMC6261023; doi:10.1371/journal.pone.0207386)
Supplement: S1 Table — (PDF) [file pone.0207386.s002.pdf]

Supplement Table 1. Clinical Outcomes During the First 12 Months in Acute Myocardial Infarction.

	Short DAPT (n=304)	Standard DAPT (n=306)	HR (95% CI) *	*P*-value
Primary endpoint †	8 (2.6)	8 (2.6)	1.01 (0.38-2.69)	0.98
<6 months	6 (1.9)	6 (1.9)	1.01 (0.33-3.14)	0.98
≥6 months	2 (0.7)	2 (0.7)	1.01 (0.14-7.19)	0.99
Cardiac death, MI, or ST	6 (2.0)	7 (2.3)	0.87 (0.29-2.58)	0.80
<6 months	5 (1.6)	5 (1.6)	1.01 (0.29-3.49)	0.98
≥6 months	1 (0.4)	2 (0.7)	0.51 (0.05-5.59)	0.58
All-cause death	4 (1.3)	4 (1.3)	1.01 (0.25-4.06)	0.98
Cardiac death	2 (0.7)	4 (1.3)	0.50 (0.09-2.75)	0.43
MI	1 (0.3)	3 (1.0)	0.34 (0.04-3.23)	0.34
ST	4 (1.3)	2 (0.7)	2.03 (0.37-11.1)	0.41
TVR	15 (4.9)	11 (3.6)	1.39 (0.64-3.02)	0.41
Stroke	1 (0.3)	1 (0.3)	1.01 (0.07-16.1)	0.99
Minor or major bleeding	3 (1.0)	3 (1.0)	1.01 (0.20-5.01)	0.99
Major Bleeding ‡	1 (0.3)	0 (0.0)		0.61

Data are number (%).* HRs are for short-duration DAPT vs. standard-duration DAPT groups

† Primary endpoint was composite of Cardiac death, MI, ST, stroke or major bleeding.

‡ Major bleeding according to Thrombolysis in Myocardial Infarction criteria.[15]
