# Supplementary material for: Optimal duration of DAPT after second-generation drug-eluting stent in acute coronary syndrome
Source: PLoS One. 2018 Nov 26;13(11):e0207386. doi: 10.1371/journal.pone.0207386 (PMC6261023; doi:10.1371/journal.pone.0207386)
Supplement: S2 Table — (PDF) [file pone.0207386.s003.pdf]

Supplement Table 2. Clinical Outcomes During the First 12 Months according to PRECISE DAPT score groups.

	Short DAPT	Standard DAPT	p	HR (95%CI) *	p†
**Not available PRECISE-DAPT**	18	17			
**Very low risk (≤10) group (n=720)**	343	377			
MI, ST, or Stroke	5(1.5)	1(0.3)	0.08	5.45(0.64-46.7)	0.12
MI, ST, Stroke or TVR	22 (6.4)	10 (2.7)	0.01	2.47(1.17-5.22)	0.02
Minor or Major bleeding	0	2 (0.5)	0.18	NA	
Cardiac death, MI, ST, stroke or Major bleeding	6 (1.7)	1 (0.3)	0.04	6.54 (0.79-54.3)	0.08
Cardiac death, MI, ST, stroke, Major bleeding or TVR	23 (6.7)	10 (2.7)	0.01	2.58(1.23-5.42)	0.01
**Low risk (11~17) groups (n=755)**	398	357			
MI, ST, or Stroke	4 (1.0)	5 (1.4)	0.63	0.73(0.19-2.70)	0.63
MI, ST, Stroke or TVR	17 (4.3)	11 (3.1)	0.36	1.42(0.66-3.03)	0.37
Minor or Major bleeding	4 (1.0)	4 (1.1)	0.90	0.91(0.23-3.65)	0.90
Cardiac death, MI, ST, stroke or Major bleeding	4 (1.0)	6 (1.7)	0.43	0.60 (0.17-2.14)	0.44
Cardiac death, MI, ST, stroke, Major bleeding or TVR	17 (4.3)	12 (3.4)	0.49	1.30 (0.62-2.72)	0.49
**Moderate risk (18~24) groups(n=409)**	215	194			
MI, ST, or Stroke	3 (1.4)	4 (2.1)	0.61	0.68 (0.15-3.04)	0.61
MI, ST, Stroke or TVR	10 (4.7)	7 (3.6)	0.59	1.30(0.49-3.41)	0.60
Minor or Major bleeding	0	2 (1.0)	0.14	NA	
Cardiac death, MI, ST, stroke or Major bleeding	4 (1.9)	5 (2.6)	0.63	0.72 (0.19-2.69)	0.72
Cardiac death, MI, ST, stroke, Major bleeding or TVR	11 (5.1)	8 (4.1)	0.64	1.25(0.50-3.09)	0.64
**High risk (≥25) groups (n=297)**	145	152			
MI, ST, or Stroke	2 (1.4)	3 (2.0)	0.67	0.68(0.11-4.08)	0.67
MI, ST, Stroke or TVR	3 (2.1)	6 (3.9)	0.32	0.50(0.13-2.00)	0.33
Minor or Major bleeding	5 (3.4)	6 (4.0)	0.75	0.83 (0.25-2.71)	0.75
Cardiac death, MI, ST, stroke or Major bleeding	8 (5.5)	9 (5.9)	0.82	0.89(0.35-2.32)	0.82

Cardiac death, MI, ST, stroke, Major bleeding or TVR	9 (6.2)	12 (7.9)	0.51	0.75(0.31-1.77)	0.51

Data are number (%).* HRs are for short-duration DAPT vs. standard-duration DAPT groups. †P was calculated with Cox proportional hazard regression models.
