# Supplementary material for: Optimal duration of DAPT after second-generation drug-eluting stent in acute coronary syndrome
Source: PLoS One. 2018 Nov 26;13(11):e0207386. doi: 10.1371/journal.pone.0207386 (PMC6261023; doi:10.1371/journal.pone.0207386)
Supplement: S1 File — (PDF) [file pone.0207386.s004.pdf]

Journal of the American College of Cardiology
© 2012 by the American College of Cardiology Foundation
Published by Elsevier Inc.

Vol. 60, No. 15, 2012
ISSN 0735-1097/$36.00
http://dx.doi.org/10.1016/j.jacc.2012.06.043

# A New Strategy for Discontinuation of Dual Antiplatelet Therapy

## The RESET Trial (REal Safety and Efficacy of 3-month dual antiplatelet Therapy following Endeavor zotarolimus-eluting stent implantation)

Byeong-Keuk Kim, MD,* Myeong-Ki Hong, MD,*† Dong-Ho Shin, MD, MPH,* Chung-Mo Nam, PHD,‡ Jung-Sun Kim, MD,* Young-Guk Ko, MD,* Donghoon Choi, MD,* Tae-Soo Kang, MD,§ Byoung-Eun Park, MD,§ Woong-Chol Kang, MD,‖ Seung-Hwan Lee, MD,¶ Jung-Han Yoon, MD,¶ Bum-Kee Hong, MD,# Hyuck-Moon Kwon, MD,# Yangsoo Jang, MD,*† for the RESET Investigators

*Seoul, Cheonan, Incheon, and Wonju, Republic of Korea*

**Objectives** The goal of this study was to evaluate shorter duration (3 months) dual antiplatelet therapy (DAPT) after drug-eluting stent (DES) implantation.

**Background** There have been few published reports of prospective randomized clinical studies comparing the safety and efficacy of shorter duration DAPT after DES implantation.

**Methods** We randomly assigned 2,117 patients with coronary artery stenosis into 2 groups according to DAPT duration and stent type: 3-month DAPT following Endeavor zotarolimus-eluting stent (E-ZES) implantation (E-ZES+3-month DAPT, n = 1,059) versus 12-month DAPT following the other DES implantation (standard therapy, n = 1,058). We hypothesized that the E-ZES+3-month DAPT would be noninferior to the standard therapy for the primary composite endpoint (cardiovascular death, myocardial infarction, stent thrombosis, target\vessel revascularization, or bleeding) at 1 year.

**Results** The primary endpoint occurred in 40 (4.7%) patients assigned to E-ZES+3-month DAPT compared with 41 (4.7%) patients assigned to the standard therapy (difference: 0.0%; 95% confidence interval [CI]: −2.5 to 2.5; p = 0.84; p < 0.001 for noninferiority). The composite rates of any death, myocardial infarction, or stent thrombosis were 0.8% and 1.3%, respectively (difference: −0.5%; 95% CI: −1.5 to 0.5; p = 0.48). The rates of stent thrombosis were 0.2% and 0.3%, respectively (difference: −0.1%; 95% CI: −0.5 to 0.3; p = 0.65) without its further occurrence after cessation of clopidogrel in the E-ZES+3-month DAPT group. The rates of target vessel revascularization were 3.9% and 3.7%, respectively (difference: 0.2%; 95% CI: −2.3 to 2.6; p = 0.70).

**Conclusions** E-ZES+3-month DAPT was noninferior to the standard therapy with respect to the occurrence of the primary endpoint. (REal Safety and Efficacy of a 3-month dual antiplatelet Therapy following E-ZES implantation [RESET]; NCT01145079) (J Am Coll Cardiol 2012;60:1340–8) © 2012 by the American College of Cardiology Foundation

Because one of the strong predictors for stent thrombosis is early discontinuation of clopidogrel (1,2), prolonged dual antiplatelet therapy (DAPT) is highly recommended to prevent stent thrombosis (1,3). However, reports from several trials of the zotarolimus-eluting stent (Endeavor [E-ZES], Medtronic, Santa Rosa, California) have shown

See page 1349

From the *Severance Cardiovascular Hospital, Yonsei University College of Medicine, Seoul, Korea; †Severance Biomedical Science Institute, Yonsei University College of Medicine, Seoul, Korea; ‡Department of Preventive Medicine and Biostatistics, Yonsei University College of Medicine, Seoul, Korea; §Dankook University College of Medicine, Cheonan, Korea; ‖Gachon University College of Medicine, Incheon, Korea; ¶Yonsei University Wonju College of Medicine, Wonju, Korea; and the #Kangnam Severance Hospital, Seoul, Korea. This study was supported by the Cardiovascular Research Center, Seoul, Korea, Medtronic Inc., and grants from the Korea Healthcare Technology R&D Project, Ministry for Health, Welfare & Family Affairs, Republic of Korea (No. A085012 and A102064), and the Korea Health 21 R&D Project, Ministry of Health & Welfare, Republic of Korea (No. A085136). Dr. Myeong-Ki Hong has received research grants from Medtronic and the Cardiovascular Research Centers, Seoul, Korea. Dr. Jang is a consultant to and has received research funds from Medtronic. All other authors have reported that they have no relationships relevant to the contents of this paper to disclose.

Manuscript received April 2, 2012; revised manuscript received June 9, 2012, accepted June 12, 2012.

beneficial efficacy and safety, despite a relatively short duration of DAPT (4–6). One optical coherence tomography study reported sufficient strut coverage following implantation with the E-ZES as early as 3 months post-procedure (7). A recent registry study with 661 low-risk patients who received DAPT for 3 months following E-ZES implantation showed favorable long-term clinical outcomes and lower incidence of stent thrombosis after cessation of clopidogrel 3 months post-intervention (8). On the basis of the safety nature of E-ZES and theoretical backgrounds from the imaging and clinical studies, we hypothesized that 3-month DAPT after E-ZES implantation (E-ZES+3-month DAPT) may be noninferior to 12-month DAPT after implantation with other drug-eluting stent (DES) (standard therapy). In the RESET (REal Safety and Efficacy of a 3-month dual antiplatelet Therapy following E-ZES implantation) trial, we compared the safety and efficacy between patients treated with E-ZES+3-month DAPT and patients treated with the standard therapy.

## Methods

The RESET trial was a prospective, open-label, randomized trial conducted at 26 sites in Korea; the complete lists and detailed information regarding participating institutes appear in the Online Appendix. The trial protocol was approved by the institutional review board at each participating center.

Patients with a diagnosis of angina or acute myocardial infarction with more than 50% diameter stenosis in a coronary artery by visual estimation, who presented to the catheterization laboratory for elective percutaneous coronary intervention, were eligible for participation. The complete inclusion and exclusion criteria are provided in the Online Appendix. All study participants provided written informed consent using documents approved by the local ethics board.

**Abbreviations and Acronyms**

**CI** = confidence interval

**DAPT** = dual antiplatelet therapy

**DES** = drug-eluting stent(s)

**E-ZES** = Endeavor zotarolimus-eluting stent(s)

Using an interactive web-based response system, study participants were randomly assigned in a 1:1 ratio to receive either the E-ZES or another currently available DES. Randomization was stratified by participating center and 4 clinical or lesion characteristics (Fig. 1): diabetes mellitus, acute coronary syndrome, treatment of a short lesion (stent length ≤24 mm); and treatment of a long lesion (stent length ≥28 mm). Patients with diabetes mellitus or acute coronary syndrome were randomized to either the E-ZES or the Resolute zotarolimus-eluting stent (Medtronic); patients with short lesions to the E-ZES or the Cypher Select sirolimus-eluting stent (Cordis, Miami, Florida); and those with long lesions to the E-ZES or the Xience V

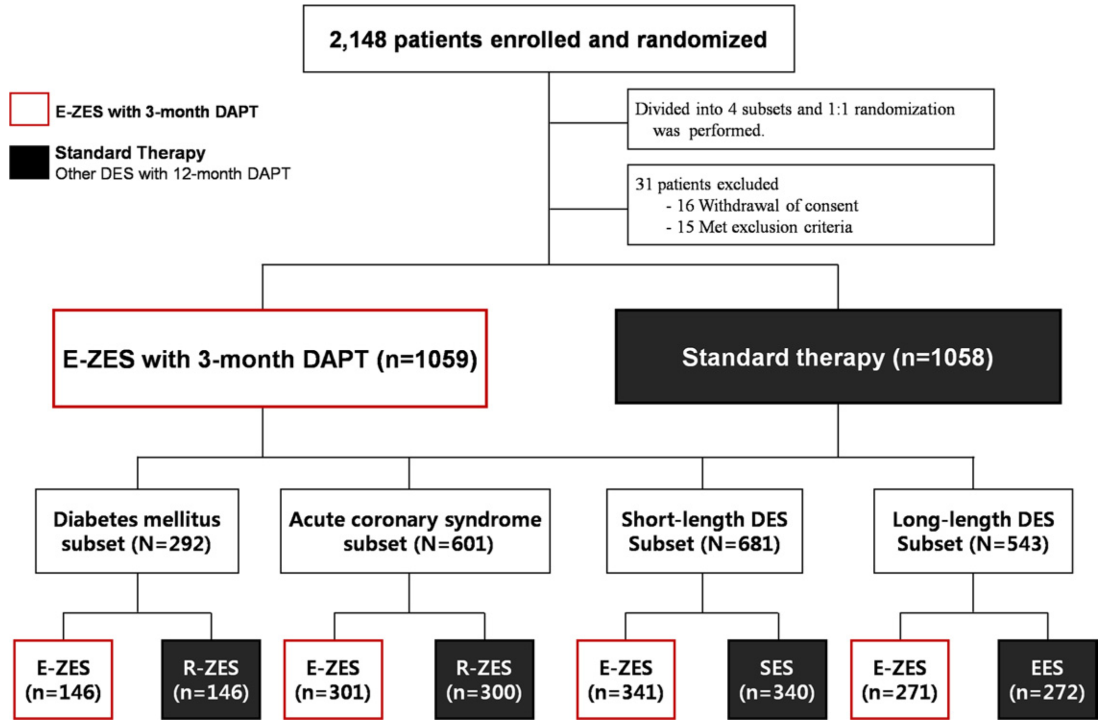


**Figure 1** **Diagram for Study Design and the Detailed Enrollment of Patients**

DAPT = dual antiplatelet therapy; DES = drug-eluting stent; EES = everolimus-eluting stent(s); E-ZES = Endeavor zotarolimus-eluting stent(s); R-ZES = Resolute zotarolimus-eluting stent(s); SES = sirolimus-eluting stent(s).

**Table 1 Baseline Clinical, Angiographic, and Procedural Characteristics**

| Variables | E-ZES+3-Month DAPT | Standard Therapy | p Value |
|---|---|---|---|
| Duration of DAPT, days | 93 ± 28 | 364 ± 31 | <0.001 |
| Clinical characteristics | | | |
| n | 1,059 | 1,058 | |
| Age, yrs | 62.4 ± 9.4 | 62.4 ± 9.8 | 0.94 |
| Male | 682 (64.4) | 665 (62.9) | 0.47 |
| Body mass index, kg/m$^2$ | 25.0 ± 3.2 | 24.9 ± 3.1 | 0.50 |
| Hypertension | 660 (62.3) | 650 (61.4) | 0.69 |
| Diabetes mellitus | 316 (29.8) | 305 (28.8) | 0.63 |
| Dyslipidemia | 611 (57.7) | 634 (59.9) | 0.31 |
| Current smoker | 267 (25.2) | 241 (22.8) | 0.20 |
| Congestive heart failure | 120 (11.3) | 125 (11.8) | 0.74 |
| Ejection fraction, % | 64.2 ± 9.4 | 63.9 ± 9.4 | 0.45 |
| Prior myocardial infarction | 19 (1.8) | 17 (1.6) | 0.87 |
| Prior percutaneous coronary intervention | 37 (3.5) | 32 (3.0) | 0.63 |
| Prior coronary bypass surgery | 2 (0.2) | 6 (0.6) | 0.18 |
| Clinical presentation | | | 0.66 |
| Stable angina | 471 (44.5) | 490 (46.3) | |
| Unstable angina | 432 (40.8) | 422 (39.9) | |
| Acute myocardial infarction | 156 (14.7) | 146 (13.8) | |
| No. of diseased vessels | | | 0.99 |
| 1 | 603 (56.9) | 604 (57.1) | |
| 2 | 292 (27.6) | 292 (27.6) | |
| 3 | 164 (15.5) | 162 (15.3) | |
| Medications at discharge | | | |
| Statins | 923 (87.2) | 914 (86.4) | 0.61 |
| Beta-blockers | 712 (67.2) | 730 (69.0) | 0.40 |
| Angiotensin-converting enzyme inhibitors | 331 (31.3) | 349 (33.0) | 0.40 |
| Angiotensin receptor blockers | 323 (30.5) | 301 (28.4) | 0.32 |
| Calcium channel blocker | 389 (36.7) | 389 (36.8) | 1.00 |
| Angiographic and procedural characteristics | | | |
| No. of lesions | 1,341 | 1,346 | |
| Treated vessel | | | 0.54 |
| Left anterior descending artery | 707 (52.7) | 722 (53.6) | |
| Left circumflex artery | 281 (21.0) | 259 (19.2) | |
| Right coronary artery | 353 (26.3) | 365 (27.1) | |
| ACC/AHA class B2/C | 910 (67.9) | 932 (69.2) | 0.46 |
| Lesion length, mm | 19.6 ± 10.1 | 20.1 ± 10.8 | 0.21 |
| Type of drug-eluting stent | | | |
| E-ZES | 1,341 (100.0) | — | |
| Cypher sirolimus-eluting stents | — | 383 (28.5) | |
| Xience everolimus-eluting stents | — | 404 (30.0) | |
| Resolute zotarolimus-eluting stents | — | 559 (41.5) | |
| Multivessel intervention/patients | 233 (22.0) | 248 (23.4) | 0.44 |
| Number of lesions per patient | 1.27 ± 0.53 | 1.27 ± 0.68 | 0.88 |
| Stent diameter, mm | 3.18 ± 0.42 | 3.17 ± 0.83 | 0.63 |
| Stent length per lesion, mm | 22.7 ± 10.1 | 22.9 ± 10.7 | 0.35 |
| Adjuvant post-dilation | 539 (40.2) | 540 (40.1) | 0.97 |
| Maximum stent pressure, atm | 16.2 ± 3.7 | 16.5 ± 3.6 | 0.35 |
| Use of glycoprotein IIb/IIIa inhibitors/patient | 20 (1.9) | 21 (2.0) | 0.89 |
| Procedure success | 1,339 (99.9) | 1,345 (99.9) | 0.63 |

*Continued on next page*

everolimus-eluting stents (Abbott Vascular, Santa Clara, California).

After stent implantation, 100-mg daily aspirin was prescribed indefinitely, and the duration of clopidogrel 75-mg daily was given depending on the randomization scheme (Fig. 1). Details of study procedures and quantitative coronary angiographic analyses are provided in the Online Appendix.

Post-procedure clinical assessment was performed in-hospital, and after 1, 3, 6, and 12 months, either by clinic visit or by telephone interview. The primary endpoint was a

**Table 1 Continued**

| Variables | E-ZES+3-Month DAPT | Standard Therapy | p Value |
|---|---|---|---|
| Quantitative angiographic analysis | | | |
| Pre-intervention | | | |
| Reference vessel diameter, mm | 3.0 ± 0.5 | 3.0 ± 0.5 | 0.13 |
| Minimum luminal diameter, mm | 1.1 ± 0.5 | 1.0 ± 0.5 | 0.23 |
| Percent diameter stenosis, % | 65.0 ± 14.1 | 65.5 ± 13.8 | 0.36 |
| Post-intervention | | | |
| Minimum luminal diameter, mm | | | |
| In-stent | 2.7 ± 0.4 | 2.7 ± 0.4 | 0.28 |
| In-segment | 2.2 ± 0.5 | 2.1 ± 0.5 | 0.58 |
| Percent diameter stenosis, % | | | |
| In-stent | 11.2 ± 7.8 | 11.1 ± 8.1 | 0.65 |
| In-segment | 30.7 ± 11.7 | 30.7 ± 11.7 | 0.83 |

Values are mean ± SD, n, or n (%).
ACC = American College of Cardiology; AHA = American Heart Association; DAPT = dual antiplatelet therapy; E-ZES = Endeavor zotarolimus-eluting stent(s).

composite of death from cardiovascular cause, myocardial infarction, stent thrombosis, ischemia-driven target-vessel revascularization, or bleeding at 1-year post-procedure. Clinical events are defined according to the Academic Research Consortium (9). Detailed definitions of study endpoints, clinical diseases, and procedural findings are provided in the Online Appendix. All clinical events were independently monitored and assessed by a clinical event committee, comprising members masked as to the assigned therapy groups.

The primary analysis was a noninferiority comparison between the 2 groups with respect to the occurrence of the primary endpoint. On the basis of the previous studies, we assumed the overall incidence of the primary endpoint after E-ZES+3-month DAPT, and after the standard therapy, would be 10% and 11%, respectively (4–6,10–12). We hypothesized that the clinical outcome of E-ZES+3-month DAPT would be noninferior to the other group, with a noninferiority margin of 4% for the absolute difference in risk at 12 months. Assuming a 10% dropout rate, this required an estimated sample size of 2,120 patients (1,060 for each group) to achieve 80% power for the noninferiority test and a 1-sided type I error of 5%. The detailed methods of statistical analysis are provided in the Online Appendix.

## Results

Between April 2009 and December 2010, we enrolled and randomized 2,148 patients, of which 2,117 patients (E-ZES+3-month DAPT = 1,059; standard therapy = 1,058) comprised the analysis population. The study design and the detailed enrollment of patients are provided in Figure 1. The baseline characteristics were similar between the 2 groups (Table 1). Clinical follow-up at 1 year was completed for 2,086 of 2,117 patients (98.5%): 1,044 of 1,059 patients (98.6%) in E-ZES+3-month DAPT group, and 1,042 of 1,058 patients (98.5%) in standard therapy group (p = 0.99). Clinical outcomes through 1-year follow-up are listed in Table 2. At 1 year, the E-ZES+3-month DAPT group was noninferior to the standard therapy group for the primary endpoint (cumulative events: 40 [4.7%] vs. 41 [4.7%]; difference: 0.0%, 95% confidence interval [CI]: −2.5 to 2.5; p = 0.84; p < 0.001 for noninferiority) (Fig. 2A). The cumulative events rates of the composite of any death, myocardial infarction, or stent thrombosis were 0.8% and 1.3%, respectively (difference: −0.5%; 95% CI: −1.5 to 0.5; p = 0.48, Fig. 2B). The occurrence of stent thrombosis was similar between the 2 groups (0.2% vs. 0.3%; difference: −0.1%; 95% CI: −0.5 to 0.3; p = 0.65). From 3 months through 12 months following the index procedure, there were 3 stent thrombosis events in the standard therapy group, and none in the E-ZES+3-month DAPT group despite the cessation of clopidogrel. The rates of target-vessel revascularization were 3.9% for the E-ZES+3-month DAPT group and 3.7% for the standard therapy group. The subgroup analysis of the primary endpoint and other events at 1 year is shown in Figure 3 and Table 2.

Interruption of the DAPT regimen occurred in 62 (5.9%) of 1,059 patients who were allocated to E-ZES+3-month DAPT (mean duration of DAPT: 196 ± 63 days). Reasons for interruption of the DAPT regimen were as follows: physicians' mistake or failure of monitoring (n = 26), physicians' discretion (n = 22), patients' disagreement (n = 13), and repeat revascularization (n = 1). After censoring patients who had an interruption of DAPT duration in the E-ZES+3-month DAPT group, there were no significant differences in 1-year clinical outcomes between the 2 groups (Tables 3 and 4).

## Discussion

This randomized study demonstrated that E-ZES+3-month DAPT is safe and noninferior to the standard therapy for the primary composite endpoint.

Regardless of DES types, current recommendations call for a minimum of 12 months of DAPT after DES implantation for the prevention of late stent thrombosis (3). However, prolonged DAPT has been associated with

**Table 2 Clinical Outcomes Through 1 Year**

| Variables | E-ZES+3-Month DAPT (n = 1,059) | Standard Therapy (n = 1,058) | Difference (95% CI) | p Value |
|---|---|---|---|---|
| Composite events | | | | |
| Primary endpoint | 40 (4.7) | 41 (4.7) | 0.0% (−2.5 to 2.5) | 0.84 |
| Death from any cause, myocardial infarction, or stent thrombosis | 8 (0.8) | 11 (1.3) | −0.5% (−1.5 to 0.5) | 0.48 |
| Death from cardiovascular cause or myocardial infarction | 4 (0.4) | 7 (0.7) | −0.3% (−1.0 to 0.4) | 0.36 |
| Each component | | | | |
| Death | | | | |
| From any cause | 5 (0.5) | 8 (1.0) | −0.5% (−1.4 to 0.4) | 0.39 |
| From cardiovascular cause | 2 (0.2) | 4 (0.4) | −0.2% (−0.6 to 0.3) | 0.41 |
| Myocardial infarction | 2 (0.2) | 4 (0.4) | −0.2% (−0.7 to 0.3) | 0.41 |
| Target vessel revascularization | 31 (3.9) | 27 (3.7) | 0.2% (−2.3 to 2.6) | 0.70 |
| Non–target vessel revascularization | 15 (1.5) | 11 (1.5) | 0.0% (−1.3 to 1.4) | 0.52 |
| Stent thrombosis, definite or probable | 2 (0.2) | 3 (0.3) | −0.1% (−0.5 to 0.3) | 0.65 |
| <1 month | 2 | 0 | | |
| 1–3 months | 0 | 0 | | |
| 3–12 months | 0 | 3 | | |
| Bleeding | | | | |
| Major or minor | 5 (0.5) | 10 (1.0) | −0.5% (−1.2 to 0.2) | 0.20 |
| Major | 2 (0.2) | 6 (0.6) | −0.4% (−0.9 to 0.1) | 0.16 |
| Cerebrovascular accidents | 6 (0.6) | 6 (0.7) | 0.1% (−0.1 to 1.0) | 0.96 |
| Subgroup analysis | | | | |
| Diabetes mellitus subset | 146 | 146 | — | — |
| Primary endpoint | 4 (2.8) | 5 (3.4) | −0.6% (−4.6 to 3.3) | 0.74 |
| Death from cardiovascular cause | 1 (0.7) | 1 (0.7) | 0.0% (−1.9 to 1.9) | 1.00 |
| Myocardial infarction | 0 (0.0) | 1 (0.7) | | 0.32 |
| Target vessel revascularization | 3 (2.1) | 2 (1.4) | 0.7% (−2.3 to 3.7) | 0.65 |
| Stent thrombosis, definite or probable | 0 (0.0) | 1 (0.7) | | 0.32 |
| Bleeding, major or minor | 0 (0.0) | 2 (1.4) | | 0.16 |
| Acute coronary syndrome subset, n | 301 | 300 | — | — |
| Primary endpoint | 12 (6.5) | 6 (2.0) | 4.4% (−1.4 to 10.2) | 0.16 |
| Death from cardiovascular cause | 1 (0.3) | 0 (0.0) | | 0.32 |
| Myocardial infarction | 0 (0.0) | 0 (0.0) | | 1.00 |
| Target vessel revascularization | 9 (5.4) | 2 (0.7) | 4.7% (−0.8 to 10.1) | 0.04 |
| Stent thrombosis, definite or probable | 1 (0.3) | 0 (0.0) | −0.9% (−5.1 to 3.4) | 0.32 |
| Bleeding, major or minor | 2 (0.7) | 4 (1.3) | −0.7% (−2.3 to 0.9) | 0.41 |
| Short-lesion drug-eluting stent subset, n | 341 | 340 | — | — |
| Primary endpoint | 9 (2.7) | 8 (4.1) | −1.5% (−5.3 to 2.4) | 0.86 |
| Death from cardiovascular cause | 0 (0.0) | 2 (0.6) | | 0.16 |
| Myocardial infarction | 1 (0.3) | 2 (0.6) | −0.3% (−1.3 to 0.7) | 0.60 |
| Target–vessel revascularization | 6 (1.8) | 6 (3.6) | −1.8% (−5.5 to 1.9) | 0.91 |
| Stent thrombosis, definite or probable | 0 (0.0) | 1 (0.3) | | 0.32 |
| Bleeding, major or minor | 2 (0.6) | 0 (0.0) | | 0.16 |
| Long-lesion drug-eluting stent subset, n | 271 | 272 | — | — |
| Primary endpoint | 15 (7.2) | 22 (8.4) | −1.2% (−6.6 to 4.3) | 0.22 |
| Death from cardiovascular cause | 0 (0.0) | 1 (0.4) | | 0.32 |
| Myocardial infarction | 1 (0.4) | 1 (0.4) | 0.0% (−1.0 to 1.0) | 0.99 |
| Target vessel revascularization | 13 (6.3) | 17 (7.8) | −1.4% (−7.2 to 4.3) | 0.40 |
| Stent thrombosis, definite or probable | 1 (0.4) | 1 (0.4) | 0.0% (−1.0 to 1.0) | 0.99 |
| Bleeding, major or minor | 1 (0.4) | 4 (1.5) | −1.1% (−2.7 to 0.5) | 0.18 |

Values are the number of events and the cumulative event rate (%). *p values were calculated with the use of the log-rank test. In case of no clinical event in either group, the confidence interval (CI) of the differences of event rates could not be calculated.
Abbreviations as in Table 1.

higher severe bleeding rates compared with treatment with aspirin alone; reported incidence of major and minor bleeding were 1.8% to 3.7% and 1.7% to 5.1%, respectively (13,14). In addition, nuisance bleeding is common in patients on prolonged DAPT post-DES implantation (28.9% of 2,948 patients) (14). The higher incidence of

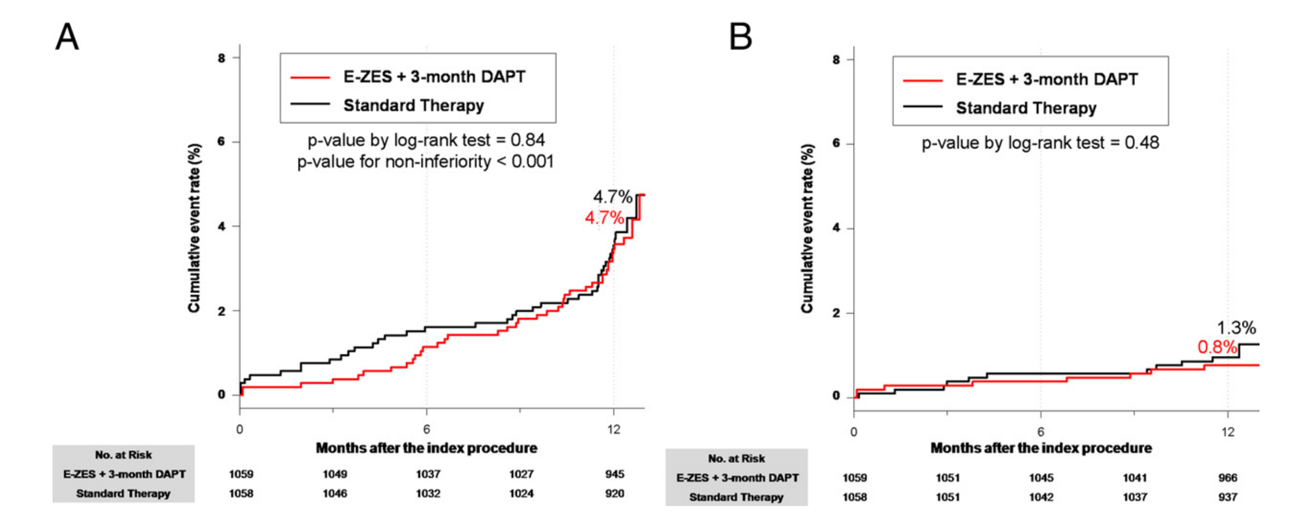


**Figure 2** **Cumulative Event Rates Using the Kaplan-Meier Method**

A primary endpoint **(A)**, and a composite of death from any cause, myocardial infarction, or stent thrombosis **(B)**. Abbreviations as in Figure 1.

bleeding episodes can impact patients' compliance and result in premature discontinuation of DAPT.

A previous randomized study reported that the use of DAPT for a period longer than 12 months in patients who had received DESs was not significantly more effective than aspirin monotherapy in reducing the rate of myocardial infarction or death from cardiac causes (15). In addition, recent randomized trials showed no clinical benefits of prolonged DAPT compared with 6-month DAPT after DES implantation (16,17).

| Subgroup | E-ZES + 3-month DAPT | Standard Therapy | Difference | 95% CI | p-value | p-for-interaction |
|---|---|---|---|---|---|---|
| **Age** | | | | | | **0.599** |
| < 65 (n=1153) | 20/582 (4.4%) | 18/571 (4.5%) | -0.2% | (-3.8%~3.3%) | 0.805 | |
| ≥ 65 (n=964) | 20/477 (5.1%) | 23/487 (4.8%) | 0.4% | (-2.8%~3.6%) | 0.636 | |
| **Gender** | | | | | | **0.424** |
| Male (n=1347) | 29/682 (5.8%) | 26/665 (5.1%) | 0.6% | (-3.0%~4.2%) | 0.786 | |
| Female (n=770) | 11/377 (3.0%) | 15/393 (3.8%) | -0.9% | (-3.4%~1.7%) | 0.483 | |
| **Diabetes mellitus** | | | | | | **0.416** |
| Yes (n=621) | 11/316 (3.8%) | 14/305 (4.7%) | -0.9% | (-4.1%~2.4%) | 0.426 | |
| No (n=1496) | 29/743 (5.1%) | 27/753 (4.6%) | 0.5% | (-2.7%~3.6%) | 0.769 | |
| **Congestive heart failure** | | | | | | **0.840** |
| Yes (n=245) | 4/120 (3.4%) | 5/125 (4.0%) | -0.6% | (-5.4%~4.1%) | 0.783 | |
| No (n=1872) | 36/939 (4.8%) | 36/933 (4.8%) | 0.0% | (-2.7%~2.8%) | 0.897 | |
| **Multivessel disease** | | | | | | **0.902** |
| Yes (n=910) | 18/456 (4.2%) | 19/454 (4.3%) | -0.1% | (-2.8%~2.6%) | 0.829 | |
| No (n=1207) | 22/603 (5.3%) | 22/604 (5.1%) | 0.1% | (-3.9%~4.2%) | 0.934 | |
| **Reference diameter** | | | | | | **0.105** |
| ≤ 3.0mm (n=669) | 8/322 (2.5%) | 16/347 (6.6%) | -4.1% | (-8.6%~0.4%) | 0.166 | |
| > 3.0mm (n=1448) | 32/737 (5.7%) | 25/711 (3.5%) | 2.2% | (-0.7%~5.0%) | 0.370 | |
| **Lesion length** | | | | | | **0.203** |
| < 20mm (n=1301) | 20/653 (3.9%) | 15/648 (3.4%) | 0.5% | (-2.7%~3.8%) | 0.330 | |
| ≥ 20mm (n=816) | 20/406 (6.0%) | 26/410 (6.4%) | -0.4% | (-4.3%~3.5%) | 0.400 | |
| **Subsets** | | | | | | **0.344** |
| Acute coronary syndrome (n=601) | 12/301 (6.4%) | 6/300 (2.0%) | 4.4% | (-1.4%~10.2%) | 0.158 | |
| Diabetes mellitus (n=292) | 4/146 (2.8%) | 5/146 (3.4%) | -0.6% | (-4.6%~3.3%) | 0.735 | |
| Long (n=543) | 15/271 (7.2%) | 22/272 (8.4%) | -1.2% | (-6.6%~4.3%) | 0.219 | |
| Short (n=681) | 9/341 (2.7%) | 8/340 (4.1%) | -1.5% | (-5.3%~2.4%) | 0.855 | |
| **Overall (n=2117)** | **40/1059 (4.7%)** | **41/1058 (4.7%)** | **0.0%** | **(-2.5%~2.5%)** | **0.843** | |

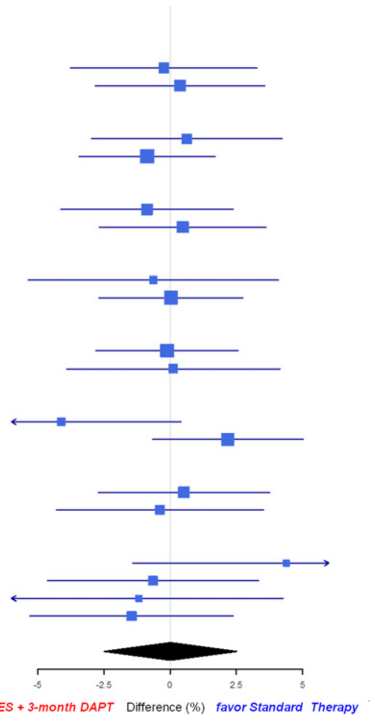


**Figure 3** **Subgroup Analysis of the Primary Endpoint at 1 Year**

CI = confidence interval; other abbreviations as in Figure 1.

**Table 3 Baseline Clinical and Angiographic Characteristics of Both Groups on a Per Protocol Analysis**

| Variables | E-ZES+3-Month DAPT | Standard Therapy | p Value |
|---|---|---|---|
| Clinical characteristics | | | |
| n | 997 | 1,058 | |
| Age, yrs | 62.4 ± 9.4 | 62.4 ± 9.8 | 0.93 |
| Male | 647 (64.9) | 665 (62.9) | 0.36 |
| Body mass index, kg/m$^2$ | 25.0 ± 3.2 | 24.9 ± 3.1 | 0.47 |
| Hypertension | 624 (62.6) | 650 (61.4) | 0.62 |
| Diabetes mellitus | 300 (30.1) | 305 (28.8) | 0.53 |
| Dyslipidemia | 580 (58.2) | 634 (59.9) | 0.45 |
| Current smoker | 249 (25.0) | 241 (22.8) | 0.23 |
| Congestive heart failure | 100 (10.0) | 125 (11.8) | 0.20 |
| Ejection fraction, % | 64.3 ± 9.2 | 63.9 ± 9.4 | 0.33 |
| Prior myocardial infarction | 18 (1.8) | 17 (1.6) | 0.74 |
| Prior percutaneous coronary intervention | 37 (3.7) | 32 (3.0) | 0.39 |
| Prior coronary bypass surgery | 2 (0.2) | 6 (0.6) | 0.29 |
| Clinical presentation | | | 0.84 |
| Stable angina | 453 (45.4) | 490 (46.3) | |
| Unstable angina | 398 (39.9) | 422 (39.9) | |
| Acute myocardial infarction | 146 (14.6) | 146 (13.8) | |
| No. of diseased vessels | | | 0.88 |
| 1 | 576 (57.8) | 604 (57.1) | |
| 2 | 271 (27.2) | 292 (27.6) | |
| 3 | 150 (15.0) | 162 (15.3) | |
| Medications at discharge | | | |
| Statins | 874 (87.7) | 914 (86.4) | 0.39 |
| Beta-blockers | 670 (67.2) | 730 (69.0) | 0.39 |
| Angiotensin-converting enzyme inhibitors | 311 (31.2) | 349 (33.0) | 0.40 |
| Angiotensin receptor blockers | 308 (30.9) | 301 (28.4) | 0.23 |
| Calcium channel blocker | 370 (37.1) | 389 (36.8) | 0.89 |
| Angiographic characteristics | | | |
| No. of lesions | 1,261 | 1,346 | |
| Treated vessel | | | 0.43 |
| Left anterior descending artery | 664 (52.7) | 722 (53.6) | |
| Left circumflex artery | 268 (21.3) | 259 (19.2) | |
| Right coronary artery | 329 (26.1) | 365 (27.1) | |
| ACC/AHA lesion class B2 or C | 852 (67.6) | 932 (69.2) | 0.38 |
| Lesion length, mm | 19.6 ± 10.1 | 20.1 ± 10.8 | 0.19 |
| Type of drug-eluting stent | | | |
| E-ZES | 1,261 (100.0) | — | |
| Cypher sirolimus-eluting stents | — | 383 (28.5) | |
| Xience everolimus-eluting stents | — | 404 (30.0) | |
| Resolute zotarolimus-eluting stents | — | 559 (41.5) | |
| Multivessel intervention/patients | 217 (21.8) | 248 (23.4) | 0.37 |
| Total no. of lesions per patient | 1.27 ± 0.54 | 1.27 ± 0.68 | 0.78 |
| Stent diameter, mm | 3.18 ± 0.42 | 3.17 ± 0.83 | 0.73 |
| Stent length per lesion, mm | 22.7 ± 10.1 | 22.9 ± 10.7 | 0.71 |
| Adjuvant post-dilation | 504 (40.0) | 540 (40.1) | 0.95 |
| Maximum stent pressure, atm | 16.5 ± 3.7 | 16.5 ± 3.6 | 0.74 |
| Use of glycoprotein IIb/IIIa inhibitors/patient | 19 (1.9) | 21 (2.0) | 0.63 |
| Procedure success | 1,259 (99.8) | 1,345 (99.9) | 0.61 |

*Continued on next page*

Therefore, balanced DES that can offer both safety and efficacy are desirable, especially for those who may need to stop DAPT early after DES implantation (5,6). The E-ZES comprises a cobalt alloy, thin-strut stent with a biocompatible phosphorylcholine polymer (4–6). A recent study reported that among 2,032 patients treated with E-ZES in 5 trials, Academic Research Consortium–defined definite or probable stent thrombosis rates through 3 years

**Table 3 Continued**

| Variables | E-ZES+3-Month DAPT | Standard Therapy | p Value |
|---|---|---|---|
| Quantitative angiographic analysis | | | |
| Pre-intervention | | | |
| Reference vessel diameter, mm | 3.0 ± 0.5 | 3.0 ± 0.5 | 0.18 |
| Minimum luminal diameter, mm | 1.1 ± 0.5 | 1.0 ± 0.5 | 0.39 |
| Percent diameter stenosis, % | 65.1 ± 14.1 | 65.5 ± 13.8 | 0.52 |
| Post-intervention | | | |
| Minimum luminal diameter, mm | | | |
| In-stent | 2.7 ± 0.4 | 2.7 ± 0.4 | 0.24 |
| In-segment | 2.2 ± 0.5 | 2.1 ± 0.5 | 0.66 |
| Percent diameter stenosis, % | | | |
| In-stent | 11.1 ± 7.8 | 11.1 ± 8.1 | 0.83 |
| In-segment | 29.8 ± 11.8 | 30.7 ± 11.7 | 0.76 |

Values are n, mean ± SD, or n (%). Analysis was performed after exclusion of the patients interrupting the 3-month DAPT criteria.
Abbreviations as in Table 1.

did not significantly differ between the 6-month and ≥12-month DAPT groups (0.3% vs. 0%, respectively) (18). These findings might be explained by better neointimal coverage in the early post-implant period compared with other DES (7).

**Study limitations.** First, 1 year of clinical follow-up may not be sufficient to assess the late outcomes, especially the occurrence of very late stent thrombosis. Second, because the patients with very high risks were not included, the generalized application of these results to the entire population demands careful attention. A careful assessment of the balance between the risk of stent thrombosis and the likelihood of bleeding events at an individual patient level is required (19). Third, the study design was not ideal: the comparator group in our trial was not treated with a single DES type; in addition, there was no 3-month versus 12-month DAPT, either within E-ZES or within other DES patients. However, because the hypothesis of protection by E-ZES was the main objective of this trial, and the 1:1 matched randomization between E-ZES and the comparative DES was performed, interpretation of the final results of the E-ZES+3-month DAPT group should be viewed appropriate. Treatment strategies (combination of DES+ duration of DAPT), neither DES types alone, nor DAPT duration alone were evaluated in this study. Finally, although the sample size of this study was calculated not to be underpowered on the basis of the event rates in

**Table 4 Clinical Outcomes of Both Groups on a Per-Protocol Analysis**

| Characteristics | E-ZES+3-Month DAPT (n = 997) | Standard Therapy (n = 1,058) | Difference (95% CI) | p Value |
|---|---|---|---|---|
| Composite events | | | | |
| Primary endpoint | 36 (4.6) | 41 (4.7) | −0.1% (−2.7 to 2.4) | 0.69 |
| Death from any cause, myocardial infarction, or stent thrombosis | 6 (0.6) | 11 (1.3) | −0.7% (−1.6 to 0.3) | 0.27 |
| Death from cardiovascular cause or myocardial infarction | 4 (0.4) | 7 (0.7) | −0.3% (−0.9 to 0.4) | 0.42 |
| Each component | | | | |
| Death | | | | |
| From any cause | 3 (0.3) | 8 (1.0) | −0.7% (−1.5 to 0.2) | 0.15 |
| From cardiovascular cause | 2 (0.2) | 4 (0.4) | −0.2% (−0.6 to 0.3) | 0.46 |
| Myocardial infarction | 2 (0.2) | 4 (0.4) | −0.2% (−0.7 to 0.3) | 0.46 |
| Target vessel revascularization | 27 (3.7) | 27 (3.7) | 0.0% (−2.5 to 2.4) | 0.94 |
| Non–target vessel revascularization | 14 (1.5) | 11 (1.5) | 0.0% (−1.4 to 1.4) | 0.55 |
| Stent thrombosis, definite or probable | 2 (0.2) | 3 (0.3) | −0.1% (−0.5 to 0.3) | 0.70 |
| <1 months | 2 | 0 | | |
| 1–3 months | 0 | 0 | | |
| 3–12 months | 0 | 3 | | |
| Bleeding | | | | |
| Major or minor | 5 (0.5) | 10 (1.0) | −0.5% (−1.2 to 0.3) | 0.24 |
| Major | 2 (0.2) | 6 (0.6) | −0.4% (−0.9 to 0.2) | 0.18 |
| Cerebrovascular accidents | 5 (0.5) | 6 (0.7) | −0.2% (−0.9 to 0.6) | 0.80 |

Values are the number of events and the cumulative event rate (%). Analysis was performed after exclusion of the patients with interrupting 3-month DAPT. *p values were calculated with the use of the log-rank test.
Abbreviations as in Tables 1 and 2.

prior studies (4–6,10–12), the findings of this study could be underpowered as a result of a relatively lower event rate than expected. The authors cannot know what among many factors was responsible for the differences from anticipated event rates.

## Conclusions

E-ZES+3-month DAPT could be safe and beneficial for the selected patients with coronary artery disease who may need to stop DAPT early after DES implantation.

**Reprint requests and correspondence:** Dr. Myeong-Ki Hong, Division of Cardiology, Severance Cardiovascular Hospital, Yonsei University College of Medicine, 250 Seongsanno, Seodaemun-gu, Seoul 120-752, Republic of Korea. E-mail: mkhong61@yuhs.ac.

## REFERENCES

1. Iakovou I, Schmidt T, Bonizzoni E, et al. Incidence, predictors, and outcome of thrombosis after successful implantation of drug-eluting stents. JAMA 2005;293:2126–30.
2. Pfisterer M, Brunner-La Rocca HP, Buser PT, et al. Late clinical events after clopidogrel discontinuation may limit the benefit of drug-eluting stents: an observational study of drug-eluting versus bare-metal stents. J Am Coll Cardiol 2006;48:2584–91.
3. Levine GN, Bates ER, Blankenship JC, et al. 2011 ACCF/AHA/SCAI guideline for percutaneous coronary intervention: executive summary: a report of the American College of Cardiology Foundation/American Heart Association Task Force on Practice Guidelines and the Society for Cardiovascular Angiography and Interventions. J Am Coll Cardiol 2011;58:2550–83.
4. Fajadet J, Wijns W, Laarman GJ, et al. Randomized, double-blind, multicenter study of the Endeavor zotarolimus-eluting phosphorylcholine-encapsulated stent for treatment of native coronary artery lesions: clinical and angiographic results of the ENDEAVOR II trial. Circulation 2006;114:798–806.
5. Meredith IT, Ormiston J, Whitbourn R, et al. Four-year clinical follow-up after implantation of the endeavor zotarolimus-eluting stent: ENDEAVOR I, the first-in-human study. Am J Cardiol 2007;100:S56–61.
6. Leon MB, Mauri L, Popma JJ, et al. A randomized comparison of the ENDEAVOR zotarolimus-eluting stent versus the TAXUS paclitaxel-eluting stent in de novo native coronary lesions: 12-month outcomes from the ENDEAVOR IV trial. J Am Coll Cardiol 2010;55:543–54.
7. Kim JS, Jang IK, Fan C, et al. Evaluation in 3 months duration of neointimal coverage after zotarolimus-eluting stent implantation by optical coherence tomography: the ENDEAVOR OCT trial. J Am Coll Cardiol Intv 2009;2:1240–7.
8. Hahn JY, Song YB, Choi JH, et al. Three-month dual antiplatelet therapy after implantation of zotarolimus-eluting stents: the DATE (Duration of dual Antiplatelet Therapy after implantation of Endeavor stent) registry. Circ J 2010;74:2314–21.
9. Cutlip DE, Windecker S, Mehran R, et al. Clinical end points in coronary stent trials: a case for standardized definitions. Circulation 2007;115:2344–51.
10. Moses JW, Leon MB, Popma JJ, et al. Sirolimus-eluting stents versus standard stents in patients with stenosis in a native coronary artery. N Engl J Med 2003;349:1315–23.
11. Stone GW, Midei M, Newman W, et al. Randomized comparison of everolimus-eluting and paclitaxel-eluting stents: two-year clinical follow-up from the clinical evaluation of the Xience V everolimus eluting coronary stent system in the treatment of patients with de novo native coronary artery lesions (SPIRIT) III trial. Circulation 2009;119:680–6.
12. Meredith IT, Worthley S, Whitbourn R, et al. Clinical and angiographic results with the next-generation resolute stent system: a prospective, multicenter, first-in-human trial. J Am Coll Cardiol Intv 2009;2:977–85.
13. Yusuf S, Zhao F, Mehta SR, Chrolavicius S, Tognoni G, Fox KK. Effects of clopidogrel in addition to aspirin in patients with acute coronary syndromes without ST-segment elevation. N Engl J Med 2001;345:494–502.
14. Ben-Dor I, Torguson R, Scheinowitz M, et al. Incidence, correlates, and clinical impact of nuisance bleeding after antiplatelet therapy for patients with drug-eluting stents. Am Heart J 2010;159:871–5.
15. Park SJ, Park D-W, Kim Y-H, et al. Duration of dual antiplatelet therapy after implantation of drug-eluting stents. N Engl J Med 2010;362:1374–82.
16. Valgimigli M, Campo G, Monti M, et al. Short- versus long-term duration of dual antiplatelet therapy after coronary stenting: a randomized multicentre trial. Circulation 2012;125:2015–26.
17. Gwon HC, Hahn JY, Park KW, et al. Six-month versus 12-month dual antiplatelet therapy after implantation of drug-eluting stents: the efficacy of Xience/Promus versus Cypher to reduce late loss after stenting (EXCELLENT) randomized, multicenter study. Circulation 2012;125:505–13.
18. Kandzari DE, Barker CS, Leon MB, et al. Dual antiplatelet therapy duration and clinical outcomes following treatment with zotarolimus-eluting stents. J Am Coll Cardiol Intv 2011;4:1119–28.
19. Kastrati A, Byrne RA, Schulz S. Will we ever know the optimal duration of dual antiplatelet therapy after drug-eluting stent implantation? J Am Coll Cardiol Intv 2011;4:1129–32.

**Key Words:** antiplatelet therapy ■ coronary artery disease ■ drug-eluting stents.

## APPENDIX

**For a supplementary introduction, methods, discussion, and references, as well as expanded information on the trial investigators, please see the online version of this paper.**
