# Supplementary material for: Optimal duration of DAPT after second-generation drug-eluting stent in acute coronary syndrome
Source: PLoS One. 2018 Nov 26;13(11):e0207386. doi: 10.1371/journal.pone.0207386 (PMC6261023; doi:10.1371/journal.pone.0207386)
Supplement: S2 File — (PDF) [file pone.0207386.s005.pdf]

Six-Month Versus 12-Month Dual Antiplatelet Therapy After Implantation of Drug-Eluting Stents

The Efficacy of Xience/Promus Versus Cypher to Reduce Late Loss After Stenting (EXCELLENT) Randomized, Multicenter Study

Hyeon-Cheol Gwon, MD*; Joo-Yong Hahn, MD*; Kyung Woo Park, MD; Young Bin Song, MD; In-Ho Chae, MD; Do-Sun Lim, MD; Kyoo-Rok Han, MD; Jin-Ho Choi, MD; Seung-Hyuk Choi, MD; Hyun-Jae Kang, MD; Bon-Kwon Koo, MD; Taehoon Ahn, MD; Jung-Han Yoon, MD; Myung-Ho Jeong, MD; Taek-Jong Hong, MD; Woo-Young Chung, MD; Young-Jin Choi, MD; Seung-Ho Hur, MD; Hyuck-Moon Kwon, MD; Dong-Woon Jeon, MD; Byung-Ok Kim, MD; Si-Hoon Park, MD; Nam-Ho Lee, MD; Hui-Kyung Jeon, MD; Yangsoo Jang, MD; Hyo-Soo Kim, MD

Background—The optimal duration of dual antiplatelet therapy (DAPT) after implantation of drug-eluting coronary stents remains undetermined. We aimed to test whether 6-month DAPT would be noninferior to 12-month DAPT after implantation of drug-eluting stents.

Methods and Results—We randomly assigned 1443 patients undergoing implantation of drug-eluting stents to receive 6- or 12-month DAPT (in a 1:1 ratio). The primary end point was a target vessel failure, defined as the composite of cardiac death, myocardial infarction, or ischemia-driven target vessel revascularization at 12 months. Rates of target vessel failure at 12 months were 4.8% in the 6-month DAPT group and 4.3% in the 12-month DAPT group (the upper limit of 1-sided 95% confidence interval, 2.4%; $P=0.001$ for noninferiority with a predefined noninferiority margin of 4.0%). Although stent thrombosis tended to occur more frequently in the 6-month DAPT group than in the 12-month group (0.9% versus 0.1%; hazard ratio, 6.02; 95% confidence interval, 0.72–49.96; $P=0.10$), the risk of death or myocardial infarction did not differ in the 2 groups (2.4% versus 1.9%; hazard ratio, 1.21; 95% confidence interval, 0.60–2.47; $P=0.58$). In the prespecified subgroup analysis, target vessel failure occurred more frequently in the 6-month DAPT group than in the 12-month group (hazard ratio, 3.16; 95% confidence interval, 1.42–7.03; $P=0.005$) among diabetic patients.

Conclusions—Six-month DAPT did not increase the risk of target vessel failure at 12 months after implantation of drug-eluting stents compared with 12-month DAPT. However, the noninferiority margin was wide, and the study was underpowered for death or myocardial infarction. Our results need to be confirmed in larger trials.

Clinical Trial Registration—URL: http://www.clinicaltrials.gov. Unique identifier: NCT00698607.

(*Circulation*. 2012;125:505-513.)

Key Words: drug-eluting stents ■ platelet aggregation inhibitors ■ stents ■ thrombosis

Downloaded from http://circ.ahajournals.org/ by guest on January 24, 2017

Several randomized trials have demonstrated that drug-eluting coronary stents reduce angiographic restenosis and target lesion revascularization compared with bare metal stents.[1–3] However, some long-term observational studies have reported that the risk of death or myocardial infarction was higher after drug-eluting stents than after bare metal stents, which may be due to the different incidences of late or very late stent thrombosis.[4,5] Previous observational studies

Received July 29, 2011; accepted November 8, 2011.

From the Division of Cardiology, Department of Medicine, Samsung Medical Center, Sungkyunkwan University School of Medicine, Seoul (H.-C.G., J.-Y.H., Y.B.S., J.-H.C., S.-H.C.); Cardiovascular Center, Seoul National University Main Hospital, Seoul (K.W.P., H.-J.K., B.-K.K., H.-S.K.); Seoul National University Bundang Hospital, Sungnam (I.-H.C.); Korea University Anam Hospital, Seoul (D.-S.L.); Kangdong Sacred Heart Hospital, Seoul (K.-R.H.); Gachon University Gil Medical Center, Incheon (T.A.); Yonsei University Wonju Severance Hospital, Wonju (J.-H.Y.); Chonnam National University Hospital, Gwangju (M.-H.J.); Busan National University Hospital, Busan (T.-J.H.); Seoul National University Boramae Hospital, Seoul (W.-Y.C.); Hallym University Sacred Heart Hospital, Anyang (Y.-J.C.); Keimyung University Dongsan Hospital, Daegu (S.-H.H.); Gangnam Severance Hospital, Seoul (H.-M.K.); NHIC Ilsan Hospital, Goyang (D.-W.J.); Inje University Sanggye Paik Hospital, Seoul (B.-O.K.); Ewha Women's University Mokdong Hospital, Seoul (S.-H.P.); Kangnam Sacred Heart Hospital, Seoul (N.-H.L.); Catholic University Uijeongbu St. Mary's Hospital, Uijeongbu (H.-K.J.); and Yonsei University Severance Hospital, Seoul (Y.J.), Korea.

*Drs Gwon and Hahn contributed equally to this article.

The online-only Data Supplement is available with this article at http://circ.ahajournals.org/lookup/suppl/doi:10.1161/CIRCULATIONAHA.111.059022/-/DC1.

Correspondence to Hyo-Soo Kim, MD, Department of Internal Medicine, Cardiovascular Center, Seoul National University Hospital, 101 DaeHak-ro, JongRo-gu, Seoul, 110–744, Korea. E-mail hyosoo@snu.ac.kr

© 2011 American Heart Association, Inc.

Circulation is available at http://circ.ahajournals.org DOI: 10.1161/CIRCULATIONAHA.111.059022

reported that premature discontinuation of thienopyridine therapy was the major determinant of stent thrombosis after implantation of drug-eluting stents[6] and that the extended use of clopidogrel in patients with drug-eluting stents may be associated with a reduced risk of death or myocardial infarction.[7] From the results of these reports, prolonged dual antiplatelet therapy (DAPT; aspirin plus thienopyridine) of at least 12 months is currently recommended after percutaneous coronary intervention (PCI) with drug-eluting stents unless patients are at high risk for bleeding.[8]

Editorial see p 471
Clinical Perspective on p 513

However, the optimal or minimal necessary duration of DAPT remains undetermined. A randomized trial showed that the use of DAPT for a period >12 months in patients who had received drug-eluting stents was not significantly more effective than aspirin monotherapy.[9] Moreover, some registry studies suggest that DAPT lasting <12 months after PCI with drug-eluting stents does not increase major adverse cardiac events and that there is no apparent clinical benefit from DAPT for >6 months.[10–12] To date, no randomized trials have been performed to compare a shorter duration of DAPT with 12-month DAPT. In the Efficacy of Xience/Promus Versus Cypher to Reduce Late Loss After Stenting (EXCELLENT) trial, we compared 6-month DAPT with 12-month DAPT in patients receiving drug-eluting stents.

Methods

Study Design and Patients

The EXCELLENT trial was a prospective, open-label, randomized trial conducted at 19 sites in Korea. The authors designed the study, and the institutional review board at each participating center approved the trial protocol. The study design has previously been described.[13] Patients were eligible for inclusion in the study if they had at least 1 lesion in a native coronary vessel with a reference diameter of 2.25 to 4.25 mm, stenosis of >50% by visual estimation, and evidence of myocardial ischemia such as stable angina, unstable angina, recent myocardial infarction, silent ischemia, a positive functional study, or reversible changes on ECG consistent with ischemia. Documentation of ischemia was not mandatory for lesions with >75% stenosis. There were no limitations on the number of lesions or the length of the lesions in efforts to reflect real-life clinical practice. Exclusion criteria were myocardial infarction within 72 hours; severely compromised ventricular dysfunction (ejection fraction <25%) or cardiogenic shock; any stent implantation in the target vessel before enrollment; hemoglobin <10 g/dL or platelet count <100 000 per 1 μL; serum creatinine ≥265.2 μmol/L (3.0 mg/dL) or dependence on dialysis; serious hepatic disease; major bleeding within 3 months or major surgery within 2 months; allergy to antiplatelet drugs, heparin, stainless steel, contrast agents, everolimus, or sirolimus; elective surgical procedure planned within <12 months; life expectancy <1 year; significant left main disease defined as stenosis of >50%; chronic total occlusion; true bifurcation lesions requiring a planned 2-stent strategy; or active participation in another clinical study. All patients provided written informed consent.

Study Procedures and Follow-Up

Patients were randomly assigned in a 1:1 ratio to receive either 6-month DAPT (aspirin 100–200 mg/d plus clopidogrel 75 mg/d for 6 months and thereafter aspirin alone) or 12-month DAPT (aspirin 100–200 mg/d plus clopidogrel 75 mg/d for 12 months). Randomization was performed with a Web-based response system after diagnostic angiography and before PCI. Randomization was stratified by the site of enrollment, presence of diabetes mellitus, and lesion length. In addition, patients were randomly assigned to receive everolimus- or sirolimus-eluting stents. The results of the drug-eluting stent arm of the trial are not reported here.

PCI was performed according to standard techniques. Before the index procedure, all patients received at least 300 mg aspirin and a 300- to 600-mg loading dose of clopidogrel unless they had previously received these antiplatelet medications. Unfractionated heparin was administered throughout the procedure to maintain an activated clotting time of ≥250 seconds. Administration of glycoprotein IIb/IIIa inhibitors was at the discretion of the operator. After the procedure, all patients were recommended to receive optimal pharmacological therapy, including statins, β-blockers, or angiotensin-converting enzyme inhibitors at the discretion of the responsible clinicians. Any P2Y12 receptor antagonist other than clopidogrel was not used. Additionally, each investigator was advised to emphasize the importance of cardiovascular risk factor modification to patients.

Clinical follow-up was performed at 1, 3, 6, 9, and 12 months after the index PCI. At follow-up, patient data, including clinical status, all interventions, outcome events, and adverse events, were recorded. In particular, information on the use of aspirin or clopidogrel was assessed at each follow-up.

Study End Points

The primary end point was target vessel failure defined as a composite of cardiac death, myocardial infarction, or target vessel revascularization during the 12-month period after randomization. Secondary end points included the individual components of the primary end point; death resulting from any cause; death or myocardial infarction; stent thrombosis; major bleeding according to the Thrombolysis in Myocardial Infarction criteria[14]; major adverse cardiocerebral events, which were a composite of death, myocardial infarction, stroke, or any revascularization; and a safety end point, which was a composite of death, myocardial infarction, stroke, stent thrombosis, or Thrombolysis in Myocardial Infarction major bleeding.

Clinical events were defined on the basis of the recommendations of the Academic Research Consortium.[15] All deaths were considered cardiac unless a definite noncardiac cause could be established. During the first 48 hours after PCI, myocardial infarction was defined as an increase of cardiac enzyme (creatine kinase-MB fraction or troponin T/troponin I) 3 times above the upper limit of normal in stable patients.[15] In patients with elevated baseline levels of cardiac enzyme, myocardial infarction was defined as a subsequent increase of >2-fold from baseline values.[16] After the first 48 hours, myocardial infarction was defined as the presence of clinical signs of myocardial infarction combined with a creatine kinase-MB fraction or troponin T/troponin I increase higher than the upper limit of normal.[15] Target lesion revascularization was defined as either a repeat PCI of the lesion within 5 mm of the deployed stent or bypass graft surgery of the target vessel. Target vessel revascularization was defined as repeat revascularization of the treated vessel by PCI or bypass graft surgery. Stent thrombosis was defined as definite or probable stent thrombosis according to the Academic Research Consortium classification.[15] Stroke, as detected by the occurrence of a new neurological deficit, was confirmed by a neurologist and on imaging. Device success was defined as the attainment at the target site of a final residual diameter stenosis of <50% using only the assigned study device. Lesion success was defined as the attainment of a final residual diameter stenosis of <50% using any percutaneous method. Procedure success was defined as the attainment at the target site of a final residual diameter stenosis of <50%, together with the absence of any in-hospital major adverse cardiac events. The independent clinical event adjudication committee (Table I in the online-only Data Supplement), the members of which were unaware of the study group assignments, assessed all of the clinical end points.

Downloaded from http://circ.ahajournals.org/ by guest on January 24, 2017

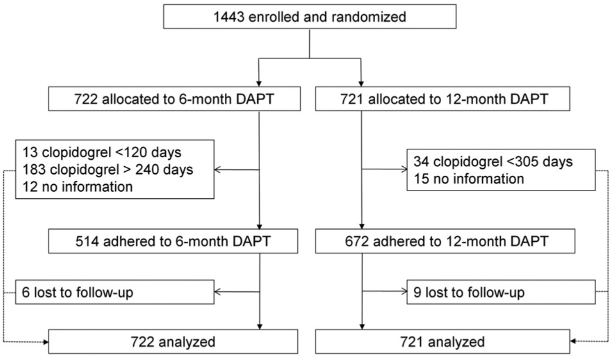

Figure 1. Trial profile. DAPT indicates dual antiplatelet therapy.

Statistical Analysis

The primary analysis was a noninferiority comparison of 6- and 12-month DAPT for the primary end point of target vessel failure according to the intention-to-treat principle. Using data from large, randomized clinical trials evaluating the efficacy of drug-eluting stents, we estimated that the incidence of the primary end point with 12-month DAPT 1 year after the procedure would be 10%.[17,18] The noninferiority margin of 4.0 percentage points was chosen on the basis of historical data,[7] clinically acceptable relevance, and the feasibility of study recruitment. We estimated that with a total of 1372 patients (686 per group), the power of the study would be 80% to show noninferiority with a 1-sided type I error rate of 0.05. Assuming that 5% of patients would be lost to follow-up, we determined the final sample size to be 1440 patients (720 per group).

Continuous variables were presented as mean±SD and compared by use of the Student *t* test. Categorical variables were presented as counts and percentages and compared by use of the χ^2 or Fisher exact test as appropriate. Cumulative event rates were estimated with the Kaplan-Meier method. If the upper limit of the 1-sided 95% confidence interval (CI) of the difference were less than the prespecified noninferiority margin, 6-month DAPT would be considered to be noninferior to 12-month DAPT. Survival curves were compared by use of the log-rank tests. Hazard ratios with 95% CIs were estimated by use of the Cox proportional-hazards method. Landmark analysis[19] was performed with a landmark of clopidogrel discontinuation at 6 months among patients who were event free at 6 months. We also performed per-protocol analysis among patients who adhered to the study protocol. The consistency of treatment effects in prespecified subgroups was assessed by use of Cox regression models with tests for interaction. *P* values and CIs were 2 tailed except those for noninferiority testing of the primary end point. All analyses were performed with SAS version 9.1 (SAS Institute, Inc, Cary, NC).

The authors had full access to and take full responsibility for the integrity of the data. All authors have read and agree to the manuscript as written.

Results

Study Participants

Between June 2008 and July 2009, a total of 1443 patients were enrolled. Of these patients, 722 were assigned to receive 6-month DAPT and 721 were assigned to receive 12-month DAPT (Figure 1). Patients in the 2 groups were well balanced with regard to most baseline demographic and clinical characteristics (Table 1). However, patients with a history of previous myocardial infarction were more common in the 6-month DAPT group compared with the 12-month group (6.5% versus 3.7%; $P=0.02$). Medications at discharge from the index PCI were similar in the 6- and 12-month DAPT groups. Angiographic and procedural data were also similar in the 2 groups (Table 2). Everolimus-eluting stents were used in three quarters of patients and sirolimus-eluting stents were used in one quarter of patients as a result of 3:1 randomization of stents.

Table 1. Baseline Patient Characteristics

	6-mo DAPT (n=722)	12-mo DAPT (n=721)	*P*
Age, y	63.0±9.6	62.4±10.4	0.21
Male sex, n (%)	470 (65.1)	461 (63.9)	0.65
Body mass index, kg/m^2	24.9±3.1	25.1±3.0	0.32
Diabetes mellitus, n (%)	272 (37.7)	278 (38.6)	0.73
Hypertension, n (%)	525 (72.7)	532 (73.8)	0.65
Dyslipidemia, n (%)	543 (75.2)	550 (76.3)	0.63
Current smoker, n (%)	198 (27.4)	186 (25.8)	0.49
Previous myocardial infarction, n (%)	47 (6.5)	27 (3.7)	0.02
Previous PCI, n (%)	67 (9.3)	62 (8.6)	0.65
Previous CABG, n (%)	11 (1.5)	7 (1.0)	0.34
Congestive heart failure, n (%)	4 (0.6)	5 (0.7)	0.75
Chronic renal failure, n (%)	6 (0.8)	9 (1.2)	0.44
Cerebrovascular disease, n (%)	47 (6.5)	48 (6.7)	0.91
Clinical presentation, n (%)			0.56
Silent ischemia/stable angina	353 (48.9)	346 (48.0)	
Unstable angina/non–ST-segment–elevation myocardial infarction	350 (48.5)	349 (48.4)	
ST-elevation myocardial infarction	19 (2.6)	26 (3.6)	
Ejection fraction, %	61.0±9.6	61.6±9.4	0.30
Discharge medications, n (%)			
Aspirin	707 (99.4)	704 (99.0)	0.36
Clopidogrel	702 (98.7)	708 (99.6)	0.08
Statin	604 (85.0)	582 (81.9)	0.12
ACE inhibitor	224 (31.5)	243 (34.2)	0.28
Angiotensin II receptor antagonist	244 (34.3)	231 (32.5)	0.46
β-blocker	427 (60.1)	445 (62.6)	0.33

DAPT indicates dual antiplatelet therapy; PCI, percutaneous coronary intervention; CABG, coronary artery bypass grafting; and ACE, angiotensin-converting enzyme. Data are mean±SD when appropriate. Data are given for the intention-to-treat population.

Study Outcomes

At 12 months, aspirin was continued in 99.9% of the 6-month DAPT group and 99.3% of the 12-month DAPT group. The median duration of DAPT was 190 days (interquartile range, 181–260 days) in the 6-month DAPT group and 375 days (interquartile range, 364–395 days) in the 12-month DAPT group. Adherence to the study protocol was 71.2% of the 6-month DAPT group and 93.2% of the 12-month DAPT group at 12 months.

Follow-up regarding the primary end point was complete in 99.1% of patients in the 6-month DAPT group and 98.8% in the 12-month group. At 12 months, the primary end point of target vessel failure was noted in 34 patients in the 6-month DAPT group and 30 patients in the 12-month group. Cumulative rates of target vessel failure at 1 year were 4.8% for the 6-month and 4.3% for the 12-month DAPT group. The noninferiority of the 6-month DAPT to 12-month DAPT was

Downloaded from http://circ.ahajournals.org/ by guest on January 24, 2017

Table 2. Lesion and Procedural Characteristics

	6-mo DAPT	12-mo DAPT	P
Patients, n	722	721	
Angiographic disease extent, n (%)			0.90
1-Vessel disease	347 (48.1)	346 (48.0)	
2-Vessel disease	226 (31.3)	232 (32.2)	
3-Vessel disease	149 (20.6)	143 (19.8)	
Left anterior descending artery treated, n (%)	452 (63.0)	447 (62.2)	0.73
Use of glycoprotein IIb/IIIa inhibitors, n (%)	12 (1.7)	12 (1.7)	0.99
Use of intravascular ultrasound, n (%)	315 (43.6)	312 (43.3)	0.89
Treated lesions per patient, n	1.3±0.6	1.4±0.5	0.58
Stents per patient, n	1.6±1.0	1.6±0.9	0.39
Type of drug-eluting stents, n (%)			0.99
Everolimus	540 (74.8)	539 (74.8)	
Sirolimus	182 (25.2)	182 (25.2)	
Treated lesions, n	957	970	
Left anterior descending artery, n (%)	482 (50.6)	474 (49.0)	0.51
ACC/AHA lesion class B2/C, n (%)	486 (52.8)	505 (53.8)	0.67
Long lesion (≥20 mm), n (%)	355 (40.3)	374 (41.2)	0.73
Total occlusion, n (%)	39 (4.2)	27 (2.9)	0.11
Thrombotic lesion, n (%)	74 (8.0)	73 (7.8)	0.84
Ulcerative lesion, n (%)	23 (2×4)	16 (1.6)	0.23
Bifurcation lesion, n (%)	98 (10.2)	111 (11.4)	0.42
Stents per lesion, n	1.2±0.5	1.2±0.5	0.41
Stent length per lesion, mm	27.8±13.0	28.3±13.7	0.31
Lesion success, n (%)	941 (99.7)	964 (99.8)	0.64
Device success, n (%)	941 (99.7)	963 (99.7)	0.98
Procedural success, n (%)	935 (99.0)	956 (99.0)	0.66

DAPT indicates dual antiplatelet therapy; ACC, American College of Cardiology; and AHA, American Heart Association. Data are mean±SD when appropriate. Data are given for the intention-to-treat population.

statistically significant (absolute risk difference, 0.5 percentage points; upper limit of 1-sided 95% CI, 2.4%; $P=0.001$ for noninferiority; Table 3 and Figure 2A). Six-month landmark analysis showed that the risk of target vessel failure at 12 months was not significantly higher in the 6-month DAPT group than in the 12-month group (hazard ratio, 1.06; 95% CI, 0.56–2.03; $P=0.85$; Figure 2B). No significant differences were observed between the 2 groups in the secondary end points (Table 3 and Figure 3). Although stent thrombosis tended to occur more frequently in the 6-month DAPT group than in the 12-month group (0.9% versus 0.1%; hazard ratio, 6.02; 95% CI, 0.72–49.96; $P=0.10$), the risk of death or myocardial infarction did not differ between the 2 groups (2.4% versus 1.9%; hazard ratio, 1.21; 95% CI, 0.60–2.47; $P=0.58$). Five of 6 stent thrombosis cases in the 6-month DAPT group occurred before 6 months when patients were taking both aspirin and clopidogrel. In the remaining 1 patient who developed it after 6 months, stent thrombosis occurred 89 days after discontinuation of clopidogrel. In the 12-month DAPT group, there was only 1 case of stent thrombosis, which developed at 7 days after the index procedure (Table 4).

The results from the per-protocol analysis were similar to those from the intention-to-treat analysis. Target vessel failure occurred in 24 of 514 patients in the 6-month DAPT group and 29 of 672 patients in the 12-month DAPT group. Cumulative rates of target vessel failure at 1 year were 4.7% for the 6-month DAPT group and 4.4% for the 12-month DAPT group. The noninferiority of the 6-month DAPT to the 12-month DAPT was also statistically significant (absolute risk difference, 0.3 percentage points; the upper limit of 1-sided 95% CI, 2.3%; $P<0.001$ for noninferiority; Table II and Figure I in the online-only Data Supplement). Although stent thrombosis tended to occur more frequently in the 6-month DAPT group than in 12-month group (1.2% versus 0.2%; hazard ratio, 7.88; 95% CI, 0.95–65.44; $P=0.06$), the risk of death or myocardial infarction did not differ significantly in the 2 groups (2.7% versus 2.1%; hazard ratio, 1.31; 95% CI, 0.63–2.75; $P=0.47$; Table II and Figure II in the online-only Data Supplement).

In prespecified subgroup analysis, the results of comparison between the 2 regimens were consistent across various subgroups (Figure 4). However, there was significant interaction between diabetes mellitus and outcomes (interaction $P<0.001$). Target vessel failure occurred more frequently in the 6-month DAPT group than in the 12-month group among diabetic patients (hazard ratio, 3.16; 95% CI, 1.42–7.03; $P=0.005$), whereas it occurred less frequently in the 6-month DAPT group than in the 12-month group among patients without diabetes mellitus (hazard ratio, 0.44; 95% CI, 0.21–0.94; $P=0.03$). Results of detailed subgroup analysis according to diabetic status are presented in Tables III and IV and Figure IIIA and IIIB in the online-only Data Supplement. Among diabetic patients, rates of myocardial infarction and target vessel revascularization were significantly higher in the 6-month DAPT group than in the 12-month DAPT group (4.5% versus 1.1%; hazard ratio, 4.14; 95% CI, 1.17–14.68; $P=0.03$; and 5.3% versus 1.9%; hazard ratio, 2.91; 95% CI, 1.05–8.08; $P=0.04$, respectively). Stent thrombosis occurred in 4 patients (1.5%) in the 6-month DAPT group compared with none in the 12-month DAPT group among diabetic patients. Although statistical significance was not achieved, the risk of target vessel failure tended to be higher in the 6-month DAPT group than in the 12-month group among patients receiving sirolimus-eluting stents, whereas such a tendency was not observed in those receiving everolimus-eluting stents (P for interaction=0.18). We compared clinical outcomes of 6-month and 12-month DAPT in detail according to the type of stents (Tables V and VI and Figure IIIC and IIID in the online-only Data Supplement). No significant differences were observed in clinical outcomes between the 6- and 12-month DAPT groups among patients receiving everolimus-eluting stents and patients receiving sirolimus-eluting stents.

Discussion

In this prospective, randomized trial, 6-month DAPT was noninferior to 12-month DAPT for the primary end point, the rate of target vessel failure at 12 months. The 6-month landmark analysis and per-protocol analysis showed consistent results. However, target vessel failure occurred more

Downloaded from http://circ.ahajournals.org/ by guest on January 24, 2017

Table 3. Clinical Outcomes

	6-mo DAPT (n=722), n (%)	12-mo DAPT (n=721), n (%)	HR* (95% CI)	*P*
Target vessel failure†	34 (4.8)	30 (4.3)	1.14 (0.70–1.86)	0.60
Total death	4 (0.6)	7 (1.0)	0.57 (0.17–1.95)	0.37
Cardiac death	2 (0.3)	3 (0.4)	0.67 (0.11–3.99)	0.66
Myocardial infarction	13 (1.8)	7 (1.0)	1.86 (0.74–4.67)	0.19
Death/myocardial infarction	17 (2.4)	14 (1.9)	1.21 (0.60–2.47)	0.58
Target vessel myocardial infarction	12 (1.7)	6 (0.8)	2.00 (0.75–5.34)	0.16
Cerebrovascular accident	3 (0.4)	5 (0.7)	0.60 (0.14–2.51)	0.48
Target lesion revascularization	17 (2.4)	18 (2.6)	0.94 (0.49–1.83)	0.86
Target vessel revascularization	22 (3.1)	22 (3.2)	1.00 (0.56–1.81)	0.99
Any revascularization	43 (6.2)	43 (6.2)	1.00 (0.66–1.53)	0.99
Stent thrombosis	6 (0.9)	1 (0.1)	6.02 (0.72–49.96)	0.10
Any bleeding	4 (0.6)	10 (1.4)	0.40 (0.13–1.27)	0.12
TIMI major bleeding‡	2 (0.3)	4 (0.6)	0.50 (0.09–2.73)	0.42
MACCE§	56 (8.0)	60 (8.5)	0.94 (0.65–1.35)	0.72
Safety end point¶	24 (3.3)	21 (3.0)	1.15 (0.64–2.06)	0.64

DAPT indicates dual antiplatelet therapy; HR, hazard ratio; CI, confidence interval; TIMI, Thrombolysis in Myocardial Infarction; and MACCE, major cardiocerebral event. The percentages shown are Kaplan-Meier estimates from the intention-to-treat analysis.

*HRs are for the 6- versus 12-month DAPT group.

†Target vessel failure was a composite of cardiac death, myocardial infarction, or target vessel revascularization.

‡TIMI major bleeding refers to adjudicated events in accordance with previously used TIMI criteria.[14]

§MACCE was a composite of death, myocardial infarction, stroke, or any revascularization.

¶Safety end point was a composite of death, myocardial infarction, stroke, stent thrombosis, or TIMI major bleeding.

Downloaded from http://circ.ahajournals.org/ by guest on January 24, 2017

frequently with 6-month DAPT than with 12-month DAPT among diabetic patients.

Prolonged DAPT of at least 12 months is currently recommended after PCI with drug-eluting stents. Supporting this guideline, several observational studies reported that the risk of death or myocardial infarction increased after drug-eluting stents compared with bare metal stents[4,5] and that the extended use of clopidogrel in patients with drug-eluting stents reduced the risk of death or myocardial infarction.[7] However, DAPT increases bleeding risk[20,21] and costs compared with aspirin alone. Endoscopic, dental, and surgical procedures are often delayed because of prolonged DAPT, which may affect the patient's quality of life.[22] Therefore, determining the optimal or minimal necessary duration of DAPT is very important.

Until now, the premature results of only 1 randomized trial have been reported comparing the clinical outcomes of DAPT versus aspirin alone beyond 12 months, and those results did not support the use of DAPT for a period >12 months in patients receiving drug-eluting stents.[9] The study, however, was not a dedicated randomized trial but a mixture of different cohorts limited by the wide range of the duration of antiplatelet therapy at the time of inclusion, and it was underpowered mainly because of lower rate of primary end point than expected. There has been no prospective randomized trial comparing 12 months and shorter durations of DAPT after drug-eluting stent implantation. Several observational studies reported that discontinuation of thienopyridine therapy beyond 6 months after implantation of drug-eluting stents was not associated with an increased risk of stent thrombosis.[10,11] However, these studies were not randomized trials and were limited by selection bias. In other words, there have been no systematic studies to assess the optimal duration of DAPT. Therefore, we performed a prospective, random-

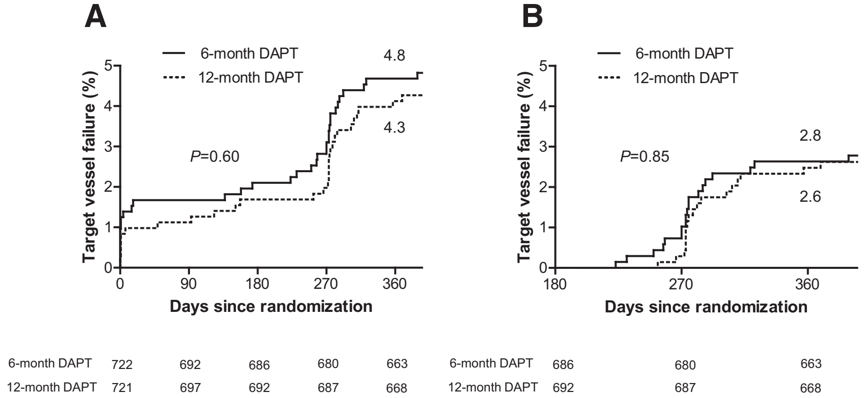

Figure 2. Kaplan-Meier curves for the primary end point of target vessel failure. *P* values were calculated with the log-rank test. **A**, A composite of cardiac death, myocardial infarction, or target vessel revascularization by intention-to-treat analysis. **B**, Six-month landmark analysis among patients who were event-free at 6 months. DAPT indicates dual antiplatelet therapy.

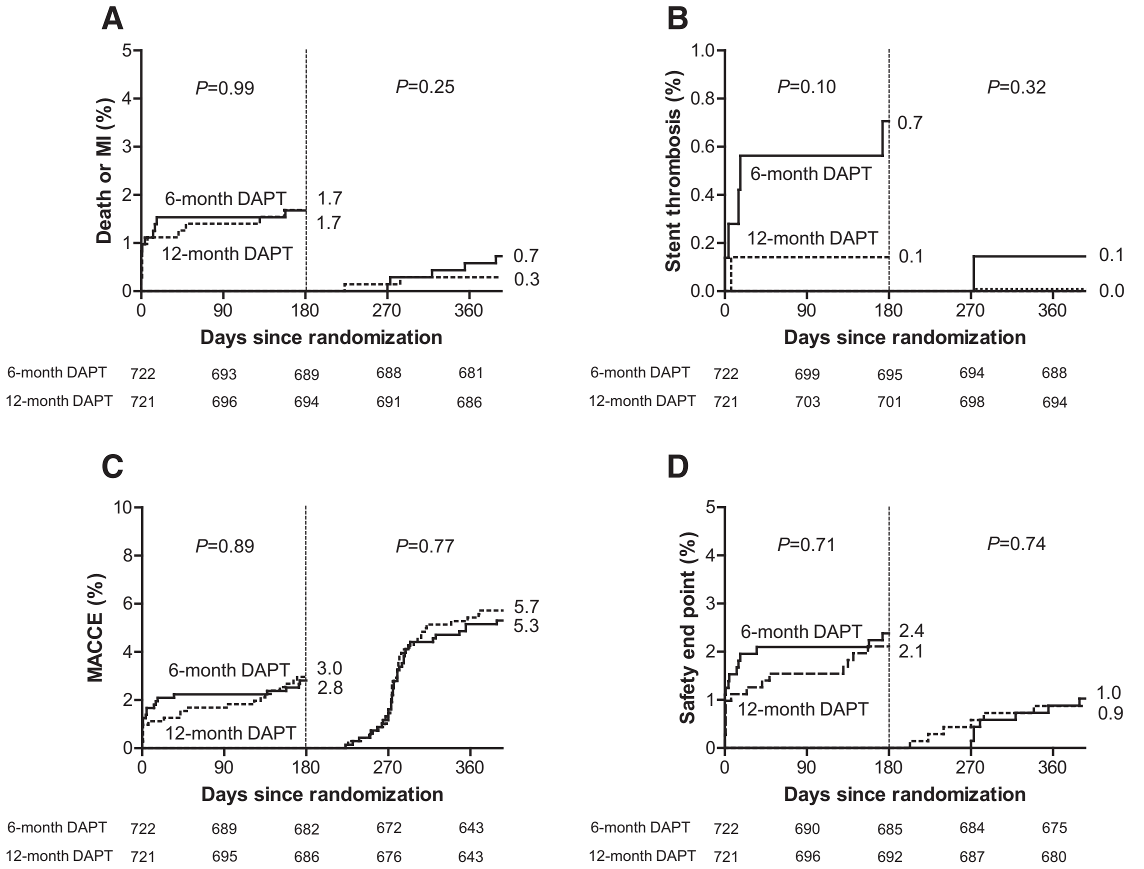

Figure 3. Six-month landmark analysis for the key secondary end points. *P* values were calculated with the log-rank test. **A**, A composite of death or myocardial infarction (MI). **B**, Stent thrombosis. **C**, Major adverse cardiocerebral events (MACCE; a composite of death, MI, stroke, or any revascularization). **D**, Safety end point (a composite of death, MI, stroke, stent thrombosis, or Thrombolysis in Myocardial Infarction major bleeding). DAPT indicates dual antiplatelet therapy.

ized study to compare 6- and 12-month DAPT in patients receiving drug-eluting stents.

The main finding of our study was that the cumulative incidences of target vessel failure did not differ significantly between 6-month and 12-month DAPT. In addition, there were no significant differences in the secondary end points such as death/myocardial infarction, revascularization, or major adverse cardiocerebral events. Our results confirm the results of previous registry data that there were no apparent clinical benefits from DAPT for >6 months.[10–12] However, stent thrombosis tended to occur more frequently in the 6-month DAPT group than in the 12-month group. The power of our study was insufficient to reach conclusions regarding the relationship between stent thrombosis and duration of

Table 4. Detailed Information on Stent Thrombosis

Time to Stent Thrombosis, d	Classification	Group	Clinical Presentation	Diabetic Status	Ejection Fraction, %	Stent Type	Aspirin	Clopidogrel	Outcome
0	Definite	6-mo DAPT	ST-segment–elevation myocardial infarction	No	55	EES	Continued	Continued	TLR
4	Definite	6-mo DAPT	Stable angina	Yes (OHA treated)	58	SES	Continued	Continued	Myocardial infarction
7	Probable	12-mo DAPT	Unstable angina	No	74	EES	Continued	Continued	Death
15	Definite	6-mo DAPT	Non–ST-segment–elevation myocardial infarction	Yes (OHA treated)	62	EES	Continued	Continued	Myocardial infarction
17	Definite	6-mo DAPT	Stable angina	Yes (OHA treated)	70	SES	Continued	Continued	Myocardial infarction
173	Definite	6-mo DAPT	Stable angina	Yes (OHA treated)	Not available	EES	Continued	Continued	TLR
273	Definite	6-mo DAPT	Stable angina	No	70	SES	Continued	Discontinued at day 184	TLR

DAPT indicates dual antiplatelet therapy; EES, everolimus-eluting stent; TLR, target lesion revascularization; OHA, oral hypoglycemic agents; and SES, sirolimus-eluting stent.

Downloaded from http://circ.ahajournals.org/ by guest on January 24, 2017

	Total	6-mo DAPT n (%)	12-mo DAPT n (%)	HR (95% CI)	*P* value	Interaction *P* value
**Age**						
<65	767	19 (5.1)	12 (3.2)	1.61 (0.78-3.31)	0.20	0.19
≥65	676	15 (4.5)	18 (5.5)	0.83 (0.42-1.65)	0.59	
**ACS**						
No	699	21 (6.03)	13 (3.82)	1.61 (0.8-3.21)	0.18	0.15
Yes	744	13 (3.65)	17 (4.69)	0.78 (0.38-1.60)	0.50	
**Diabetes**						
No	893	10 (2.27)	22 (5.08)	0.44 (0.21-0.94)	0.03	<0.001
Yes	550	24 (9.09)	8 (2.96)	3.16 (1.42-7.03)	0.005	
**LV ejection fraction**						
≥50%	1097	26 (4.91)	24 (4.42)	1.12 (0.64-1.95)	0.69	0.27
<50%	124	2 (3.08)	4 (7.41)	0.41 (0.07-2.23)	0.30	
**Bifurcation**						
No	1243	27 (4.41)	22 (3.65)	1.22 (0.69-2.14)	0.49	0.70
Yes	200	7 (7.61)	8 (7.96)	0.97 (0.35-2.67)	0.95	
**Type of stent**						
EES	1079	25 (4.72)	26 (4.94)	0.96 (0.55-1.66)	0.89	0.18
SES	364	9 (5.14)	4 (2.27)	2.31 (0.71-7.5)	0.16	
**Multi-stent**						
No	866	14 (3.18)	12 (2.91)	1.1 (0.51-2.38)	0.81	0.80
Yes	567	20 (7.68)	18 (6.2)	1.25 (0.66-2.37)	0.49	

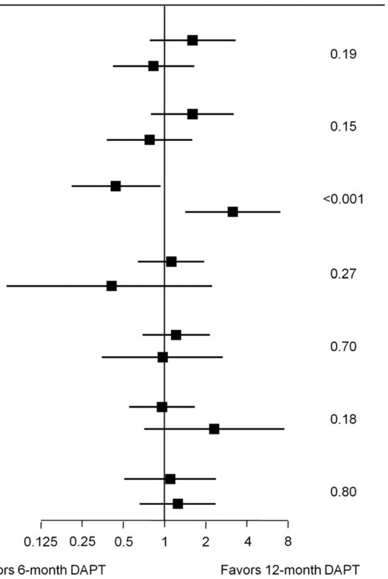

Figure 4. Subgroup analyses of the primary end point. DAPT indicates dual antiplatelet therapy; HR, hazard ratio; CI, confidence interval; ACS, acute coronary syndrome; LV, left ventricular; EES, everolimus-eluting stent; and SES, sirolimus-eluting stent.

DAPT. Prolonged DAPT for >6 months might be needed to prevent late stent thrombosis because of delayed vascular healing and inflammatory reaction after implantation of drug-eluting stents.[23,24] However, the majority of patients were taking both aspirin and clopidogrel at the time of stent thrombosis. Moreover, the timing of discontinuation of clopidogrel and stent thrombosis in our study called into question the temporal and causal relationship between discontinuation and thrombosis.

The interesting result in this study is that the treatment effect varied depending on the presence of diabetes mellitus. Among diabetic patients, target vessel failure occurred more frequently with 6-month DAPT than with 12-month DAPT. Although these results might have occurred by chance, several lines of evidences support our result. Diabetes mellitus is regarded as a proinflammatory and prothrombotic condition,[25,26] and patients with diabetes mellitus are more frequently resistant to aspirin than those without diabetes mellitus.[27] In an observational study of diabetes mellitus, longer use of clopidogrel was associated with a lower incidence of death or myocardial infarction after implantation of drug-eluting stents.[28] The minimum necessary duration of DAPT may be longer in diabetic than in nondiabetic patients. Although a significant interaction between other conditions and outcomes was not found, results from diabetic patients can suggest that longer DAPT may be needed in high-risk patients. These findings, however, should be interpreted with caution, although they were derived from the prespecified subgroup analysis.

There were several limitations to our study. First, the primary end point was target vessel failure rather than hard end points such as death or myocardial infarction. Considering the low rate of death or myocardial infarction in the present study, tens of thousands of patients need to be enrolled to compare death or myocardial infarction as the primary end point. However, we do not think that including revascularization could have biased our findings toward a neutral effect on outcomes because it is unlikely that a shorter duration of DAPT is superior to a longer one in terms of revascularization. Revascularization is also one of the important outcomes and might be included in the primary end point to test noninferiority of 6-month DAPT more rigorously. Second, the event rate was lower than expected, although estimates of the event rate were based on data from previous studies.[17,18] As a result, the noninferiority margin of 4.0 percentage points was quite wide, considering that the rate of target vessel failure was 4.3% with 12-month antiplatelet therapy. The low event rate might be explained by several possible factors. Our study was not an all-comer study, and patients with high risk such as those with myocardial infarction within 72 hours, left main lesions, or severe left ventricular dysfunction were excluded. Differences in interventional practice such as frequent use of intravascular ultrasound may play a role. Ethnic differences between our study and previous ones may be another potential contributor. Third, our study was an open-label trial and was not placebo controlled. This can affect study outcomes, including target vessel revascularization, which was 1 component of the primary end point, target vessel failure. Although all clinical end points were assessed by members of independent clinical event adjudication committee and statistical analyses were performed by independent statisticians, operators were not blinded to duration of clopidogrel. Fourth, apart from the American College of Cardiology/American Heart Association guideline for PCI,[8] clopidogrel is recommended for at least 12 months in patients receiving stent during PCI for acute coronary syndromes.[29] Patients who initially presented with acute coronary syndromes and were allocated to the 6-month DAPT group might be at higher risk of events than those whose initial presentation was stable angina. Finally, a considerable proportion of patients in the 6-month DAPT group received clopidogrel for >6 months. However, 6-month DAPT was also noninferior to 12-month DAPT in the per-protocol analysis. Larger ongoing randomized trial

Downloaded from http://circ.ahajournals.org/ by guest on January 24, 2017

such as the Safety and Efficacy of Six Months DAPT After Drug-Eluting Stenting (ISAR-SAFE) trial (NCT00661206) can provide more evidence regarding the safety of 6-month DAPT. Until more confirmative evidence of the safety of 6-month DAPT is obtained, 6-month DAPT cannot be recommended in the general population undergoing PCI. Meanwhile, our results may be helpful for physicians to decide the duration of DAPT case by case in real-world practice, eg, in patients with increased bleeding risk or undergoing elective surgery.

Conclusions

Our trial showed that the rate of target vessel failure was not significantly different between the 6- and 12-month DAPT groups after PCI with drug-eluting stents and that 6-month DAPT was noninferior to 12-month DAPT in the risk of target vessel failure. However, the noninferiority margin was wide, and the study was underpowered for hard end points such as death or myocardial infarction. The safety of a short duration of DAPT in terms of stent thrombosis or in diabetic patients should be studied in larger randomized trials.

Sources of Funding

This study was supported by a grant (A040152) from the Ministry of Health, Welfare, and Family Affairs, Korea; Abbott Vascular Korea; and Boston Scientific Korea. The sponsors had no access to the study data and had no role in the design, conduct, analysis, or reporting of the study.

Disclosures

None.

References

1. Moses JW, Leon MB, Popma JJ, Fitzgerald PJ, Holmes DR, O'Shaughnessy C, Caputo RP, Kereiakes DJ, Williams DO, Teirstein PS, Jaeger JL, Kuntz RE. Sirolimus-eluting stents versus standard stents in patients with stenosis in a native coronary artery. *N Engl J Med*. 2003;349:1315–1323.
2. Stone GW, Ellis SG, Cox DA, Hermiller J, O'Shaughnessy C, Mann JT, Turco M, Caputo R, Bergin P, Greenberg J, Popma JJ, Russell ME. A polymer-based, paclitaxel-eluting stent in patients with coronary artery disease. *N Engl J Med*. 2004;350:221–231.
3. Fajadet J, Wijns W, Laarman GJ, Kuck KH, Ormiston J, Munzel T, Popma JJ, Fitzgerald PJ, Bonan R, Kuntz RE. Randomized, double-blind, multicenter study of the Endeavor zotarolimus-eluting phosphorylcholine-encapsulated stent for treatment of native coronary artery lesions: clinical and angiographic results of the ENDEAVOR II trial. *Circulation*. 2006;114:798–806.
4. Pfisterer M, Brunner-La Rocca HP, Buser PT, Rickenbacher P, Hunziker P, Mueller C, Jeger R, Bader F, Osswald S, Kaiser C. Late clinical events after clopidogrel discontinuation may limit the benefit of drug-eluting stents: an observational study of drug-eluting versus bare-metal stents. *J Am Coll Cardiol*. 2006;48:2584–2591.
5. Daemen J, Wenaweser P, Tsuchida K, Abrecht L, Vaina S, Morger C, Kukreja N, Juni P, Sianos G, Hellige G, van Domburg RT, Hess OM, Boersma E, Meier B, Windecker S, Serruys PW. Early and late coronary stent thrombosis of sirolimus-eluting and paclitaxel-eluting stents in routine clinical practice: data from a large two-institutional cohort study. *Lancet*. 2007;369:667–678.
6. Iakovou I, Schmidt T, Bonizzoni E, Ge L, Sangiorgi GM, Stankovic G, Airoldi F, Chieffo A, Montorfano M, Carlino M, Michev I, Corvaja N, Briguori C, Gerckens U, Grube E, Colombo A. Incidence, predictors, and outcome of thrombosis after successful implantation of drug-eluting stents. *JAMA*. 2005;293:2126–2130.
7. Eisenstein EL, Anstrom KJ, Kong DF, Shaw LK, Tuttle RH, Mark DB, Kramer JM, Harrington RA, Matchar DB, Kandzari DE, Peterson ED, Schulman KA, Califf RM. Clopidogrel use and long-term clinical outcomes after drug-eluting stent implantation. *JAMA*. 2007;297:159–168.
8. King SB 3rd, Smith SC Jr, Hirshfeld JW Jr, Jacobs AK, Morrison DA, Williams DO, Feldman TE, Kern MJ, O'Neill WW, Schaff HV, Whitlow PL, Adams CD, Anderson JL, Buller CE, Creager MA, Ettinger SM, Halperin JL, Hunt SA, Krumholz HM, Kushner FG, Lytle BW, Nishimura R, Page RL, Riegel B, Tarkington LG, Yancy CW. 2007 Focused update of the ACC/AHA/SCAI 2005 guideline update for percutaneous coronary intervention: a report of the American College of Cardiology/American Heart Association Task Force on Practice Guidelines: 2007 Writing Group to Review New Evidence and Update the ACC/AHA/SCAI 2005 Guideline Update for Percutaneous Coronary Intervention, writing on behalf of the 2005 writing committee. *Circulation*. 2008;117:261–295.
9. Park SJ, Park DW, Kim YH, Kang SJ, Lee SW, Lee CW, Han KH, Park SW, Yun SC, Lee SG, Rha SW, Seong IW, Jeong MH, Hur SH, Lee NH, Yoon J, Yang JY, Lee BK, Choi YJ, Chung WS, Lim DS, Cheong SS, Kim KS, Chae JK, Nah DY, Jeon DS, Seung KB, Jang JS, Park HS, Lee K. Duration of dual antiplatelet therapy after implantation of drug-eluting stents. *N Engl J Med*. 2010;362:1374–1382.
10. Airoldi F, Colombo A, Morici N, Latib A, Cosgrave J, Buellesfeld L, Bonizzoni E, Carlino M, Gerckens U, Godino C, Melzi G, Michev I, Montorfano M, Sangiorgi GM, Qasim A, Chieffo A, Briguori C, Grube E. Incidence and predictors of drug-eluting stent thrombosis during and after discontinuation of thienopyridine treatment. *Circulation*. 2007;116:745–754.
11. Kimura T, Morimoto T, Nakagawa Y, Tamura T, Kadota K, Yasumoto H, Nishikawa H, Hiasa Y, Muramatsu T, Meguro T, Inoue N, Honda H, Hayashi Y, Miyazaki S, Oshima S, Honda T, Shiode N, Namura M, Sone T, Nobuyoshi M, Kita T, Mitsudo K. Antiplatelet therapy and stent thrombosis after sirolimus-eluting stent implantation. *Circulation*. 2009;119:987–995.
12. Hahn JY, Song YB, Choi JH, Choi SH, Lee SY, Park HS, Hur SH, Lee S, Han KR, Rha SW, Cho BR, Park JS, Yoon J, Lim do S, Lee SH, Gwon HC. Three-month dual antiplatelet therapy after implantation of zotarolimus-eluting stents: the DATE (Duration of Dual Antiplatelet Therapy After Implantation of Endeavor Stent) registry. *Circ J*. 2010;74:2314–2321.
13. Park KW, Yoon JH, Kim JS, Hahn JY, Cho YS, Chae IH, Gwon HC, Ahn T, Oh BH, Park JE, Shim WH, Shin EK, Jang YS, Kim HS. Efficacy of Xience/promus versus Cypher in rEducing Late Loss after stENTing (EXCELLENT) trial: study design and rationale of a Korean multicenter prospective randomized trial. *Am Heart J*. 2009;157:811–817.e1.
14. Bovill EG, Terrin ML, Stump DC, Berke AD, Frederick M, Collen D, Feit F, Gore JM, Hillis LD, Lambrew CT, et al. Hemorrhagic events during therapy with recombinant tissue-type plasminogen activator, heparin, and aspirin for acute myocardial infarction: results of the Thrombolysis in Myocardial Infarction (TIMI), phase II trial. *Ann Intern Med*. 1991;115:256–265.
15. Cutlip DE, Windecker S, Mehran R, Boam A, Cohen DJ, van Es GA, Steg PG, Morel MA, Mauri L, Vranckx P, McFadden E, Lansky A, Hamon M, Krucoff MW, Serruys PW. Clinical end points in coronary stent trials: a case for standardized definitions. *Circulation*. 2007;115:2344–2351.
16. Fox KA, Poole-Wilson PA, Henderson RA, Clayton TC, Chamberlain DA, Shaw TR, Wheatley DJ, Pocock SJ. Interventional versus conservative treatment for patients with unstable angina or non-ST-elevation myocardial infarction: the British Heart Foundation RITA 3 randomised trial: Randomized Intervention Trial of unstable Angina. *Lancet*. 2002;360:743–751.
17. Windecker S, Remondino A, Eberli FR, Juni P, Raber L, Wenaweser P, Togni M, Billinger M, Tuller D, Seiler C, Roffi M, Corti R, Sutsch G, Maier W, Luscher T, Hess OM, Egger M, Meier B. Sirolimus-eluting and paclitaxel-eluting stents for coronary revascularization. *N Engl J Med*. 2005;353:653–662.
18. Morice MC, Colombo A, Meier B, Serruys P, Tamburino C, Guagliumi G, Sousa E, Stoll HP. Sirolimus- vs paclitaxel-eluting stents in de novo coronary artery lesions: the REALITY trial: a randomized controlled trial. *JAMA*. 2006;295:895–904.
19. Anderson JR, Cain KC, Gelber RD. Analysis of survival by tumor response. *J Clin Oncol*. 1983;1:710–719.
20. Yusuf S, Zhao F, Mehta SR, Chrolavicius S, Tognoni G, Fox KK. Effects of clopidogrel in addition to aspirin in patients with acute coronary syndromes without ST-segment elevation. *N Engl J Med*. 2001;345:494–502.

Downloaded from http://circ.ahajournals.org/ by guest on January 24, 2017

21. Hallas J, Dall M, Andries A, Andersen BS, Aalykke C, Hansen JM, Andersen M, Lassen AT. Use of single and combined antithrombotic therapy and risk of serious upper gastrointestinal bleeding: population based case-control study. *BMJ*. 2006;333:726.
22. Iwata Y, Kobayashi Y, Fukushima K, Kitahara H, Asano T, Ishio N, Nakayama T, Kuroda N, Komuro I. Incidence of premature discontinuation of antiplatelet therapy after sirolimus-eluting stent implantation. *Circ J*. 2008;72:340–341.
23. Finn AV, Joner M, Nakazawa G, Kolodgie F, Newell J, John MC, Gold HK, Virmani R. Pathological correlates of late drug-eluting stent thrombosis: strut coverage as a marker of endothelialization. *Circulation*. 2007;115:2435–2441.
24. Farb A, Burke AP, Kolodgie FD, Virmani R. Pathological mechanisms of fatal late coronary stent thrombosis in humans. *Circulation*. 2003;108:1701–1706.
25. Dichiara J, Bliden KP, Tantry US, Chaganti SK, Kreutz RP, Gesheff TB, Kreutz Y, Gurbel PA. Platelet function measured by VerifyNow identifies generalized high platelet reactivity in aspirin treated patients. *Platelets*. 2007;18:414–423.
26. DiChiara J, Bliden KP, Tantry US, Hamed MS, Antonino MJ, Suarez TA, Bailon O, Singla A, Gurbel PA. The effect of aspirin dosing on platelet function in diabetic and nondiabetic patients: an analysis from the Aspirin-Induced Platelet Effect (ASPECT) study. *Diabetes*. 2007;56:3014–3019.
27. Angiolillo DJ, Fernandez-Ortiz A, Bernardo E, Ramirez C, Sabate M, Jimenez-Quevedo P, Hernandez R, Moreno R, Escaned J, Alfonso F, Banuelos C, Costa MA, Bass TA, Macaya C. Influence of aspirin resistance on platelet function profiles in patients on long-term aspirin and clopidogrel after percutaneous coronary intervention. *Am J Cardiol*. 2006;97:38–43.
28. Brar SS, Kim J, Brar SK, Zadegan R, Ree M, Liu IL, Mansukhani P, Aharonian V, Hyett R, Shen AY. Long-term outcomes by clopidogrel duration and stent type in a diabetic population with de novo coronary artery lesions. *J Am Coll Cardiol*. 2008;51:2220–2227.
29. Kushner FG, Hand M, Smith SC Jr, King SB 3rd, Anderson JL, Antman EM, Bailey SR, Bates ER, Blankenship JC, Casey DE Jr, Green LA, Hochman JS, Jacobs AK, Krumholz HM, Morrison DA, Ornato JP, Pearle DL, Peterson ED, Sloan MA, Whitlow PL, Williams DO. 2009 Focused updates: ACC/AHA guidelines for the management of patients with ST-elevation myocardial infarction (updating the 2004 guideline and 2007 focused update) and ACC/AHA/SCAI guidelines on percutaneous coronary intervention (updating the 2005 guideline and 2007 focused update): a report of the American College of Cardiology Foundation/American Heart Association Task Force on Practice Guidelines. *Circulation*. 2009;120:2271–2306.

CLINICAL PERSPECTIVE

The optimal duration of dual antiplatelet therapy (DAPT) after implantation of drug-eluting coronary stents remains undetermined. Although premature discontinuation of thienopyridine therapy was reported to be the major determinant of stent thrombosis after implantation of drug-eluting stents, some studies suggest that there is no apparent clinical benefit from DAPT for >6 months. In the Efficacy of Xience/Promus Versus Cypher to Reduce Late Loss After Stenting (EXCELLENT) trial, we compared 6-month DAPT with 12-month DAPT in patients receiving drug-eluting stents. Our trial showed that the rate of target vessel failure was not significantly different between the 6- and 12-month DAPT groups after percutaneous coronary intervention with drug-eluting stents (4.8% versus 4.3%) and that 6-month DAPT was noninferior to 12-month DAPT in the risk of target vessel failure. However, stent thrombosis tended to occur more frequently in the 6-month DAPT group than in the 12-month group (0.9% versus 0.1%). In subgroup analysis, target vessel failure occurred more frequently in the 6-month DAPT group than in the 12-month group among diabetic patients (hazard ratio, 3.16; 95% confidence interval, 1.42–7.03). Although 6-month DAPT cannot be recommended in the general population on the basis of our trial, these data may be helpful for physicians to decide the duration of DAPT case by case in real-world practice, eg, in patients with increased bleeding risk or undergoing elective surgery.

Downloaded from http://circ.ahajournals.org/ by guest on January 24, 2017

Circulation

American Heart Association

Six-Month Versus 12-Month Dual Antiplatelet Therapy After Implantation of Drug-Eluting Stents: The Efficacy of Xience/Promus Versus Cypher to Reduce Late Loss After Stenting (EXCELLENT) Randomized, Multicenter Study

Hyeon-Cheol Gwon, Joo-Yong Hahn, Kyung Woo Park, Young Bin Song, In-Ho Chae, Do-Sun Lim, Kyoo-Rok Han, Jin-Ho Choi, Seung-Hyuk Choi, Hyun-Jae Kang, Bon-Kwon Koo, Taehoon Ahn, Jung-Han Yoon, Myung-Ho Jeong, Taek-Jong Hong, Woo-Young Chung, Young-Jin Choi, Seung-Ho Hur, Hyuck-Moon Kwon, Dong-Woon Jeon, Byung-Ok Kim, Si-Hoon Park, Nam-Ho Lee, Hui-Kyung Jeon, Yangsoo Jang and Hyo-Soo Kim

Circulation. 2012;125:505-513; originally published online December 16, 2011;
doi: 10.1161/CIRCULATIONAHA.111.059022
Circulation is published by the American Heart Association, 7272 Greenville Avenue, Dallas, TX 75231
Copyright © 2011 American Heart Association, Inc. All rights reserved.
Print ISSN: 0009-7322. Online ISSN: 1524-4539

The online version of this article, along with updated information and services, is located on the World Wide Web at:
http://circ.ahajournals.org/content/125/3/505

Data Supplement (unedited) at:
http://circ.ahajournals.org/content/suppl/2011/12/16/CIRCULATIONAHA.111.059022.DC1.html
http://circ.ahajournals.org/content/suppl/2013/10/14/CIRCULATIONAHA.111.059022.DC2.html

Permissions: Requests for permissions to reproduce figures, tables, or portions of articles originally published in *Circulation* can be obtained via RightsLink, a service of the Copyright Clearance Center, not the Editorial Office. Once the online version of the published article for which permission is being requested is located, click Request Permissions in the middle column of the Web page under Services. Further information about this process is available in the Permissions and Rights Question and Answer document.

Reprints: Information about reprints can be found online at:
http://www.lww.com/reprints

Subscriptions: Information about subscribing to *Circulation* is online at:
http://circ.ahajournals.org//subscriptions/

Downloaded from http://circ.ahajournals.org/ by guest on January 24, 2017

SUPPLEMENTAL MATERIAL

Supplemental Table 1. Clinical event adjudication committee

Name	Affiliation
Seok-Jae Hwang	Division of Cardiology, Department of Internal Medicine, Gyeongsang National University Hospital, Jinju, Republic of Korea
Kyounghoon Lee	Department of Cardiology, Gil Medical Center, Gachon University of Medicine and Science, Incheon, Republic of Korea
Chan Il Moon	Department of Cardiology, Gil Medical Center, Gachon University of Medicine and Science, Incheon, Republic of Korea

Supplemental Table 2. Clinical outcomes by per-protocol analysis

	6-month DAPT (n=514)	12-month DAPT (n=672)	HR (95% CI)†	*P*
Target-vessel failure†	24 (4.7)	29 (4.4)	1.09 (0.63-1.87)	0.76
Total death	4 (0.8)	7 (1.1)	0.75 (0.22-2.56)	0.64
Cardiac death	2 (0.4)	3 (0.5)	0.87 (0.15-5.22)	0.88
Myocardial infarction	10 (2.0)	7 (1.1)	1.87 (0.71-4.92)	0.20
Death / myocardial infarction	14 (2.7)	14 (2.1)	1.31 (0.63-2.75)	0.47
Target vessel myocardial infarction	9 (1.8)	6 (0.9)	1.97 (0.70-5.52)	0.20
Cerebrovascular accident	3 (0.6)	5 (0.8)	0.79 (0.19-3.28)	0.74
Target-lesion revascularization	10 (2.0)	17 (2.6)	0.77 (0.35-1.68)	0.51
Target-vessel revascularization	14 (2.8)	21 (3.2)	0.88 (0.45-1.72)	0.70
Any revascularization	29 (5.7)	42 (6.4)	0.90 (0.56-1.45)	0.67
Stent thrombosis	6 (1.2)	1 (0.2)	7.88 (0.95-65.44)	0.06
Any bleeding	4 (0.8)	9 (1.4)	0.58 (0.18-1.89)	0.37
TIMI major bleeding‡	2 (0.4)	4 (0.6)	0.66 (0.12-3.60)	0.63
MACCE§	42 (8.2)	59 (8.8)	0.93 (0.63-1.38)	0.72
Safety endpoint¶	21 (4.1)	21 (3.1)	1.32 (0.72-2.41)	0.37

Data are n (%). The percentages shown are Kaplan–Meier estimates from the per-protocol analysis. DAPT indicates dual antiplatelet therapy.

*Hazard ratios (HR) are for the 6-month dual antiplatelet therapy group as compared with the 12-month dual antiplatelet therapy group.

†Target vessel failure was a composite of cardiac death, myocardial infarction, or target vessel revascularization.

‡Thrombolysis in Myocardial Infarction (TIMI) major bleeding refers to adjudicated events in accordance with previously used TIMI criteria.

§Major adverse cardiocerebral event (MACCE) was a composite of death, myocardial infarction, stroke, or any revascularization.

¶Safety endpoint was a composite of death, myocardial infarction, stroke, stent thrombosis, or TIMI major bleeding.

Supplemental Table 3. Clinical outcomes among diabetic patients

	6-month DAPT (n=272)	12-month DAPT (n=278)	HR (95% CI)†	*P*
Target-vessel failure†	24 (9.1)	8 (3.0)	3.16 (1.42-7.03)	0.005
Total death	2 (0.8)	3 (1.1)	0.68 (0.11-4.08)	0.68
Cardiac death	1 (0.4)	1 (0.4)	1.02 (0.06-16.32)	0.99
Myocardial infarction	12 (4.5)	3 (1.1)	4.14 (1.17-14.68)	0.03
Death / myocardial infarction	14 (5.3)	6 (2.2)	2.42 (0.93-6.31)	0.07
Target vessel myocardial infarction	11 (4.1)	2 (0.7)	5.68 (1.26-25.63)	0.02
Cerebrovascular accident	1 (0.4)	2 (0.7)	0.51 (0.05-5.64)	0.58
Target-lesion revascularization	11 (4.2)	5 (1.9)	2.27 (0.79-6.54)	0.13
Target-vessel revascularization	14 (5.3)	5 (1.9)	2.91 (1.05-8.08)	0.04
Any revascularization	23 (8.8)	14 (5.2)	1.71 (0.88-3.33)	0.11
Stent thrombosis	4 (1.5)	0 (0)	-	0.31
Any bleeding	0 (0)	4 (1.5)	-	0.31
TIMI major bleeding‡	0 (0)	0 (0)	-	-
MACCE§	33 (12.5)	21 (7.8)	1.66 (0.96-2.86)	0.07
Safety endpoint¶	16 (6.0)	8 (2.9)	2.08 (0.89-4.86)	0.09

Data are n (%). The percentages shown are Kaplan–Meier estimates from the intention-to-treat analysis. DAPT indicates dual antiplatelet therapy.

*Hazard ratios (HR) are for the 6-month dual antiplatelet therapy group as compared with the 12-month dual antiplatelet therapy group.

†Target vessel failure was a composite of cardiac death, myocardial infarction, or target vessel revascularization.

‡Thrombolysis in Myocardial Infarction (TIMI) major bleeding refers to adjudicated events in accordance with previously used TIMI criteria.[14]

§Major adverse cardiocerebral event (MACCE) was a composite of death, myocardial infarction, stroke, or any revascularization.

¶Safety endpoint was a composite of death, myocardial infarction, stroke, stent thrombosis, or TIMI major bleeding.

Supplemental Table 4. Clinical outcomes among non-diabetic patients

	6-month DAPT (n=450)	12-month DAPT (n=443)	HR (95% CI)†	*P*
Target-vessel failure†	10 (2.3)	22 (5.1)	0.44 (0.21-0.94)	0.03
Total death	2 (0.5)	4 (0.9)	0.49 (0.09-2.70)	0.42
Cardiac death	1 (0.2)	2 (0.5)	0.49 (0.05-5.45)	0.57
Myocardial infarction	1 (0.2)	4 (0.9)	0.25 (0.03-2.21)	0.21
Death / myocardial infarction	3 (0.7)	8 (1.8)	0.37 (0.10-1.39)	0.14
Target vessel myocardial infarction	1 (0.2)	4 (0.9)	0.25 (0.03-2.21)	0.21
Cerebrovascular accident	2 (0.5)	3 (0.7)	0.66 (0.11-3.93)	0.65
Target-lesion revascularization	6 (1.4)	13 (3.0)	0.45 (0.17-1.19)	0.11
Target-vessel revascularization	8 (1.8)	17 (4.0)	0.46 (0.20-1.07)	0.07
Any revascularization	20 (4.6)	29 (6.8)	0.68 (0.38-1.19)	0.18
Stent thrombosis	2 (0.5)	1 (0.2)	2.00 (0.18-21.81)	0.58
Any bleeding	4 (0.9)	6 (1.4)	0.66 (0.19-2.33)	0.52
TIMI major bleeding‡	2 (0.5)	4 (0.9)	0.49 (0.09-2.69)	0.41
MACCE§	23 (5.2)	39 (9.0)	0.57 (0.34-0.96)	0.03
Safety endpoint¶	8 (1.8)	13 (3.0)	0.61 (0.25-3.67)	0.27

Data are n (%). The percentages shown are Kaplan–Meier estimates from the intention-to-treat analysis. DAPT indicates dual antiplatelet therapy.

*Hazard ratios (HR) are for the 6-month dual antiplatelet therapy group as compared with the 12-month dual antiplatelet therapy group.

†Target vessel failure was a composite of cardiac death, myocardial infarction, or target vessel revascularization.

‡Thrombolysis in Myocardial Infarction (TIMI) major bleeding refers to adjudicated events in accordance with previously used TIMI criteria.[14]

§Major adverse cardiocerebral event (MACCE) was a composite of death, myocardial infarction, stroke, or any revascularization.

¶Safety endpoint was a composite of death, myocardial infarction, stroke, stent thrombosis, or TIMI major bleeding.

Supplemental Table 5. Clinical outcomes among patients receiving everolimus-eluting stents

	6-month DAPT (n=540)	12-month DAPT (n=539)	HR (95% CI)†	*P*
Target-vessel failure†	25 (4.7)	26 (4.9)	0.96 (0.56-1.66)	0.89
Total death	3 (0.6)	4 (0.8)	0.75 (0.17-3.34)	0.70
Cardiac death	1 (0.2)	2 (0.4)	0.50 (0.05-5.49)	0.57
Myocardial infarction	9 (1.7)	6 (1.1)	1.50 (0.53-4.21)	0.44
Death / myocardial infarction	12 (2.3)	10 (1.9)	1.20 (0.52-2.78)	0.67
Target vessel myocardial infarction	8 (1.5)	5 (0.9)	1.60 (0.52-4.89)	0.41
Cerebrovascular accident	0 (0)	4 (0.8)	0.02 (0.00-47.17)	0.31
Target-lesion revascularization	11 (2.1)	18 (3.5)	0.60 (0.29-1.28)	0.19
Target-vessel revascularization	16 (3.0)	20 (3.8)	0.80 (0.41-1.54)	0.50
Any revascularization	30 (5.7)	35 (6.7)	0.85 (0.52-1.39)	0.51
Stent thrombosis	3 (0.6)	1 (0.2)	3.00 (0.31-28.84)	0.34
Any bleeding	3 (0.6)	9 (1.7)	0.33 (0.09-1.22)	0.10
TIMI major bleeding‡	2 (0.4)	3 (0.6)	0.66 (0.11-3.97)	0.65
MACCE§	39 (7.4)	47 (8.9)	0.82 (0.54-1.26)	0.37
Safety endpoint¶	16 (3.0)	16 (3.0)	1.00 (0.50-2.00)	0.99

Data are n (%). The percentages shown are Kaplan–Meier estimates from the intention-to-treat analysis. DAPT indicates dual antiplatelet therapy.

*Hazard ratios (HR) are for the 6-month dual antiplatelet therapy group as compared with the 12-month dual antiplatelet therapy group.

†Target vessel failure was a composite of cardiac death, myocardial infarction, or target vessel revascularization.

‡Thrombolysis in Myocardial Infarction (TIMI) major bleeding refers to adjudicated events in accordance with previously used TIMI criteria.[14]

§Major adverse cardiocerebral event (MACCE) was a composite of death, myocardial infarction, stroke, or any revascularization.

¶Safety endpoint was a composite of death, myocardial infarction, stroke, stent thrombosis, or TIMI major bleeding.

Supplemental Table 6. Clinical outcomes among patients receiving sirolimus-eluting stents

	6-month DAPT (n=182)	12-month DAPT (n=182)	HR (95% CI)†	*P*
Target-vessel failure†	9 (5.1)	4 (2.3)	2.31 (0.71-7.51)	0.16
Total death	1 (0.6)	3 (1.7)	0.34 (0.04-3.25)	0.35
Cardiac death	1 (0.6)	1 (0.6)	1.01 (0.06-16.13)	0.99
Myocardial infarction	4 (2.3)	1 (0.6)	4.07 (0.45-36.37)	0.21
Death / myocardial infarction	5 (2.8)	4 (2.2)	1.27 (0.34-4.74)	0.72
Target vessel myocardial infarction	4 (2.3)	1 (0.6)	4.07 (0.45-36.37)	0.21
Cerebrovascular accident	3 (1.7)	1 (0.6)	3.03 (0.32-29.09)	0.34
Target-lesion revascularization	6 (3.5)	0 (0)	-	0.21
Target-vessel revascularization	6 (3.5)	2 (1.2)	3.09 (0.62-15.31)	0.17
Any revascularization	13 (7.6)	8 (4.6)	1.68 (0.70-4.06)	0.25
Stent thrombosis	3 (1.7)	0 (0)	-	0.38
Any bleeding	1 (0.6)	1 (0.6)	1.02 (0.06-16.26)	0.99
TIMI major bleeding‡	0 (0)	1 (0.6)	-	0.61
MACCE§	17 (9.8)	13 (7.3)	1.35 (0.66-2.79)	0.41
Safety endpoint¶	8 (4.5)	5 (2.8)	1.64 (0.54-5.00)	0.39

Data are n (%). The percentages shown are Kaplan–Meier estimates from the intention-to-treat analysis. DAPT indicates dual antiplatelet therapy.

*Hazard ratios (HR) are for the 6-month dual antiplatelet therapy group as compared with the 12-month dual antiplatelet therapy group.

†Target vessel failure was a composite of cardiac death, myocardial infarction, or target vessel revascularization.

‡Thrombolysis in Myocardial Infarction (TIMI) major bleeding refers to adjudicated events in accordance with previously used TIMI criteria.[14]

§Major adverse cardiocerebral event (MACCE) was a composite of death, myocardial infarction, stroke, or any revascularization.

¶Safety endpoint was a composite of death, myocardial infarction, stroke, stent thrombosis, or TIMI major bleeding.

Supplemental Figure 1

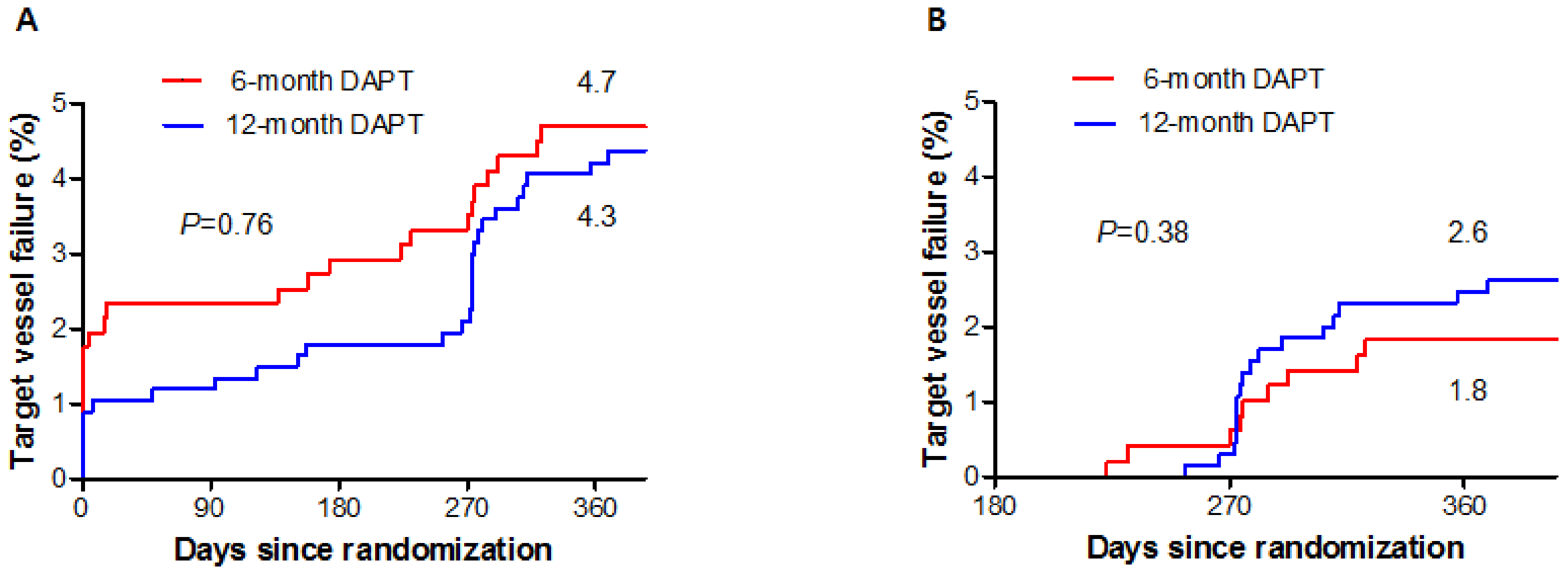

6-month DAPT	514	500	494	491	480
12-month DAPT	672	659	652	647	633

6-month DAPT	494	491	480
12-month DAPT	652	647	633

Supplemental Figure 2

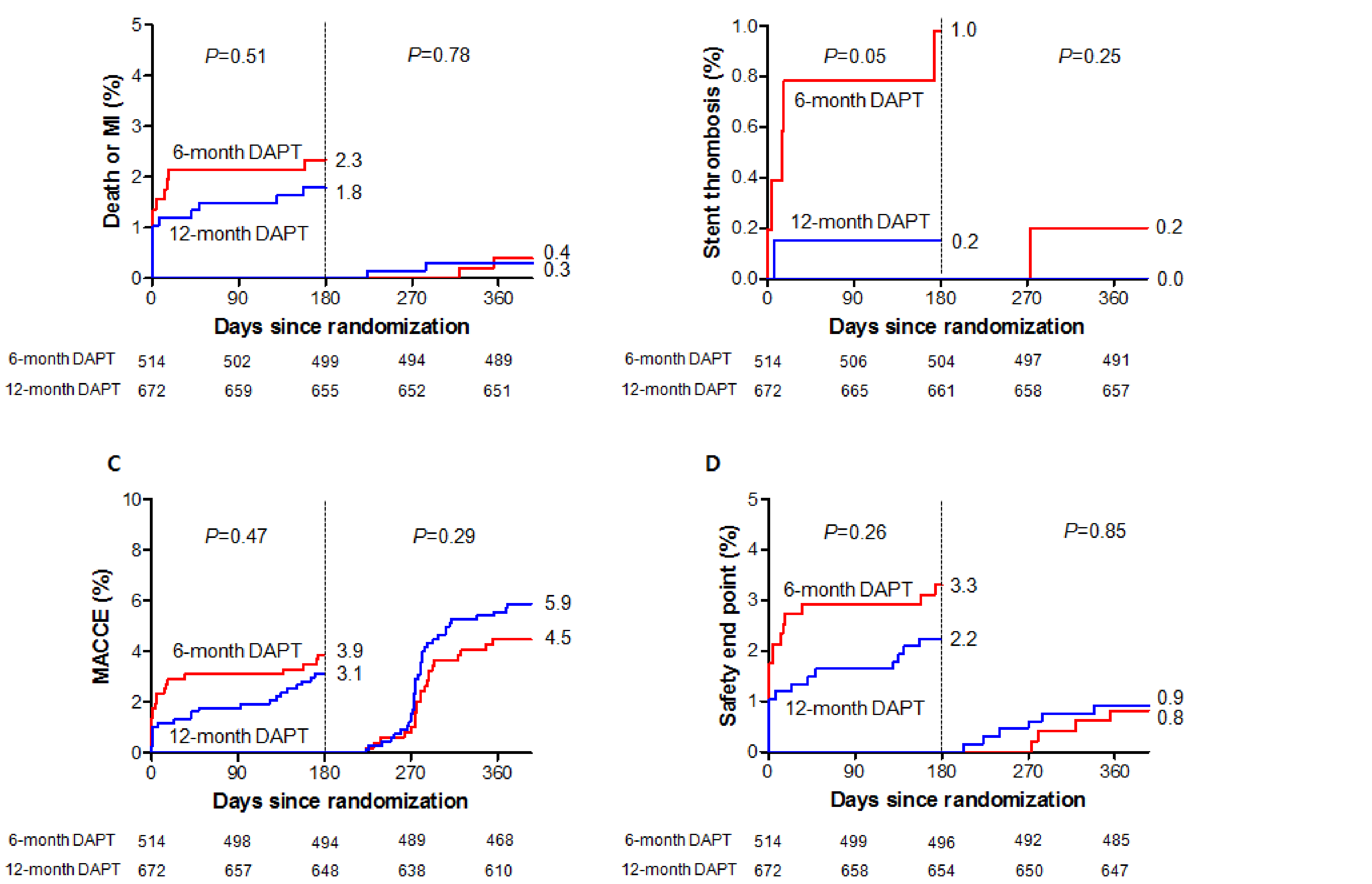

Supplemental Figure 3

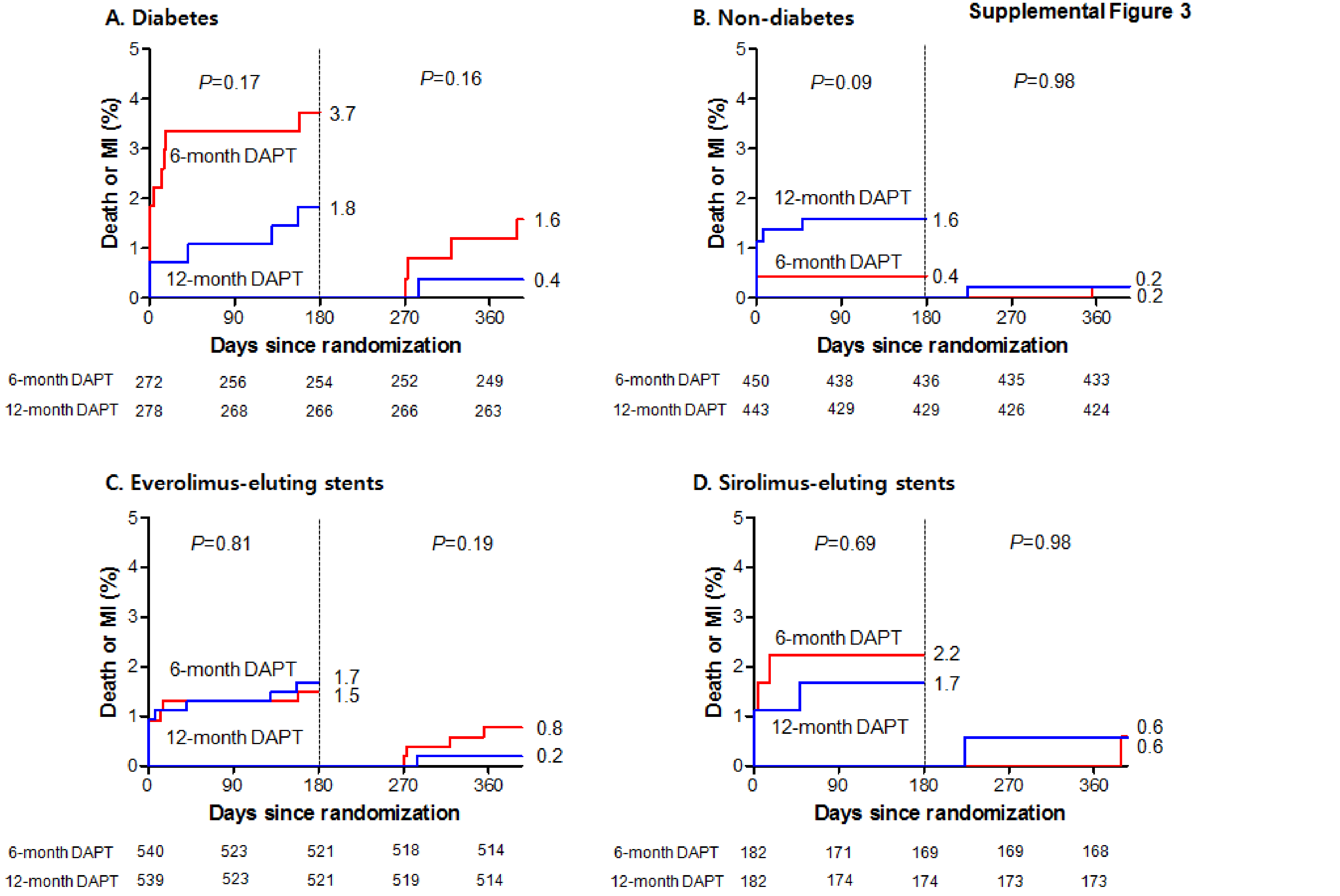

Supplemental Figure Legends

Supplemental Figure 1. Kaplan-Meier curves for the primary end point of target-vessel failure by per-protocol analysis

P values were calculated with the use of the log-rank test.

(A) A composite of cardiac death, myocardial infarction, or target vessel revascularization. (B) Six-month landmark analysis among patients who were event-free at 6 months.

Supplemental Figure 2. Six-month landmark analysis for the key secondary end points by per-protocol analysis

P values were calculated with the use of the log-rank test.

(A) A composite of death or myocardial infarction (MI). (B) Stent thrombosis. (C) Major adverse cardiocerebral event (MACCE: a composite of death, myocardial infarction, stroke, or any revascularization). (D) Safety end point (a composite of death, myocardial infarction, stroke, stent thrombosis, or TIMI major bleeding).

Supplemental Figure 3. Six-month landmark analysis for death or myocardial infarction (MI) according to diabetic status or stent types

P values were calculated with the use of the log-rank test.

(A) A composite of death or MI in patients with diabetes. (B) A composite of death or MI in patients without diabetes. (C) A composite of death or MI in patients receiving everolimus-eluting stents. (D) A composite of death or MI in patients receiving sirolimus-eluting stents.

약물용출스텐트 삽입 후 항혈소판제 2제 요법을 6개월간만 사용하여도 12개월 사용하는 것보다 나쁘지 않은 것 같다

나 승 운 교수 고려대학교 구로병원 순환기내과

Summary

배경

약물용출스텐트를 삽입하고 나서 항혈소판제 2제 요법(dual antiplatelet therapy)의 이상적인 사용기간에 대해서는 아직도 확실하지가 않다. 이 연구의 목적은 약물용출스텐트 삽입 후 항혈소판제 2제 요법을 6개월 사용하는 것이 12개월 사용하는 것보다 열등하지 않은지를 살펴보려는 것이다.

방법 및 결과

이 연구에서 약물용출스텐트를 삽입받은 1,443명의 환자를 1:1의 비율로 항혈소판제 2제를 6개월간 또는 12개월간 투여받는 군으로 무작위 배정하였다. 1차 연구 종말점은 심인성 사망, 심근경색증 또는 심근허혈과 관련된 목표혈관 재시술(ischemia-driven target vessel revascularization)을 포함한 목표혈관실패(target vessel failure)로 삼았다.

목표혈관실패는 2제 요법 6개월 군에서는 4.8%, 그리고 12개월 군에서는 4.3%[일측 95% CI의 상한선, 2.4%; 미리 정의된 비열등성 여지(margin)의 4.0%로 비열등성, *P*=0.001]를 보였다. 비록 스텐트 혈전증은 2제 요법 6개월 군에서 12개월 군보다 더 자주 발생하는 경향을 보였으나(0.9% vs. 0.1%, HR, 6.02; 95% CI, 0.72-49.96; *P*=0.10), 사망 또는 심근경색증의 위험은 두 군 간에 차이가 없었다(2.4% vs. 1.9%, HR, 1.21; 95% CI, 0.60-2.47; *P*=0.58). 좀 더 자세한 하위군 분석에서 목표혈관실패는 당뇨병 환자들에서 2제 요법 12개월 군보다 6개월 군에서 더 자주 발생하였다(HR, 3.16; 95% CI, 1.42-7.03; *P*=0.005).

결론

약물용출스텐트 삽입 후 항혈소판제 2제 투여를 6개월 시행한 군은 12개월 투여군에 비해 목표혈관실패율을 더 증가시키지 않았다. 그러나 비열등성 여지가 넓고(wide non-inferiority margin), 이 연구는 사망이나 심근경색증의 발생빈도의 차이를 보기에는 환자 등록 수가 충분히 많지가 않아(underpowered), 이러한 연구 결과들은 추후 더 큰 임상연구를 통해 확인되어야 할 것으로 보인다.

Commentary

그동안 소위 가이드라인에서는 약물용출스텐트 삽입 후 항혈소판제 2제 요법을 최소한 1년 이상 사용할 것을 권고해 왔으나, 이는 이미 여러 해가 지난 data에 근거해 이루어졌고, 주로 초기 1세대 약물용출스텐트 관련 data를 근거로 정해졌다.[1] 그러나 실제로 이를 뒷받침하는 무작위 배정연구는 없었고, 대부분 관찰연구, 후향적 연구를 토대로 정해졌다. 그런 의미에서 이번에 발표된 EXCELLENT(The Efficacy of Xience/Promus Versus Cypher to Reduce Late Loss After Stenting) trial은 6개월과 12개월 요법을 head-to-head로 비교한 제대로 된 무작위 연구라 할 수 있고, 우리로서는 한국인을 대상으로 한 증거이므로 임상에의 적용에 더 큰 가치를 주는 연구라 할 수 있겠다.

이번 연구의 핵심은 항혈소판제 2제 요법을 약물용출스텐트 삽입 후 6개월만 사용하고, 그 이후에는 aspirin 단독으로만 유지해도 2제 요법을 1년 유지한 군에 비해 목표혈관실패율에 있어서 차이가 없다는 것이다.

항혈소판 2제 요법은 유효성을 유지하며 안전성을 담보하는 최소한의 투여기간이 매우 중요하다 할 수 있는데, 이는 2제 요법과 관련된 여러 가지 출혈성 합병증의 위험도 줄일 수 있고, 장기간 투여에 따른 비용도 줄일 수 있으며, 여러 가지 예기치 않았던 시술 및 수술과 관련된 복잡한 문제들이 좀 더 간단해질 수 있는 장점이 있기 때문이다.

이 연구에서 스텐트 혈전증의 발생이 6개월 투여군에서 많은 경향을 보였으나 유의하지 않았고, 이와 관련하여 사망이나 심근경색증의 발생 위험이 더 증가하지는 않았다. 스텐트 혈전증을 더 자세히 들여다보면, 6개월 투여군에서 발생한 6명의 스텐트 혈전증에서 5명이 그들이 2제 약물을 투여받는 중인 6개월 이내에 발생하여, 실제로 2제 요법이 중단된 6개월 이후에 발생한 경우는 1명만 해당되었다.

그러나 하부군 분석에서 비교적 전신 염증반응과 혈전 발생의 위험, 시술의 재발 위험이 큰 고위험군에 해당하는 당뇨병 환자들에서는 6개월 군이 12개월 군보다 목표혈관실패율이 더 높았고, 스텐트 혈전증도 당뇨병 환자에서는 6개월 투여군에서 4명(1.5%)인 반면에 12개월 투여군에서는 한 명도 발생하지 않았다. 따라서 당뇨병과 같은 고위험 환자들에게는 그래도 12개월 투여가 더 안전할 것으로 생각된다.

이 연구에서 또 한 가지 재미있는 결과는 2제 요법 6개월 군이 12개월 투여군에 비해 sirolimus-eluting stent(SES)를 삽입한 군에서 목표혈관실패율이 높은 경향을 보인 것이다. 그러나 이러한 소견은 everolimus-eluting stent(EES)를 삽입한 군에서는 관찰되지 않았다. 이는 아무래도 1세대 약물용출스텐트인 SES를 삽입하고 6개월만 약제를 쓰기에는 부담을 가질 수밖에 없는 소견으로 보이고, 반면에 2세대 이후의 약물용출스텐트는 6개월 투여군에서도 비교적 안정적인 결과를 보여준다고 해석할 수 있겠다.

그러나 이 연구의 제한점으로, 연구의 목표 피험자 수 산출을 위해 사용한 과거 연구들에 비해 임상사건의 발생률이 낮아 두 군 간의 비교 시 차이를 보기에 상대적으로 어려울 수 있었다는 점,[2,3] 위약-대조 무작위 배정이 아니고 open-label의 무작위 연구라 완전한 개연성을 보장하기 어려운 점이 있었으며, 또한 일정 시점에 내원하는 모든 시술 환자들을 대상으로 하지 않고, 3일 이내의 급성 심근경색증 환자, 좌주간지 병변 또는 심한 좌심실 기능부전 등 위험요소가 높은 환자들은 연구에서 제외되었기 때문에 이러한 고위험 환자들에서 이 결과의 해석은 주의를 필요로 한다고 볼 수 있겠다. 또한, 이는 한국인만을 대상으로 하여 혈전 지혈 관련 경향이나 체질량지수 등에 차이가 있는 서구인들에게도 동일하게 적용할 수 있을지는 추가적인 연구들이 필요하리

라 생각된다.

결론적으로, 이 연구결과를 기초로 생각해 볼 때 한국인에서 당뇨병을 포함한 고위험군의 환자이며, 1세대 약물용출스텐트를 삽입 받은 환자들을 제외한 일반적인 환자들이면서 2세대 이후의 약물용출스텐트를 삽입 받은 환자들은 2제 항혈소판제 투여를 6개월간 유지하고 그 이후에 aspirin 단독 요법으로 유지하는 것이 비용-효과적일 수 있다고 사료된다.

References

1. King SB 3rd, Smith SC Jr, Hirshfeld JW Jr, Jacobs AK, Morrison DA, Williams DO, Feldman TE, Kern MJ, O'Neil WW, Schaff HV, Whitlow PL, Adams CD, Anderson JL, Buller CE, Creager MA, Ettinger SM, Halperin JL, Hunt SA, Krumholz HM, Kushner FG, Lytle BW, Nishimura R, Page RL, Riegel B, Tarkington LG, Yancy CW. 2007 Focused update of the ACC/AHA/SCAI 2005 guideline update for percutaneous coronary intervention: a report of the American College of Cardiology/American Heart Association Task Force on Practice Guidelines: 2007 Writing Group to Review New Evidence and Update the ACC/AHA/SCAI 2005 Guideline Update for Percutaneous Coronary Intervention, writing on behalf of the 2005 writing committee. *Circulation.* 2008;117:261-295.
2. Windecker S, Remondino A, Eberli FR, Jüni P, Räber L, Wenaweser P, Togni M, Billinger M, Tüller D, Seiler C, Roffi M, Corti R, Sütsch G, Maier W, Lüscher T, Hess OM, Egger M, Meier B. Sirolimus-eluting and paclitaxel-eluting stents for coronary revascularization. *N Engl J Med.* 2005;353:653-662.
3. Morice MC, Colombo A, Meier B, Serruys P, Tamburino C, Guagliumi G, Sousa E, Stoll HP; REALITY trial investigators. Sirolimus- vs. paclitaxel-eluting stents in de novo coronary artery lesions: the REALITY trial: a randomized controlled trial. *JAMA.* 2006;295:895-904.

Six-Month Versus 12-Month Dual Antiplatelet Therapy After Implantation of Drug-Eluting Stents

The Efficacy of Xience/Promus Versus Cypher to Reduce Late Loss After Stenting (EXCELLENT) Randomized, Multicenter Study

Hyeon-Cheol Gwon, MD*; Joo-Yong Hahn, MD*; Kyung Woo Park, MD; Young Bin Song, MD; In-Ho Chae, MD; Do-Sun Lim, MD; Kyoo-Rok Han, MD; Jin-Ho Choi, MD; Seung-Hyuk Choi, MD; Hyun-Jae Kang, MD; Bon-Kwon Koo, MD; Taehoon Ahn, MD; Jung-Han Yoon, MD; Myung-Ho Jeong, MD; Taek-Jong Hong, MD; Woo-Young Chung, MD; Young-Jin Choi, MD; Seung-Ho Hur, MD; Hyuck-Moon Kwon, MD; Dong-Woon Jeon, MD; Byung-Ok Kim, MD; Si-Hoon Park, MD; Nam-Ho Lee, MD; Hui-Kyung Jeon, MD; Yangsoo Jang, MD; Hyo-Soo Kim, MD

***Background*—**The optimal duration of dual antiplatelet therapy (DAPT) after implantation of drug-eluting coronary stents remains undetermined. We aimed to test whether 6-month DAPT would be noninferior to 12-month DAPT after implantation of drug-eluting stents.

***Methods and Results*—**We randomly assigned 1443 patients undergoing implantation of drug-eluting stents to receive 6- or 12-month DAPT (in a 1:1 ratio). The primary end point was a target vessel failure, defined as the composite of cardiac death, myocardial infarction, or ischemia-driven target vessel revascularization at 12 months. Rates of target vessel failure at 12 months were 4.8% in the 6-month DAPT group and 4.3% in the 12-month DAPT group (the upper limit of 1-sided 95% confidence interval, 2.4%; $P=0.001$ for noninferiority with a predefined noninferiority margin of 4.0%). Although stent thrombosis tended to occur more frequently in the 6-month DAPT group than in the 12-month group (0.9% versus 0.1%; hazard ratio, 6.02; 95% confidence interval, 0.72–49.96; $P=0.10$), the risk of death or myocardial infarction did not differ in the 2 groups (2.4% versus 1.9%; hazard ratio, 1.21; 95% confidence interval, 0.60–2.47; $P=0.58$). In the prespecified subgroup analysis, target vessel failure occurred more frequently in the 6-month DAPT group than in the 12-month group (hazard ratio, 3.16; 95% confidence interval, 1.42–7.03; $P=0.005$) among diabetic patients.

***Conclusions*—**Six-month DAPT did not increase the risk of target vessel failure at 12 months after implantation of drug-eluting stents compared with 12-month DAPT. However, the noninferiority margin was wide, and the study was underpowered for death or myocardial infarction. Our results need to be confirmed in larger trials.

***Clinical Trial Registration*—**URL: http://www.clinicaltrials.gov. Unique identifier: NCT00698607.

(*Circulation*. 2012;125:505-513.)

Key Words: drug-eluting stents ■ platelet aggregation inhibitors ■ stents ■ thrombosis

Several randomized trials have demonstrated that drug-eluting coronary stents reduce angiographic restenosis and target lesion revascularization compared with bare metal stents.[1–3] However, some long-term observational studies have reported that the risk of death or myocardial infarction was higher after drug-eluting stents than after bare metal stents, which may be due to the different incidences of late or very late stent thrombosis.[4,5] Previous observational studies

Received July 29, 2011; accepted November 8, 2011.

From the Division of Cardiology, Department of Medicine, Samsung Medical Center, Sungkyunkwan University School of Medicine, Seoul (H.-C.G., J.-Y.H., Y.B.S., J.-H.C., S.-H.C.); Cardiovascular Center, Seoul National University Main Hospital, Seoul (K.W.P., H.-J.K., B.-K.K., H.-S.K.); Seoul National University Bundang Hospital, Sungnam (I.-H.C.); Korea University Anam Hospital, Seoul (D.-S.L.); Kangdong Sacred Heart Hospital, Seoul (K.-R.H.); Gachon University Gil Medical Center, Incheon (T.A.); Yonsei University Wonju Severance Hospital, Wonju (J.-H.Y.); Chonnam National University Hospital, Gwangju (M.-H.J.); Busan National University Hospital, Busan (T.-J.H.); Seoul National University Boramae Hospital, Seoul (W.-Y.C.); Hallym University Sacred Heart Hospital, Anyang (Y.-J.C.); Keimyung University Dongsan Hospital, Daegu (S.-H.H.); Gangnam Severance Hospital, Seoul (H.-M.K.); NHIC Ilsan Hospital, Goyang (D.-W.J.); Inje University Sanggye Paik Hospital, Seoul (B.-O.K.); Ewha Women's University Mokdong Hospital, Seoul (S.-H.P.); Kangnam Sacred Heart Hospital, Seoul (N.-H.L.); Catholic University Uijeongbu St. Mary's Hospital, Uijeongbu (H.-K.J.); and Yonsei University Severance Hospital, Seoul (Y.J.), Korea.

*Drs Gwon and Hahn contributed equally to this article.

The online-only Data Supplement is available with this article at http://circ.ahajournals.org/lookup/suppl/doi:10.1161/CIRCULATIONAHA.111.059022/-/DC1.

Correspondence to Hyo-Soo Kim, MD, Department of Internal Medicine, Cardiovascular Center, Seoul National University Hospital, 101 DaeHak-ro, JongRo-gu, Seoul, 110–744, Korea. E-mail hyosoo@snu.ac.kr

© 2011 American Heart Association, Inc.

***Circulation* is available at http://circ.ahajournals.org** **DOI: 10.1161/CIRCULATIONAHA.111.059022**

reported that premature discontinuation of thienopyridine therapy was the major determinant of stent thrombosis after implantation of drug-eluting stents[6] and that the extended use of clopidogrel in patients with drug-eluting stents may be associated with a reduced risk of death or myocardial infarction.[7] From the results of these reports, prolonged dual antiplatelet therapy (DAPT; aspirin plus thienopyridine) of at least 12 months is currently recommended after percutaneous coronary intervention (PCI) with drug-eluting stents unless patients are at high risk for bleeding.[8]

Clinical Perspective on p 90

However, the optimal or minimal necessary duration of DAPT remains undetermined. A randomized trial showed that the use of DAPT for a period >12 months in patients who had received drug-eluting stents was not significantly more effective than aspirin monotherapy.[9] Moreover, some registry studies suggest that DAPT lasting <12 months after PCI with drug-eluting stents does not increase major adverse cardiac events and that there is no apparent clinical benefit from DAPT for >6 months.[10–12] To date, no randomized trials have been performed to compare a shorter duration of DAPT with 12-month DAPT. In the Efficacy of Xience/Promus Versus Cypher to Reduce Late Loss After Stenting (EXCELLENT) trial, we compared 6-month DAPT with 12-month DAPT in patients receiving drug-eluting stents.

Methods

Study Design and Patients

The EXCELLENT trial was a prospective, open-label, randomized trial conducted at 19 sites in Korea. The authors designed the study, and the institutional review board at each participating center approved the trial protocol. The study design has previously been described.[13] Patients were eligible for inclusion in the study if they had at least 1 lesion in a native coronary vessel with a reference diameter of 2.25 to 4.25 mm, stenosis of >50% by visual estimation, and evidence of myocardial ischemia such as stable angina, unstable angina, recent myocardial infarction, silent ischemia, a positive functional study, or reversible changes on ECG consistent with ischemia. Documentation of ischemia was not mandatory for lesions with >75% stenosis. There were no limitations on the number of lesions or the length of the lesions in efforts to reflect real-life clinical practice. Exclusion criteria were myocardial infarction within 72 hours; severely compromised ventricular dysfunction (ejection fraction <25%) or cardiogenic shock; any stent implantation in the target vessel before enrollment; hemoglobin <10 g/dL or platelet count <100 000 per 1 μL; serum creatinine ≥265.2 μmol/L (3.0 mg/dL) or dependence on dialysis; serious hepatic disease; major bleeding within 3 months or major surgery within 2 months; allergy to antiplatelet drugs, heparin, stainless steel, contrast agents, everolimus, or sirolimus; elective surgical procedure planned within <12 months; life expectancy <1 year; significant left main disease defined as stenosis of >50%; chronic total occlusion; true bifurcation lesions requiring a planned 2-stent strategy; or active participation in another clinical study. All patients provided written informed consent.

Study Procedures and Follow-Up

Patients were randomly assigned in a 1:1 ratio to receive either 6-month DAPT (aspirin 100–200 mg/d plus clopidogrel 75 mg/d for 6 months and thereafter aspirin alone) or 12-month DAPT (aspirin 100–200 mg/d plus clopidogrel 75 mg/d for 12 months). Randomization was performed with a Web-based response system after diagnostic angiography and before PCI. Randomization was stratified by the site of enrollment, presence of diabetes mellitus, and lesion length. In addition, patients were randomly assigned to receive everolimus- or sirolimus-eluting stents. The results of the drug-eluting stent arm of the trial are not reported here.

PCI was performed according to standard techniques. Before the index procedure, all patients received at least 300 mg aspirin and a 300- to 600-mg loading dose of clopidogrel unless they had previously received these antiplatelet medications. Unfractionated heparin was administered throughout the procedure to maintain an activated clotting time of ≥250 seconds. Administration of glycoprotein IIb/IIIa inhibitors was at the discretion of the operator. After the procedure, all patients were recommended to receive optimal pharmacological therapy, including statins, β-blockers, or angiotensin-converting enzyme inhibitors at the discretion of the responsible clinicians. Any P2Y12 receptor antagonist other than clopidogrel was not used. Additionally, each investigator was advised to emphasize the importance of cardiovascular risk factor modification to patients.

Clinical follow-up was performed at 1, 3, 6, 9, and 12 months after the index PCI. At follow-up, patient data, including clinical status, all interventions, outcome events, and adverse events, were recorded. In particular, information on the use of aspirin or clopidogrel was assessed at each follow-up.

Study End Points

The primary end point was target vessel failure defined as a composite of cardiac death, myocardial infarction, or target vessel revascularization during the 12-month period after randomization. Secondary end points included the individual components of the primary end point; death resulting from any cause; death or myocardial infarction; stent thrombosis; major bleeding according to the Thrombolysis in Myocardial Infarction criteria[14]; major adverse cardiocerebral events, which were a composite of death, myocardial infarction, stroke, or any revascularization; and a safety end point, which was a composite of death, myocardial infarction, stroke, stent thrombosis, or Thrombolysis in Myocardial Infarction major bleeding.

Clinical events were defined on the basis of the recommendations of the Academic Research Consortium.[15] All deaths were considered cardiac unless a definite noncardiac cause could be established. During the first 48 hours after PCI, myocardial infarction was defined as an increase of cardiac enzyme (creatine kinase-MB fraction or troponin T/troponin I) 3 times above the upper limit of normal in stable patients.[15] In patients with elevated baseline levels of cardiac enzyme, myocardial infarction was defined as a subsequent increase of >2-fold from baseline values.[16] After the first 48 hours, myocardial infarction was defined as the presence of clinical signs of myocardial infarction combined with a creatine kinase-MB fraction or troponin T/troponin I increase higher than the upper limit of normal.[15] Target lesion revascularization was defined as either a repeat PCI of the lesion within 5 mm of the deployed stent or bypass graft surgery of the target vessel. Target vessel revascularization was defined as repeat revascularization of the treated vessel by PCI or bypass graft surgery. Stent thrombosis was defined as definite or probable stent thrombosis according to the Academic Research Consortium classification.[15] Stroke, as detected by the occurrence of a new neurological deficit, was confirmed by a neurologist and on imaging. Device success was defined as the attainment at the target site of a final residual diameter stenosis of <50% using only the assigned study device. Lesion success was defined as the attainment of a final residual diameter stenosis of <50% using any percutaneous method. Procedure success was defined as the attainment at the target site of a final residual diameter stenosis of <50%, together with the absence of any in-hospital major adverse cardiac events. The independent clinical event adjudication committee (Table I in the online-only Data Supplement), the members of which were unaware of the study group assignments, assessed all of the clinical end points.

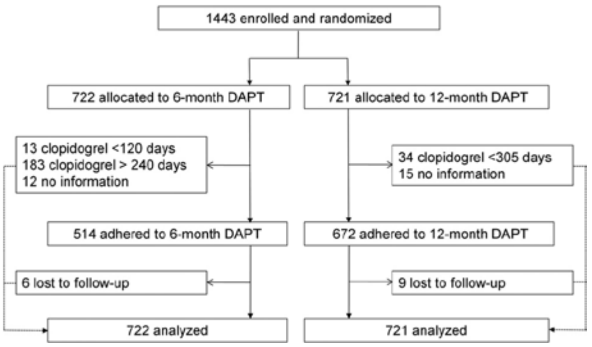

Figure 1. Trial profile. DAPT indicates dual antiplatelet therapy.

Statistical Analysis

The primary analysis was a noninferiority comparison of 6- and 12-month DAPT for the primary end point of target vessel failure according to the intention-to-treat principle. Using data from large, randomized clinical trials evaluating the efficacy of drug-eluting stents, we estimated that the incidence of the primary end point with 12-month DAPT 1 year after the procedure would be 10%.[17,18] The noninferiority margin of 4.0 percentage points was chosen on the basis of historical data,[7] clinically acceptable relevance, and the feasibility of study recruitment. We estimated that with a total of 1372 patients (686 per group), the power of the study would be 80% to show noninferiority with a 1-sided type I error rate of 0.05. Assuming that 5% of patients would be lost to follow-up, we determined the final sample size to be 1440 patients (720 per group).

Continuous variables were presented as mean±SD and compared by use of the Student *t* test. Categorical variables were presented as counts and percentages and compared by use of the χ^2 or Fisher exact test as appropriate. Cumulative event rates were estimated with the Kaplan-Meier method. If the upper limit of the 1-sided 95% confidence interval (CI) of the difference were less than the prespecified noninferiority margin, 6-month DAPT would be considered to be noninferior to 12-month DAPT. Survival curves were compared by use of the log-rank tests. Hazard ratios with 95% CIs were estimated by use of the Cox proportional-hazards method. Landmark analysis[19] was performed with a landmark of clopidogrel discontinuation at 6 months among patients who were event free at 6 months. We also performed per-protocol analysis among patients who adhered to the study protocol. The consistency of treatment effects in prespecified subgroups was assessed by use of Cox regression models with tests for interaction. *P* values and CIs were 2 tailed except those for noninferiority testing of the primary end point. All analyses were performed with SAS version 9.1 (SAS Institute, Inc, Cary, NC).

The authors had full access to and take full responsibility for the integrity of the data. All authors have read and agree to the manuscript as written.

Results

Study Participants

Between June 2008 and July 2009, a total of 1443 patients were enrolled. Of these patients, 722 were assigned to receive 6-month DAPT and 721 were assigned to receive 12-month DAPT (Figure 1). Patients in the 2 groups were well balanced with regard to most baseline demographic and clinical characteristics (Table 1). However, patients with a history of previous myocardial infarction were more common in the 6-month DAPT group compared with the 12-month group (6.5% versus 3.7%; $P=0.02$). Medications at discharge from the index PCI were similar in the 6- and 12-month DAPT groups. Angiographic and procedural data were also similar in the 2 groups (Table 2). Everolimus-eluting stents were used in three quarters of patients and sirolimus-eluting stents were used in one quarter of patients as a result of 3:1 randomization of stents.

Table 1. Baseline Patient Characteristics

	6-mo DAPT (n=722)	12-mo DAPT (n=721)	*P*
Age, y	63.0±9.6	62.4±10.4	0.21
Male sex, n (%)	470 (65.1)	461 (63.9)	0.65
Body mass index, kg/m^2	24.9±3.1	25.1±3.0	0.32
Diabetes mellitus, n (%)	272 (37.7)	278 (38.6)	0.73
Hypertension, n (%)	525 (72.7)	532 (73.8)	0.65
Dyslipidemia, n (%)	543 (75.2)	550 (76.3)	0.63
Current smoker, n (%)	198 (27.4)	186 (25.8)	0.49
Previous myocardial infarction, n (%)	47 (6.5)	27 (3.7)	0.02
Previous PCI, n (%)	67 (9.3)	62 (8.6)	0.65
Previous CABG, n (%)	11 (1.5)	7 (1.0)	0.34
Congestive heart failure, n (%)	4 (0.6)	5 (0.7)	0.75
Chronic renal failure, n (%)	6 (0.8)	9 (1.2)	0.44
Cerebrovascular disease, n (%)	47 (6.5)	48 (6.7)	0.91
Clinical presentation, n (%)			0.56
Silent ischemia/stable angina	353 (48.9)	346 (48.0)	
Unstable angina/non–ST-segment–elevation myocardial infarction	350 (48.5)	349 (48.4)	
ST-elevation myocardial infarction	19 (2.6)	26 (3.6)	
Ejection fraction, %	61.0±9.6	61.6±9.4	0.30
Discharge medications, n (%)			
Aspirin	707 (99.4)	704 (99.0)	0.36
Clopidogrel	702 (98.7)	708 (99.6)	0.08
Statin	604 (85.0)	582 (81.9)	0.12
ACE inhibitor	224 (31.5)	243 (34.2)	0.28
Angiotensin II receptor antagonist	244 (34.3)	231 (32.5)	0.46
β-blocker	427 (60.1)	445 (62.6)	0.33

DAPT indicates dual antiplatelet therapy; PCI, percutaneous coronary intervention; CABG, coronary artery bypass grafting; and ACE, angiotensin-converting enzyme. Data are mean±SD when appropriate. Data are given for the intention-to-treat population.

Study Outcomes

At 12 months, aspirin was continued in 99.9% of the 6-month DAPT group and 99.3% of the 12-month DAPT group. The median duration of DAPT was 190 days (interquartile range, 181–260 days) in the 6-month DAPT group and 375 days (interquartile range, 364–395 days) in the 12-month DAPT group. Adherence to the study protocol was 71.2% of the 6-month DAPT group and 93.2% of the 12-month DAPT group at 12 months.

Follow-up regarding the primary end point was complete in 99.1% of patients in the 6-month DAPT group and 98.8% in the 12-month group. At 12 months, the primary end point of target vessel failure was noted in 34 patients in the 6-month DAPT group and 30 patients in the 12-month group. Cumulative rates of target vessel failure at 1 year were 4.8% for the 6-month and 4.3% for the 12-month DAPT group. The noninferiority of the 6-month DAPT to 12-month DAPT was

Table 2. Lesion and Procedural Characteristics

	6-mo DAPT	12-mo DAPT	*P*
Patients, n	722	721	
Angiographic disease extent, n (%)			0.90
1-Vessel disease	347 (48.1)	346 (48.0)	
2-Vessel disease	226 (31.3)	232 (32.2)	
3-Vessel disease	149 (20.6)	143 (19.8)	
Left anterior descending artery treated, n (%)	452 (63.0)	447 (62.2)	0.73
Use of glycoprotein IIb/IIIa inhibitors, n (%)	12 (1.7)	12 (1.7)	0.99
Use of intravascular ultrasound, n (%)	315 (43.6)	312 (43.3)	0.89
Treated lesions per patient, n	1.3±0.6	1.4±0.5	0.58
Stents per patient, n	1.6±1.0	1.6±0.9	0.39
Type of drug-eluting stents, n (%)			0.99
Everolimus	540 (74.8)	539 (74.8)	
Sirolimus	182 (25.2)	182 (25.2)	
Treated lesions, n	957	970	
Left anterior descending artery, n (%)	482 (50.6)	474 (49.0)	0.51
ACC/AHA lesion class B2/C, n (%)	486 (52.8)	505 (53.8)	0.67
Long lesion (≥20 mm), n (%)	355 (40.3)	374 (41.2)	0.73
Total occlusion, n (%)	39 (4.2)	27 (2.9)	0.11
Thrombotic lesion, n (%)	74 (8.0)	73 (7.8)	0.84
Ulcerative lesion, n (%)	23 (2×4)	16 (1.6)	0.23
Bifurcation lesion, n (%)	98 (10.2)	111 (11.4)	0.42
Stents per lesion, n	1.2±0.5	1.2±0.5	0.41
Stent length per lesion, mm	27.8±13.0	28.3±13.7	0.31
Lesion success, n (%)	941 (99.7)	964 (99.8)	0.64
Device success, n (%)	941 (99.7)	963 (99.7)	0.98
Procedural success, n (%)	935 (99.0)	956 (99.0)	0.66

DAPT indicates dual antiplatelet therapy; ACC, American College of Cardiology; and AHA, American Heart Association. Data are mean±SD when appropriate. Data are given for the intention-to-treat population.

statistically significant (absolute risk difference, 0.5 percentage points; upper limit of 1-sided 95% CI, 2.4%; $P=0.001$ for noninferiority; Table 3 and Figure 2A). Six-month landmark analysis showed that the risk of target vessel failure at 12 months was not significantly higher in the 6-month DAPT group than in the 12-month group (hazard ratio, 1.06; 95% CI, 0.56–2.03; $P=0.85$; Figure 2B). No significant differences were observed between the 2 groups in the secondary end points (Table 3 and Figure 3). Although stent thrombosis tended to occur more frequently in the 6-month DAPT group than in the 12-month group (0.9% versus 0.1%; hazard ratio, 6.02; 95% CI, 0.72–49.96; $P=0.10$), the risk of death or myocardial infarction did not differ between the 2 groups (2.4% versus 1.9%; hazard ratio, 1.21; 95% CI, 0.60–2.47; $P=0.58$). Five of 6 stent thrombosis cases in the 6-month DAPT group occurred before 6 months when patients were taking both aspirin and clopidogrel. In the remaining 1 patient who developed it after 6 months, stent thrombosis occurred 89 days after discontinuation of clopidogrel. In the 12-month DAPT group, there was only 1 case of stent thrombosis, which developed at 7 days after the index procedure (Table 4).

The results from the per-protocol analysis were similar to those from the intention-to-treat analysis. Target vessel failure occurred in 24 of 514 patients in the 6-month DAPT group and 29 of 672 patients in the 12-month DAPT group. Cumulative rates of target vessel failure at 1 year were 4.7% for the 6-month DAPT group and 4.4% for the 12-month DAPT group. The noninferiority of the 6-month DAPT to the 12-month DAPT was also statistically significant (absolute risk difference, 0.3 percentage points; the upper limit of 1-sided 95% CI, 2.3%; $P<0.001$ for noninferiority; Table II and Figure I in the online-only Data Supplement). Although stent thrombosis tended to occur more frequently in the 6-month DAPT group than in 12-month group (1.2% versus 0.2%; hazard ratio, 7.88; 95% CI, 0.95–65.44; $P=0.06$), the risk of death or myocardial infarction did not differ significantly in the 2 groups (2.7% versus 2.1%; hazard ratio, 1.31; 95% CI, 0.63–2.75; $P=0.47$; Table II and Figure II in the online-only Data Supplement).

In prespecified subgroup analysis, the results of comparison between the 2 regimens were consistent across various subgroups (Figure 4). However, there was significant interaction between diabetes mellitus and outcomes (interaction $P<0.001$). Target vessel failure occurred more frequently in the 6-month DAPT group than in the 12-month group among diabetic patients (hazard ratio, 3.16; 95% CI, 1.42–7.03; $P=0.005$), whereas it occurred less frequently in the 6-month DAPT group than in the 12-month group among patients without diabetes mellitus (hazard ratio, 0.44; 95% CI, 0.21–0.94; $P=0.03$). Results of detailed subgroup analysis according to diabetic status are presented in Tables III and IV and Figure IIIA and IIIB in the online-only Data Supplement. Among diabetic patients, rates of myocardial infarction and target vessel revascularization were significantly higher in the 6-month DAPT group than in the 12-month DAPT group (4.5% versus 1.1%; hazard ratio, 4.14; 95% CI, 1.17–14.68; $P=0.03$; and 5.3% versus 1.9%; hazard ratio, 2.91; 95% CI, 1.05–8.08; $P=0.04$, respectively). Stent thrombosis occurred in 4 patients (1.5%) in the 6-month DAPT group compared with none in the 12-month DAPT group among diabetic patients. Although statistical significance was not achieved, the risk of target vessel failure tended to be higher in the 6-month DAPT group than in the 12-month group among patients receiving sirolimus-eluting stents, whereas such a tendency was not observed in those receiving everolimus-eluting stents (P for interaction=0.18). We compared clinical outcomes of 6-month and 12-month DAPT in detail according to the type of stents (Tables V and VI and Figure IIIC and IIID in the online-only Data Supplement). No significant differences were observed in clinical outcomes between the 6- and 12-month DAPT groups among patients receiving everolimus-eluting stents and patients receiving sirolimus-eluting stents.

Discussion

In this prospective, randomized trial, 6-month DAPT was noninferior to 12-month DAPT for the primary end point, the rate of target vessel failure at 12 months. The 6-month landmark analysis and per-protocol analysis showed consistent results. However, target vessel failure occurred more

Table 3. Clinical Outcomes

	6-mo DAPT (n=722), n (%)	12-mo DAPT (n=721), n (%)	HR* (95% CI)	*P*
Target vessel failure†	34 (4.8)	30 (4.3)	1.14 (0.70–1.86)	0.60
Total death	4 (0.6)	7 (1.0)	0.57 (0.17–1.95)	0.37
Cardiac death	2 (0.3)	3 (0.4)	0.67 (0.11–3.99)	0.66
Myocardial infarction	13 (1.8)	7 (1.0)	1.86 (0.74–4.67)	0.19
Death/myocardial infarction	17 (2.4)	14 (1.9)	1.21 (0.60–2.47)	0.58
Target vessel myocardial infarction	12 (1.7)	6 (0.8)	2.00 (0.75–5.34)	0.16
Cerebrovascular accident	3 (0.4)	5 (0.7)	0.60 (0.14–2.51)	0.48
Target lesion revascularization	17 (2.4)	18 (2.6)	0.94 (0.49–1.83)	0.86
Target vessel revascularization	22 (3.1)	22 (3.2)	1.00 (0.56–1.81)	0.99
Any revascularization	43 (6.2)	43 (6.2)	1.00 (0.66–1.53)	0.99
Stent thrombosis	6 (0.9)	1 (0.1)	6.02 (0.72–49.96)	0.10
Any bleeding	4 (0.6)	10 (1.4)	0.40 (0.13–1.27)	0.12
TIMI major bleeding‡	2 (0.3)	4 (0.6)	0.50 (0.09–2.73)	0.42
MACCE§	56 (8.0)	60 (8.5)	0.94 (0.65–1.35)	0.72
Safety end point¶	24 (3.3)	21 (3.0)	1.15 (0.64–2.06)	0.64

DAPT indicates dual antiplatelet therapy; HR, hazard ratio; CI, confidence interval; TIMI, Thrombolysis in Myocardial Infarction; and MACCE, major cardiocerebral event. The percentages shown are Kaplan-Meier estimates from the intention-to-treat analysis.

*HRs are for the 6- versus 12-month DAPT group.

†Target vessel failure was a composite of cardiac death, myocardial infarction, or target vessel revascularization.

‡TIMI major bleeding refers to adjudicated events in accordance with previously used TIMI criteria.[14]

§MACCE was a composite of death, myocardial infarction, stroke, or any revascularization.

¶Safety end point was a composite of death, myocardial infarction, stroke, stent thrombosis, or TIMI major bleeding.

frequently with 6-month DAPT than with 12-month DAPT among diabetic patients.

Prolonged DAPT of at least 12 months is currently recommended after PCI with drug-eluting stents. Supporting this guideline, several observational studies reported that the risk of death or myocardial infarction increased after drug-eluting stents compared with bare metal stents[4,5] and that the extended use of clopidogrel in patients with drug-eluting stents reduced the risk of death or myocardial infarction.[7] However, DAPT increases bleeding risk[20,21] and costs compared with aspirin alone. Endoscopic, dental, and surgical procedures are often delayed because of prolonged DAPT, which may affect the patient's quality of life.[22] Therefore, determining the optimal or minimal necessary duration of DAPT is very important.

Until now, the premature results of only 1 randomized trial have been reported comparing the clinical outcomes of DAPT versus aspirin alone beyond 12 months, and those results did not support the use of DAPT for a period >12 months in patients receiving drug-eluting stents.[9] The study, however, was not a dedicated randomized trial but a mixture of different cohorts limited by the wide range of the duration of antiplatelet therapy at the time of inclusion, and it was underpowered mainly because of lower rate of primary end point than expected. There has been no prospective randomized trial comparing 12 months and shorter durations of DAPT after drug-eluting stent implantation. Several observational studies reported that discontinuation of thienopyridine therapy beyond 6 months after implantation of drug-eluting stents was not associated with an increased risk of stent thrombosis.[10,11] However, these studies were not randomized trials and were limited by selection bias. In other words, there have been no systematic studies to assess the optimal duration of DAPT. Therefore, we performed a prospective, random-

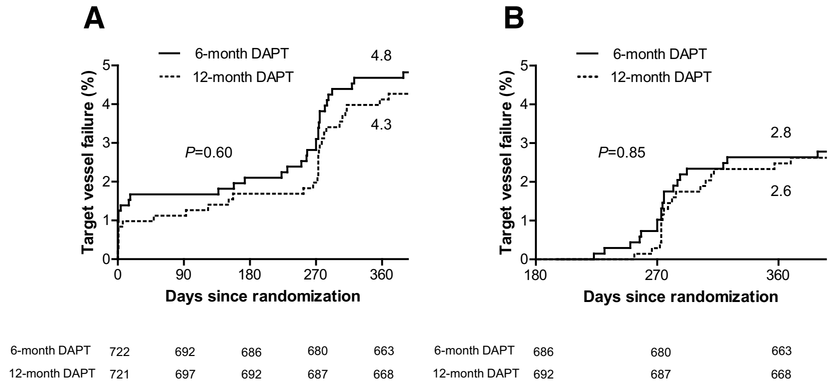

Figure 2. Kaplan-Meier curves for the primary end point of target vessel failure. *P* values were calculated with the log-rank test. **A**, A composite of cardiac death, myocardial infarction, or target vessel revascularization by intention-to-treat analysis. **B**, Six-month landmark analysis among patients who were event-free at 6 months. DAPT indicates dual antiplatelet therapy.

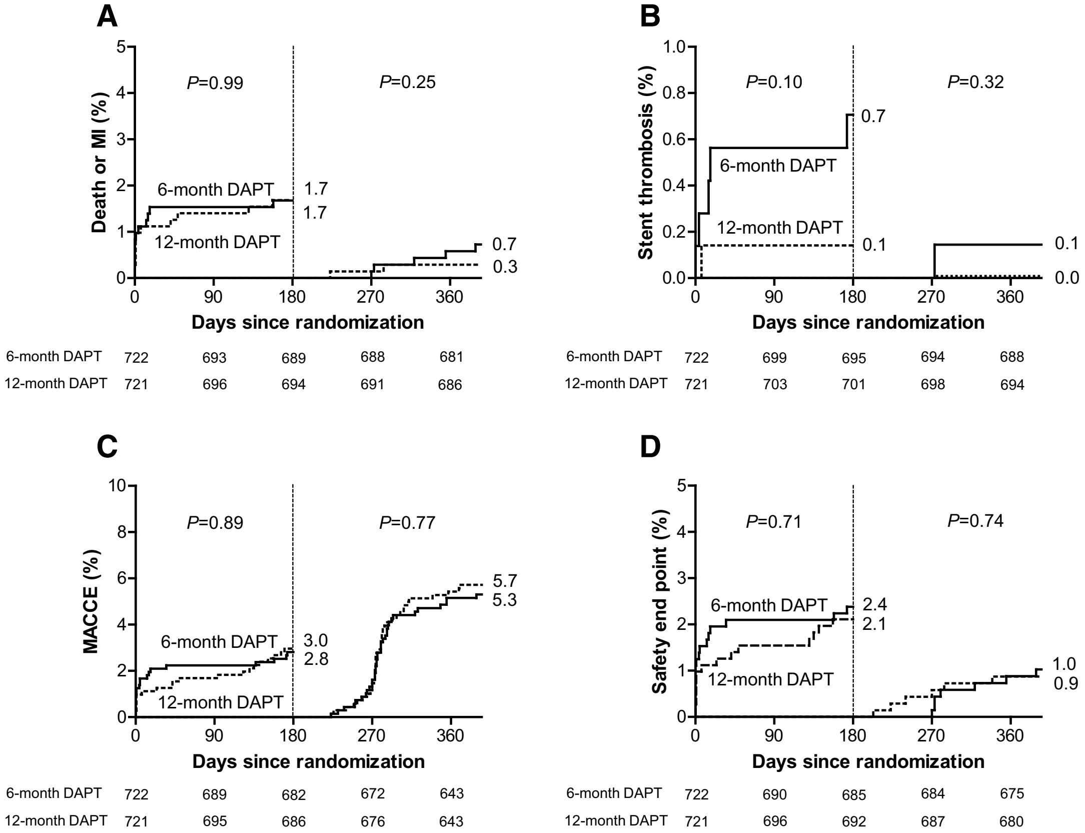

Figure 3. Six-month landmark analysis for the key secondary end points. *P* values were calculated with the log-rank test. **A**, A composite of death or myocardial infarction (MI). **B**, Stent thrombosis. **C**, Major adverse cardiocerebral events (MACCE; a composite of death, MI, stroke, or any revascularization). **D**, Safety end point (a composite of death, MI, stroke, stent thrombosis, or Thrombolysis in Myocardial Infarction major bleeding). DAPT indicates dual antiplatelet therapy.

ized study to compare 6- and 12-month DAPT in patients receiving drug-eluting stents.

The main finding of our study was that the cumulative incidences of target vessel failure did not differ significantly between 6-month and 12-month DAPT. In addition, there were no significant differences in the secondary end points such as death/myocardial infarction, revascularization, or major adverse cardiocerebral events. Our results confirm the results of previous registry data that there were no apparent clinical benefits from DAPT for >6 months.[10–12] However, stent thrombosis tended to occur more frequently in the 6-month DAPT group than in the 12-month group. The power of our study was insufficient to reach conclusions regarding the relationship between stent thrombosis and duration of

Table 4. Detailed Information on Stent Thrombosis

Time to Stent Thrombosis, d	Classification	Group	Clinical Presentation	Diabetic Status	Ejection Fraction, %	Stent Type	Aspirin	Clopidogrel	Outcome
0	Definite	6-mo DAPT	ST-segment–elevation myocardial infarction	No	55	EES	Continued	Continued	TLR
4	Definite	6-mo DAPT	Stable angina	Yes (OHA treated)	58	SES	Continued	Continued	Myocardial infarction
7	Probable	12-mo DAPT	Unstable angina	No	74	EES	Continued	Continued	Death
15	Definite	6-mo DAPT	Non–ST-segment–elevation myocardial infarction	Yes (OHA treated)	62	EES	Continued	Continued	Myocardial infarction
17	Definite	6-mo DAPT	Stable angina	Yes (OHA treated)	70	SES	Continued	Continued	Myocardial infarction
173	Definite	6-mo DAPT	Stable angina	Yes (OHA treated)	Not available	EES	Continued	Continued	TLR
273	Definite	6-mo DAPT	Stable angina	No	70	SES	Continued	Discontinued at day 184	TLR

DAPT indicates dual antiplatelet therapy; EES, everolimus-eluting stent; TLR, target lesion revascularization; OHA, oral hypoglycemic agents; and SES, sirolimus-eluting stent.

	Total	6-mo DAPT n (%)	12-mo DAPT n (%)	HR (95% CI)	*P* value	Interaction *P* value
**Age**						
<65	767	19 (5.1)	12 (3.2)	1.61 (0.78-3.31)	0.20	0.19
≥65	676	15 (4.5)	18 (5.5)	0.83 (0.42-1.65)	0.59	
**ACS**						
No	699	21 (6.03)	13 (3.82)	1.61 (0.8-3.21)	0.18	0.15
Yes	744	13 (3.65)	17 (4.69)	0.78 (0.38-1.60)	0.50	
**Diabetes**						
No	893	10 (2.27)	22 (5.08)	0.44 (0.21-0.94)	0.03	<0.001
Yes	550	24 (9.09)	8 (2.96)	3.16 (1.42-7.03)	0.005	
**LV ejection fraction**						
≥50%	1097	26 (4.91)	24 (4.42)	1.12 (0.64-1.95)	0.69	0.27
<50%	124	2 (3.08)	4 (7.41)	0.41 (0.07-2.23)	0.30	
**Bifurcation**						
No	1243	27 (4.41)	22 (3.65)	1.22 (0.69-2.14)	0.49	0.70
Yes	200	7 (7.61)	8 (7.96)	0.97 (0.35-2.67)	0.95	
**Type of stent**						
EES	1079	25 (4.72)	26 (4.94)	0.96 (0.55-1.66)	0.89	0.18
SES	364	9 (5.14)	4 (2.27)	2.31 (0.71-7.5)	0.16	
**Multi-stent**						
No	866	14 (3.18)	12 (2.91)	1.1 (0.51-2.38)	0.81	0.80
Yes	567	20 (7.68)	18 (6.2)	1.25 (0.66-2.37)	0.49	

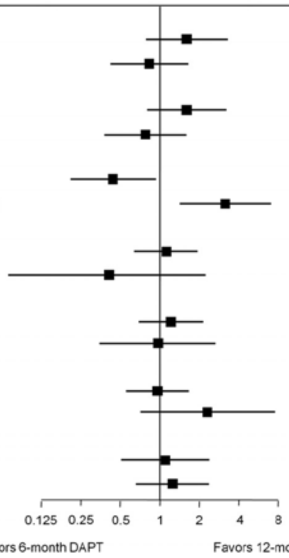

Figure 4. Subgroup analyses of the primary end point. DAPT indicates dual antiplatelet therapy; HR, hazard ratio; CI, confidence interval; ACS, acute coronary syndrome; LV, left ventricular; EES, everolimus-eluting stent; and SES, sirolimus-eluting stent.

DAPT. Prolonged DAPT for >6 months might be needed to prevent late stent thrombosis because of delayed vascular healing and inflammatory reaction after implantation of drug-eluting stents.[23,24] However, the majority of patients were taking both aspirin and clopidogrel at the time of stent thrombosis. Moreover, the timing of discontinuation of clopidogrel and stent thrombosis in our study called into question the temporal and causal relationship between discontinuation and thrombosis.

The interesting result in this study is that the treatment effect varied depending on the presence of diabetes mellitus. Among diabetic patients, target vessel failure occurred more frequently with 6-month DAPT than with 12-month DAPT. Although these results might have occurred by chance, several lines of evidences support our result. Diabetes mellitus is regarded as a proinflammatory and prothrombotic condition,[25,26] and patients with diabetes mellitus are more frequently resistant to aspirin than those without diabetes mellitus.[27] In an observational study of diabetes mellitus, longer use of clopidogrel was associated with a lower incidence of death or myocardial infarction after implantation of drug-eluting stents.[28] The minimum necessary duration of DAPT may be longer in diabetic than in nondiabetic patients. Although a significant interaction between other conditions and outcomes was not found, results from diabetic patients can suggest that longer DAPT may be needed in high-risk patients. These findings, however, should be interpreted with caution, although they were derived from the prespecified subgroup analysis.

There were several limitations to our study. First, the primary end point was target vessel failure rather than hard end points such as death or myocardial infarction. Considering the low rate of death or myocardial infarction in the present study, tens of thousands of patients need to be enrolled to compare death or myocardial infarction as the primary end point. However, we do not think that including revascularization could have biased our findings toward a neutral effect on outcomes because it is unlikely that a shorter duration of DAPT is superior to a longer one in terms of revascularization. Revascularization is also one of the important outcomes and might be included in the primary end point to test noninferiority of 6-month DAPT more rigorously. Second, the event rate was lower than expected, although estimates of the event rate were based on data from previous studies.[17,18] As a result, the noninferiority margin of 4.0 percentage points was quite wide, considering that the rate of target vessel failure was 4.3% with 12-month antiplatelet therapy. The low event rate might be explained by several possible factors. Our study was not an all-comer study, and patients with high risk such as those with myocardial infarction within 72 hours, left main lesions, or severe left ventricular dysfunction were excluded. Differences in interventional practice such as frequent use of intravascular ultrasound may play a role. Ethnic differences between our study and previous ones may be another potential contributor. Third, our study was an open-label trial and was not placebo controlled. This can affect study outcomes, including target vessel revascularization, which was 1 component of the primary end point, target vessel failure. Although all clinical end points were assessed by members of independent clinical event adjudication committee and statistical analyses were performed by independent statisticians, operators were not blinded to duration of clopidogrel. Fourth, apart from the American College of Cardiology/American Heart Association guideline for PCI,[8] clopidogrel is recommended for at least 12 months in patients receiving stent during PCI for acute coronary syndromes.[29] Patients who initially presented with acute coronary syndromes and were allocated to the 6-month DAPT group might be at higher risk of events than those whose initial presentation was stable angina. Finally, a considerable proportion of patients in the 6-month DAPT group received clopidogrel for >6 months. However, 6-month DAPT was also noninferior to 12-month DAPT in the per-protocol analysis. Larger ongoing randomized trial

such as the Safety and Efficacy of Six Months DAPT After Drug-Eluting Stenting (ISAR-SAFE) trial (NCT00661206) can provide more evidence regarding the safety of 6-month DAPT. Until more confirmative evidence of the safety of 6-month DAPT is obtained, 6-month DAPT cannot be recommended in the general population undergoing PCI. Meanwhile, our results may be helpful for physicians to decide the duration of DAPT case by case in real-world practice, eg, in patients with increased bleeding risk or undergoing elective surgery.

Conclusions

Our trial showed that the rate of target vessel failure was not significantly different between the 6- and 12-month DAPT groups after PCI with drug-eluting stents and that 6-month DAPT was noninferior to 12-month DAPT in the risk of target vessel failure. However, the noninferiority margin was wide, and the study was underpowered for hard end points such as death or myocardial infarction. The safety of a short duration of DAPT in terms of stent thrombosis or in diabetic patients should be studied in larger randomized trials.

Sources of Funding

This study was supported by a grant (A040152) from the Ministry of Health, Welfare, and Family Affairs, Korea; Abbott Vascular Korea; and Boston Scientific Korea. The sponsors had no access to the study data and had no role in the design, conduct, analysis, or reporting of the study.

Disclosures

None.

References

1. Moses JW, Leon MB, Popma JJ, Fitzgerald PJ, Holmes DR, O'Shaughnessy C, Caputo RP, Kereiakes DJ, Williams DO, Teirstein PS, Jaeger JL, Kuntz RE. Sirolimus-eluting stents versus standard stents in patients with stenosis in a native coronary artery. *N Engl J Med*. 2003; 349:1315–1323.
2. Stone GW, Ellis SG, Cox DA, Hermiller J, O'Shaughnessy C, Mann JT, Turco M, Caputo R, Bergin P, Greenberg J, Popma JJ, Russell ME. A polymer-based, paclitaxel-eluting stent in patients with coronary artery disease. *N Engl J Med*. 2004;350:221–231.
3. Fajadet J, Wijns W, Laarman GJ, Kuck KH, Ormiston J, Munzel T, Popma JJ, Fitzgerald PJ, Bonan R, Kuntz RE. Randomized, double-blind, multicenter study of the Endeavor zotarolimus-eluting phosphorylcholine-encapsulated stent for treatment of native coronary artery lesions: clinical and angiographic results of the ENDEAVOR II trial. *Circulation*. 2006;114:798–806.
4. Pfisterer M, Brunner-La Rocca HP, Buser PT, Rickenbacher P, Hunziker P, Mueller C, Jeger R, Bader F, Osswald S, Kaiser C. Late clinical events after clopidogrel discontinuation may limit the benefit of drug-eluting stents: an observational study of drug-eluting versus bare-metal stents. *J Am Coll Cardiol*. 2006;48:2584–2591.
5. Daemen J, Wenaweser P, Tsuchida K, Abrecht L, Vaina S, Morger C, Kukreja N, Juni P, Sianos G, Hellige G, van Domburg RT, Hess OM, Boersma E, Meier B, Windecker S, Serruys PW. Early and late coronary stent thrombosis of sirolimus-eluting and paclitaxel-eluting stents in routine clinical practice: data from a large two-institutional cohort study. *Lancet*. 2007;369:667–678.
6. Iakovou I, Schmidt T, Bonizzoni E, Ge L, Sangiorgi GM, Stankovic G, Airoldi F, Chieffo A, Montorfano M, Carlino M, Michev I, Corvaja N, Briguori C, Gerckens U, Grube E, Colombo A. Incidence, predictors, and outcome of thrombosis after successful implantation of drug-eluting stents. *JAMA*. 2005;293:2126–2130.
7. Eisenstein EL, Anstrom KJ, Kong DF, Shaw LK, Tuttle RH, Mark DB, Kramer JM, Harrington RA, Matchar DB, Kandzari DE, Peterson ED, Schulman KA, Califf RM. Clopidogrel use and long-term clinical outcomes after drug-eluting stent implantation. *JAMA*. 2007;297: 159–168.
8. King SB 3rd, Smith SC Jr, Hirshfeld JW Jr, Jacobs AK, Morrison DA, Williams DO, Feldman TE, Kern MJ, O'Neill WW, Schaff HV, Whitlow PL, Adams CD, Anderson JL, Buller CE, Creager MA, Ettinger SM, Halperin JL, Hunt SA, Krumholz HM, Kushner FG, Lytle BW, Nishimura R, Page RL, Riegel B, Tarkington LG, Yancy CW. 2007 Focused update of the ACC/AHA/SCAI 2005 guideline update for percutaneous coronary intervention: a report of the American College of Cardiology/American Heart Association Task Force on Practice Guidelines: 2007 Writing Group to Review New Evidence and Update the ACC/AHA/SCAI 2005 Guideline Update for Percutaneous Coronary Intervention, writing on behalf of the 2005 writing committee. *Circulation*. 2008;117:261–295.
9. Park SJ, Park DW, Kim YH, Kang SJ, Lee SW, Lee CW, Han KH, Park SW, Yun SC, Lee SG, Rha SW, Seong IW, Jeong MH, Hur SH, Lee NH, Yoon J, Yang JY, Lee BK, Choi YJ, Chung WS, Lim DS, Cheong SS, Kim KS, Chae JK, Nah DY, Jeon DS, Seung KB, Jang JS, Park HS, Lee K. Duration of dual antiplatelet therapy after implantation of drug-eluting stents. *N Engl J Med*. 2010;362:1374–1382.
10. Airoldi F, Colombo A, Morici N, Latib A, Cosgrave J, Buellesfeld L, Bonizzoni E, Carlino M, Gerckens U, Godino C, Melzi G, Michev I, Montorfano M, Sangiorgi GM, Qasim A, Chieffo A, Briguori C, Grube E. Incidence and predictors of drug-eluting stent thrombosis during and after discontinuation of thienopyridine treatment. *Circulation*. 2007;116: 745–754.
11. Kimura T, Morimoto T, Nakagawa Y, Tamura T, Kadota K, Yasumoto H, Nishikawa H, Hiasa Y, Muramatsu T, Meguro T, Inoue N, Honda H, Hayashi Y, Miyazaki S, Oshima S, Honda T, Shiode N, Namura M, Sone T, Nobuyoshi M, Kita T, Mitsudo K. Antiplatelet therapy and stent thrombosis after sirolimus-eluting stent implantation. *Circulation*. 2009; 119:987–995.
12. Hahn JY, Song YB, Choi JH, Choi SH, Lee SY, Park HS, Hur SH, Lee S, Han KR, Rha SW, Cho BR, Park JS, Yoon J, Lim do S, Lee SH, Gwon HC. Three-month dual antiplatelet therapy after implantation of zotarolimus-eluting stents: the DATE (Duration of Dual Antiplatelet Therapy After Implantation of Endeavor Stent) registry. *Circ J*. 2010;74: 2314–2321.
13. Park KW, Yoon JH, Kim JS, Hahn JY, Cho YS, Chae IH, Gwon HC, Ahn T, Oh BH, Park JE, Shim WH, Shin EK, Jang YS, Kim HS. Efficacy of Xience/promus versus Cypher in rEducing Late Loss after stENTing (EXCELLENT) trial: study design and rationale of a Korean multicenter prospective randomized trial. *Am Heart J*. 2009;157:811–817.e1.
14. Bovill EG, Terrin ML, Stump DC, Berke AD, Frederick M, Collen D, Feit F, Gore JM, Hillis LD, Lambrew CT, et al. Hemorrhagic events during therapy with recombinant tissue-type plasminogen activator, heparin, and aspirin for acute myocardial infarction: results of the Thrombolysis in Myocardial Infarction (TIMI), phase II trial. *Ann Intern Med*. 1991;115:256–265.
15. Cutlip DE, Windecker S, Mehran R, Boam A, Cohen DJ, van Es GA, Steg PG, Morel MA, Mauri L, Vranckx P, McFadden E, Lansky A, Hamon M, Krucoff MW, Serruys PW. Clinical end points in coronary stent trials: a case for standardized definitions. *Circulation*. 2007;115:2344–2351.
16. Fox KA, Poole-Wilson PA, Henderson RA, Clayton TC, Chamberlain DA, Shaw TR, Wheatley DJ, Pocock SJ. Interventional versus conservative treatment for patients with unstable angina or non-ST-elevation myocardial infarction: the British Heart Foundation RITA 3 randomised trial: Randomized Intervention Trial of unstable Angina. *Lancet*. 2002; 360:743–751.
17. Windecker S, Remondino A, Eberli FR, Juni P, Raber L, Wenaweser P, Togni M, Billinger M, Tuller D, Seiler C, Roffi M, Corti R, Sutsch G, Maier W, Luscher T, Hess OM, Egger M, Meier B. Sirolimus-eluting and paclitaxel-eluting stents for coronary revascularization. *N Engl J Med*. 2005;353:653–662.
18. Morice MC, Colombo A, Meier B, Serruys P, Tamburino C, Guagliumi G, Sousa E, Stoll HP. Sirolimus- vs paclitaxel-eluting stents in de novo coronary artery lesions: the REALITY trial: a randomized controlled trial. *JAMA*. 2006;295:895–904.
19. Anderson JR, Cain KC, Gelber RD. Analysis of survival by tumor response. *J Clin Oncol*. 1983;1:710–719.
20. Yusuf S, Zhao F, Mehta SR, Chrolavicius S, Tognoni G, Fox KK. Effects of clopidogrel in addition to aspirin in patients with acute coronary syndromes without ST-segment elevation. *N Engl J Med*. 2001;345: 494–502.

21. Hallas J, Dall M, Andries A, Andersen BS, Aalykke C, Hansen JM, Andersen M, Lassen AT. Use of single and combined antithrombotic therapy and risk of serious upper gastrointestinal bleeding: population based case-control study. *BMJ*. 2006;333:726.
22. Iwata Y, Kobayashi Y, Fukushima K, Kitahara H, Asano T, Ishio N, Nakayama T, Kuroda N, Komuro I. Incidence of premature discontinuation of antiplatelet therapy after sirolimus-eluting stent implantation. *Circ J*. 2008;72:340–341.
23. Finn AV, Joner M, Nakazawa G, Kolodgie F, Newell J, John MC, Gold HK, Virmani R. Pathological correlates of late drug-eluting stent thrombosis: strut coverage as a marker of endothelialization. *Circulation*. 2007;115:2435–2441.
24. Farb A, Burke AP, Kolodgie FD, Virmani R. Pathological mechanisms of fatal late coronary stent thrombosis in humans. *Circulation*. 2003;108:1701–1706.
25. Dichiara J, Bliden KP, Tantry US, Chaganti SK, Kreutz RP, Gesheff TB, Kreutz Y, Gurbel PA. Platelet function measured by VerifyNow identifies generalized high platelet reactivity in aspirin treated patients. *Platelets*. 2007;18:414–423.
26. DiChiara J, Bliden KP, Tantry US, Hamed MS, Antonino MJ, Suarez TA, Bailon O, Singla A, Gurbel PA. The effect of aspirin dosing on platelet function in diabetic and nondiabetic patients: an analysis from the Aspirin-Induced Platelet Effect (ASPECT) study. *Diabetes*. 2007;56:3014–3019.
27. Angiolillo DJ, Fernandez-Ortiz A, Bernardo E, Ramirez C, Sabate M, Jimenez-Quevedo P, Hernandez R, Moreno R, Escaned J, Alfonso F, Banuelos C, Costa MA, Bass TA, Macaya C. Influence of aspirin resistance on platelet function profiles in patients on long-term aspirin and clopidogrel after percutaneous coronary intervention. *Am J Cardiol*. 2006;97:38–43.
28. Brar SS, Kim J, Brar SK, Zadegan R, Ree M, Liu IL, Mansukhani P, Aharonian V, Hyett R, Shen AY. Long-term outcomes by clopidogrel duration and stent type in a diabetic population with de novo coronary artery lesions. *J Am Coll Cardiol*. 2008;51:2220–2227.
29. Kushner FG, Hand M, Smith SC Jr, King SB 3rd, Anderson JL, Antman EM, Bailey SR, Bates ER, Blankenship JC, Casey DE Jr, Green LA, Hochman JS, Jacobs AK, Krumholz HM, Morrison DA, Ornato JP, Pearle DL, Peterson ED, Sloan MA, Whitlow PL, Williams DO. 2009 Focused updates: ACC/AHA guidelines for the management of patients with ST-elevation myocardial infarction (updating the 2004 guideline and 2007 focused update) and ACC/AHA/SCAI guidelines on percutaneous coronary intervention (updating the 2005 guideline and 2007 focused update): a report of the American College of Cardiology Foundation/American Heart Association Task Force on Practice Guidelines. *Circulation*. 2009;120:2271–2306.

CLINICAL PERSPECTIVE

The optimal duration of dual antiplatelet therapy (DAPT) after implantation of drug-eluting coronary stents remains undetermined. Although premature discontinuation of thienopyridine therapy was reported to be the major determinant of stent thrombosis after implantation of drug-eluting stents, some studies suggest that there is no apparent clinical benefit from DAPT for >6 months. In the Efficacy of Xience/Promus Versus Cypher to Reduce Late Loss After Stenting (EXCELLENT) trial, we compared 6-month DAPT with 12-month DAPT in patients receiving drug-eluting stents. Our trial showed that the rate of target vessel failure was not significantly different between the 6- and 12-month DAPT groups after percutaneous coronary intervention with drug-eluting stents (4.8% versus 4.3%) and that 6-month DAPT was noninferior to 12-month DAPT in the risk of target vessel failure. However, stent thrombosis tended to occur more frequently in the 6-month DAPT group than in the 12-month group (0.9% versus 0.1%). In subgroup analysis, target vessel failure occurred more frequently in the 6-month DAPT group than in the 12-month group among diabetic patients (hazard ratio, 3.16; 95% confidence interval, 1.42–7.03). Although 6-month DAPT cannot be recommended in the general population on the basis of our trial, these data may be helpful for physicians to decide the duration of DAPT case by case in real-world practice, eg, in patients with increased bleeding risk or undergoing elective surgery.
