# Supplementary material for: Optimal duration of DAPT after second-generation drug-eluting stent in acute coronary syndrome
Source: PLoS One. 2018 Nov 26;13(11):e0207386. doi: 10.1371/journal.pone.0207386 (PMC6261023; doi:10.1371/journal.pone.0207386)
Supplement: S3 File — (PDF) [file pone.0207386.s006.pdf]

Original Investigation

Effect of Intravascular Ultrasound–Guided vs Angiography-Guided Everolimus-Eluting Stent Implantation
The IVUS-XPL Randomized Clinical Trial

Sung-Jin Hong, MD; Byeong-Keuk Kim, MD; Dong-Ho Shin, MD, MPH; Chung-Mo Nam, PhD; Jung-Sun Kim, MD; Young-Guk Ko, MD; Donghoon Choi, MD; Tae-Soo Kang, MD; Woong-Chol Kang, MD; Ae-Young Her, MD; Yong Hoon Kim, MD; Seung-Ho Hur, MD; Bum-Kee Hong, MD; Hyuckmoon Kwon, MD; Yangsoo Jang, MD; Myeong-Ki Hong, MD, PhD; for the IVUS-XPL Investigators

IMPORTANCE Use of intravascular ultrasound (IVUS) promotes better clinical outcomes for coronary intervention in complex coronary lesions. However, randomized data demonstrating the clinical usefulness of IVUS are limited for lesions treated with drug-eluting stents.

OBJECTIVE To determine whether the long-term clinical outcomes with IVUS-guided drug-eluting stent implantation are superior to those with angiography-guided implantation in patients with long coronary lesions.

DESIGN, SETTING, AND PARTICIPANTS The Impact of Intravascular Ultrasound Guidance on Outcomes of Xience Prime Stents in Long Lesions (IVUS-XPL) randomized, multicenter trial was conducted in 1400 patients with long coronary lesions (implanted stent ≥28 mm in length) between October 2010 and July 2014 at 20 centers in Korea.

INTERVENTIONS Patients were randomly assigned to receive IVUS-guided (n = 700) or angiography-guided (n = 700) everolimus-eluting stent implantation.

MAIN OUTCOMES AND MEASURES Primary outcome measure was the composite of major adverse cardiac events, including cardiac death, target lesion-related myocardial infarction, or ischemia-driven target lesion revascularization at 1 year, analyzed by intention-to-treat.

RESULTS One-year follow-up was complete in 1323 patients (94.5%). Major adverse cardiac events at 1 year occurred in 19 patients (2.9%) undergoing IVUS-guided and in 39 patients (5.8%) undergoing angiography-guided stent implantation (absolute difference, −2.97% [95% CI, −5.14% to −0.79%]) (hazard ratio [HR], 0.48 [95% CI, 0.28 to 0.83], *P* = .007). The difference was driven by a lower risk of ischemia-driven target lesion revascularization in patients undergoing IVUS-guided (17 [2.5%]) compared with angiography-guided (33 [5.0%]) stent implantation (HR, 0.51 [95% CI, 0.28 to 0.91], *P* = .02). Cardiac death and target lesion–related myocardial infarction were not significantly different between the 2 groups. For cardiac death, there were 3 patients (0.4%) in the IVUS-guided group and 5 patients (0.7%) in the angiography-guided group (HR, 0.60 [95% CI, 0.14 to 2.52], *P* = .48). Target lesion–related myocardial infarction occurred in 1 patient (0.1%) in the angiography-guided stent implantation group (*P* = .32).

CONCLUSIONS AND RELEVANCE Among patients requiring long coronary stent implantation, the use of IVUS-guided everolimus-eluting stent implantation, compared with angiography-guided stent implantation, resulted in a significantly lower rate of the composite of major adverse cardiac events at 1 year. These differences were primarily due to lower risk of target lesion revascularization.

TRIAL REGISTRATION clinicaltrials.gov Identifier: NCT01308281

JAMA. 2015;314(20):2155-2163. doi:10.1001/jama.2015.15454
Published online November 10, 2015. Corrected on February 2, 2016.

Supplemental content at jama.com

Author Affiliations: Author affiliations are listed at the end of this article.

Group Information: The IVUS-XPL Investigators are listed at the end of this article.

Corresponding Author: Myeong-Ki Hong, MD, PhD, Division of Cardiology, Severance Cardiovascular Hospital, Yonsei University College of Medicine, 134 Sinchon-dong, Seodaemun-gu, Seoul 120-752, Korea (mkhong61@yuhs.ac).

Copyright 2015 American Medical Association. All rights reserved.

Downloaded From: by a MEDICAL LIBRARY YONSEI UNIVERSITY User on 06/15/2018

The introduction of the drug-eluting stent (DES) has contributed to a significant reduction in in-stent restenosis and repeat revascularization.[1,2] However, despite the use of the DES, percutaneous coronary intervention (PCI) of diffuse long coronary lesions still remains challenging because the prevalence of in-stent restenosis and stent thrombosis remains high compared with short-length coronary lesions.[3,4]

During the PCI procedure, intravascular ultrasound (IVUS) may be a useful tool for providing information on preintervention lesion characteristics, including vulnerable plaques, lesion severity, length, and morphology; on postintervention optimal stent implantation for stent expansion, extension, and apposition; and on possible complications after stent implantation.[5-7]

Four meta-analyses showed that IVUS-guided DES implantation was associated with a significant reduction in major adverse cardiac events, stent thrombosis, and target lesion revascularization (TLR).[8-11] Even though recent guidelines recommend the use of IVUS to optimize stent implantation for select patients, the effect of IVUS-guided DES implantation on clinical outcomes remains uncertain because of the limited number of properly powered randomized trials.[12-15] In addition, first-generation DESs were commonly used in most studies for investigating the IVUS-guided DES implantation.[5-7,12] However, second-generation DESs are now exclusively used for PCI in current clinical practice, and determining the clinical usefulness of IVUS guidance in the implantation of these DESs is needed.

DES *drug-eluting stent*

IVUS *intravascular ultrasound*

PCI *percutaneous coronary intervention*

TLR *target lesion revascularization*

The Impact of Intravascular Ultrasound Guidance on Outcomes of Xience Prime Stents in Long Lesions (IVUS-XPL) randomized, multicenter trial was conducted to evaluate the clinical benefits of IVUS in patients who underwent everolimus-eluting stent implantation for long coronary lesions.

Methods

Study Design

The IVUS-XPL trial was an investigator-initiated, randomized, multicenter study conducted at 20 centers in Korea with patients who received an everolimus-eluting stent (Xience prime, Abbott Vascular) implantation for long coronary lesions. The detailed rationale and design of this study have been previously described.[16] We hypothesized that the long-term clinical outcomes of patients undergoing IVUS-guided everolimus-eluting stent implantation would be superior to patients undergoing angiography-guided everolimus-eluting stent implantation for long coronary lesions. Study coordination, data management, and site management services were performed at the Cardiovascular Research Center, Seoul, Korea. The designated trial monitors reviewed the investigational data at appropriate intervals for accuracy and completeness and ensured compliance with the protocol.[16] A data and safety monitoring board, composed of independent physicians with access to the unblinded data, monitored the safety of the study.

Study Population

Patients with typical chest pain or evidence of myocardial ischemia were eligible for enrollment if implantation of an everolimus-eluting stent for long coronary lesions (implanted stent ≥28 mm in length) was indicated based on angiographic estimation.[14,16] The detailed information for inclusion and exclusion criteria has been previously described.[16] The study protocol was approved by the institutional review boards or ethics committees at each participating center, and all participants gave written informed consent. The trial protocol appears in Supplement 1.

Randomization and Study Procedures

Using an interactive web-based response system, study participants were randomly assigned in a 1:1 ratio (**Figure 1**) to receive either IVUS-guided or angiographic-guided stent implantation immediately after the pre-PCI angiogram with the use of a block size of 4 for the 2 study groups. Concealed randomization was stratified based on enrolling sites, multivessel PCI, and diabetes mellitus.

The detailed information regarding the study procedures according to either IVUS-guided or angiography-guided stent implantation is described in Supplement 1. Neither the patients nor the treating physicians were blinded to the treatment procedures performed. Everolimus-eluting stent implantation was performed according to standard techniques. In the angiography-guided stent implantation group, stent size and length were chosen by visual estimation, and adjunct high-pressure dilation was performed if an optimal result, defined as angiographic residual diameter stenosis of less than 30% by visual estimation and the absence of angiographically detected dissection, was not achieved.[16]

In the IVUS-guided stent implantation group, stent size and length were selected by online IVUS measurements, and adjunct high-pressure dilation was performed according to the discretion of the physicians, based on the IVUS findings. Use of IVUS was allowed at any step of PCI (before, during, or after PCI). IVUS examination before and during PCI was not mandatory; however, IVUS examination was mandatory after PCI.[16] In the present study, IVUS criteria for stent optimization after PCI was defined as a minimal lumen cross-sectional area greater than the lumen cross-sectional area at the distal reference segments.

After stent implantation, aspirin (at a dose of 100 mg/d) was prescribed indefinitely. Clopidogrel (at a dose of 75 mg/d) was administered for more than 6 months following stent implantation for all patients according to the study protocol (Supplement 1).

Study End Points and Follow-up

The primary end point was a composite of major adverse cardiac events, including cardiac death, target lesion–related myocardial infarction, or ischemia-driven TLR at 1 year. Clinical events were defined according to the Academic Research Con-

Copyright 2015 American Medical Association. All rights reserved.

Downloaded From: by a MEDICAL LIBRARY YONSEI UNIVERSITY User on 06/15/2018

Figure 1. Flow of Participants in the Impact of Intravascular Ultrasound Guidance on Outcomes of Xience Prime Stents in Long Lesions Trial

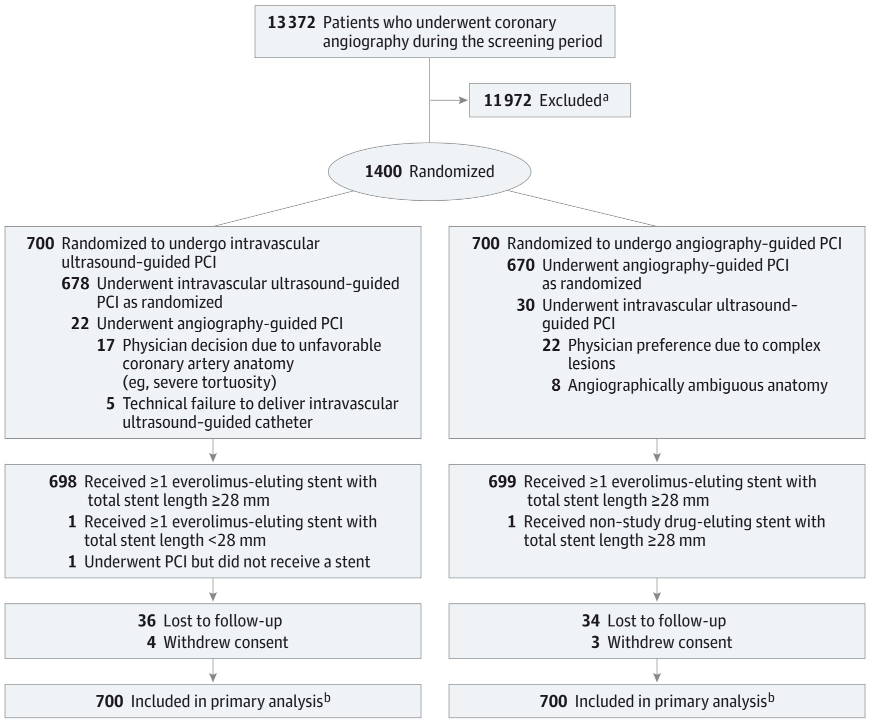

PCI indicates percutaneous coronary intervention.

[a] Data were not collected regarding specific reasons for ineligibility.

[b] All patients were included in the primary time to event analysis for the duration of their follow-up, including patients who withdrew consent or were lost to follow-up.

sortium and were previously described.[16,17] All deaths were considered cardiac deaths unless a definite noncardiac cause could be established. Target lesion–related myocardial infarction during the 1-year follow-up after hospital discharge was defined as the presence of clinical symptoms, electrocardiographic changes, or abnormal imaging findings of myocardial infarction, combined with an increase in the creatine kinase MB fraction above the upper normal limits or an increase in troponin T or troponin I to a level greater than the 99th percentile of the upper normal limit.

The territory of the myocardial infarction was supplied by the coronary artery containing the stented lesions (implanted stent ≥28 mm in length).[16-18] Clinically relevant periprocedural myocardial infarction after PCI was defined as a peak creatine kinase MB fraction of 10 or more times the upper limit measured within 48 hours of the procedure, or of 5 or more times the upper normal limit, with new pathological Q waves in 2 or more contiguous leads, or new persistent left bundle-branch block according to the expert consensus document from the Society for Cardiovascular Angiography and Interventions.[19]

Definite, probable, and possible stent thrombosis was defined according to the recommendations of the Academic Research Consortium.[16,17,20] Ischemia-driven TLR was defined as a repeat PCI or bypass surgery of the target lesions with either of the following: (1) symptoms of ischemia or a positive stress test and angiographic diameter stenosis of 50% or greater by quantitative coronary angiographic analysis, or (2) angiographic diameter stenosis of 70% or greater by quantitative coronary angiographic analysis without symptoms of ischemia or a positive stress test.[16]

Postprocedural clinical assessment, including the evaluation of cardiac symptoms and compliance with medications, were performed in the hospital and after 1, 3, 6, and 12 months at the physician office visit. During follow-up, data were collected and entered into a computer database by a specialist from a clinical data management center (Cardiovascular Research Center, Seoul, Korea).[16] A blinded independent clinical events committee adjudicated all nonprocedural components of the primary end point. The detailed descriptions of the angiographic and IVUS analyses were presented in the previous study.[16,21]

Statistical Analysis

Calculation of the sample size was based on a 2-sample and 2-sided test. We assumed the overall incidence of major adverse cardiac events, including cardiac death, myocardial infarction, or ischemia-driven TLR, to be 7% at the 1-year follow-up in the angiography-guided stent implantation group.[14,16,22-26] This study was designed as a superiority trial, with an expected risk reduction of 50% in the IVUS-guided stent implantation group for the primary end point.[27] Therefore, 700 patients were needed for each group, assuming a 2-sided α level of .05, statistical power of 80%, and an estimated dropout rate of 5% to 10% (more details appear in Supplement 1).

The primary analysis was performed with an intention-to-treat analysis to compare whether IVUS-guided stent im-

Copyright 2015 American Medical Association. All rights reserved.

Downloaded From: by a MEDICAL LIBRARY YONSEI UNIVERSITY User on 06/15/2018

Table 1. Baseline Clinical Characteristics

	IVUS-Guided PCI[a]	Angiography-Guided PCI[a]
No. of patients	700	700
Age, mean (SD), y	64 (9)	64 (9)
Male sex	483 (69)	481 (69)
Body mass index, mean (SD)[b]	24.6 (3.0)	24.8 (3.1)
Hypertension	454 (65)	444 (63)
Diabetes mellitus	250 (36)	256 (37)
Insulin-dependent diabetes	22 (3)	23 (3)
Dyslipidemia	471 (67)	458 (65)
Current smoker	155 (22)	181 (26)
Prior myocardial infarction	34 (5)	29 (4)
Prior PCI	76 (11)	69 (10)
Prior coronary artery bypass graft	20 (3)	16 (2)
LVEF, mean (SD), %	62.9 (9.8)	62.4 (10.2)
Clinical presentation		
Stable angina	358 (51)	356 (51)
Unstable angina	242 (35)	226 (32)
Acute myocardial infarction	100 (14)	118 (17)
No. of diseased vessels		
1	230 (33)	210 (30)
2	256 (37)	260 (37)
3	214 (31)	230 (33)
No. of treated lesions per patient, mean (SD)	1.34 (0.56)	1.36 (0.57)
Duration of dual antiplatelet treatment, median (IQR), d	365 (180-365)	365 (180-365)
Medications at discharge		
Statins	669 (96)	670 (96)
β-Blockers	501 (72)	479 (68)
ACE inhibitors	181 (26)	198 (28)
Angiotensin II receptor blockers	242 (35)	240 (34)
Calcium channel blockers	235 (34)	236 (34)

Abbreviations: ACE, angiotensin-converting enzyme; IQR, interquartile range; IVUS, intravascular ultrasound; LVEF, left ventricular ejection fraction; PCI, percutaneous coronary intervention.

[a] Data are expressed as No. (%) unless otherwise indicated.

[b] Calculated as weight in kilograms divided by height in meters squared.

plantation would be superior to angiography-guided stent implantation with respect to the first occurrence of the primary end point. Cumulative incidences of major adverse cardiac events at 1 year, which was the primary end point, were calculated using the Kaplan-Meier estimates and compared using the log-rank test. Information on patients who were lost to follow-up (n = 70, 5.0%) or withdrew consent (n = 7, 0.5%) were used as censored data in the survival analysis. Although patients could experience more than 1 component of the primary end point, each patient was assessed until the occurrence of their first event and only once during the analysis. Subgroup analysis was performed according to the prespecified subgroups. Heterogeneity of the effects in subgroups was assessed using interaction terms in the Cox proportional hazard model.

Categorical variables were reported as numbers and percentages and were compared using the χ^2 test or Fisher exact test. Continuous variables were reported as the mean (standard deviation) or median (interquartile range) as appropriate, and these variables were compared using the *t* test or the Mann-Whitney test. All analyses were conducted using SAS version 9.2 (SAS Institute Inc). All tests were 2-sided and a *P* value of less than .05 was considered statistically significant.

Results

During the enrollment period between October 2010 and July 2014, a total of 1400 patients were randomly assigned to receive either IVUS-guided stent implantation (700 patients) or angiography-guided stent implantation (700 patients). Patient assignment and follow-up are detailed in Figure 1. A total of 77 patients withdrew consent or were lost to follow-up.

Baseline clinical, angiographic, and procedural characteristics were well balanced in both groups (**Table 1**, **Table 2**, and eTable 1 in Supplement 2). The mean (SD) age of all patients was 64 (9) years and 69% were men. The mean (SD) stented length of target lesions was 39.3 (12.7) mm. During the procedure, adjunct poststent balloon dilation was more frequently performed in the IVUS-guided stent group (76%; 534 patients) than in the angiography-guided stent group (57%; 402 patients) (absolute difference, 19% [95% CI, 14%-24%]; $P < .001$; Table 2). The mean final balloon size was larger in the IVUS-guided stent group than in the angiography-guided stent group. Consequently, on postprocedural quantitative angiography analysis, minimum lumen diameter was greater and diameter stenosis was smaller in the IVUS-guided stent group than in the angiography-guided stent group.

In the postintervention IVUS analysis, the mean (SD) IVUS-measured postintervention minimal lumen area was 5.90 (1.82) mm^2, and the number of patients meeting the IVUS criteria for stent optimization was 363 (54%) (**Table 3**). The patients who met the IVUS criteria had a significantly greater mean postintervention minimal lumen area at the stented segment and a smaller distal reference segment lumen area compared with those who did not meet the IVUS criteria (Table 3). Periprocedural myocardial infarction was not significantly different between the groups (11 patients [1.6%] in the IVUS-guided stent group vs 9 patients [1.3%] in the angiography-guided stent group; absolute difference, 0.3% [95% CI, −1.0% to 1.5%]; $P = .65$).

Clinical outcomes appear in **Table 4**. One-year follow-up was complete in 1323 patients (94.5%). At 1 year, the primary end point of major adverse cardiac events occurred in 19 patients (2.9%) undergoing IVUS-guided stent implantation and in 39 patients (5.8%) undergoing angiography-guided stent implantation (hazard ratio [HR], 0.48 [95% CI, 0.28 to 0.83]; $P = .007$) (absolute difference, −2.97% [95% CI, −5.14% to −0.79%]) (Table 4 and **Figure 2**A). For cardiac death alone, there were 3 patients (0.4%) in the IVUS-guided stent group and 5 patients (0.7%) in the angiography-guided stent group (HR, 0.60 [95% CI, 0.14 to 2.52]; $P = .48$). Target lesion-related myocardial infarction occurred in 1 patient (0.1%) in the

Copyright 2015 American Medical Association. All rights reserved.

Downloaded From: by a MEDICAL LIBRARY YONSEI UNIVERSITY User on 06/15/2018

Table 2. Angiographic and Procedural Characteristics for Target Lesions

	IVUS-Guided PCI	Angiography-Guided PCI	*P* Value
No. of patients with lesions	700	700	
Coronary arteries, No. (%)			
Left anterior descending artery	455 (65)	419 (60)	.14
Left circumflex artery	96 (14)	108 (15)	
Right coronary artery	149 (21)	173 (25)	
Baseline quantitative coronary angiographic data, mean (SD)			
Reference vessel diameter, mm	2.89 (0.45)	2.85 (0.45)	.13
Minimum lumen diameter, mm	0.83 (0.42)	0.82 (0.43)	.56
Diameter stenosis, %	71.1 (14.3)	71.4 (14.4)	.70
Lesion length, mm	34.7 (10.8)	35.2 (10.5)	.41
Adjunct postdilatation, No. (%)	534 (76)	402 (57)	<.001
Final balloon size, mean (SD), mm	3.14 (0.43)	3.04 (0.42)	<.001
Overlapping stent, No. (%)	145 (21)	138 (20)	.64
No. of stents per lesion, mean (SD)	1.3 (0.5)	1.3 (0.5)	.48
Stent edge dissections, No. (%)	15 (2)	13 (2)	.70
Coronary perforation, No. (%)	0	0	
Maximal inflation pressure, mean (SD), atm	16.5 (4.1)	15.9 (4.1)	.05
Postintervention quantitative coronary angiographic data, mean (SD)			
Total stented length, mm	39.3 (13.1)	39.2 (12.3)	.90
Reference vessel diameter, mm	3.03 (0.44)	2.97 (0.43)	.01
Minimum lumen diameter, mm	2.64 (0.42)	2.56 (0.39)	<.001
Diameter stenosis, %	12.79 (8.66)	13.74 (8.05)	.04

Abbreviations: IVUS, intravascular ultrasound; PCI, percutaneous coronary intervention.

Table 3. Postintervention Intravascular Ultrasound (IVUS) Analysis of Target Long Lesions

	Patients in the IVUS-Guided PCI Group Who Underwent IVUS-Guided Stent Implantation		
	Met Criteria[a]	Did Not Meet Criteria	*P* Value
No. (%) of patients[b]	363 (54)	315 (46)	
Adjunct postdilatation, No. (%)	282 (78)	237 (75)	.34
Final balloon size, mean (SD), mm	3.15 (0.45)	3.13 (0.42)	.52
Maximal inflation pressure, mean (SD), atm	16.5 (3.9)	16.4 (4.4)	.87
Proximal reference, mean (SD), mm^2			
External elastic membrane area	17.52 (5.34)	17.27 (5.04)	.56
Lumen area	9.02 (3.51)	8.86 (3.27)	.57
Minimal lumen area, mean (SD), mm^2	6.09 (1.91)	5.71 (1.71)	.008
Distal reference, mean (SD), mm^2			
External elastic membrane area	9.44 (3.98)	10.94 (3.83)	<.001
Lumen area	5.55 (1.82)	6.83 (1.68)	<.001

Abbreviation: PCI, percutaneous coronary intervention.

[a] Defined as having a minimal lumen cross-sectional area greater than the lumen cross-sectional area at distal reference segments.

[b] Twenty-two patients did not receive IVUS-guided stent implantation even though they were randomized to that treatment group.

angiography-guided stent group (*P* = .32). Ischemia-driven TLR was required in 17 patients (2.5%; 15 patients with ischemic symptoms or a positive test and angiographic diameter stenosis ≥50% by quantitative coronary angiographic analysis and 2 patients with angiographic ≥70% by quantitative coronary angiographic analysis without ischemic symptoms or a positive stress test) in the IVUS-guided stent group and in 33 patients (5.0%; 30 and 3 patients, respectively) in the angiography-guided stent group (HR, 0.51 [95% CI, 0.28 to 0.91]; *P* = .02). The risk reduction of major adverse cardiac events was achieved in 48% of the IVUS-guided stent group.

Prespecified subgroup analyses showed no statistically significant interactions among the subgroups (eFigure in Supplement 2). In the post hoc analysis among the patients within the IVUS-guided stent group, the patients who did not meet the IVUS criteria had a significantly higher incidence of the primary end point compared with those meeting the IVUS criteria for stent optimization (4.6% vs 1.5%, respectively; HR, 0.31 [95% CI, 0.11-0.86], *P* = .02; Figure 2B). In addition, the per-protocol based comparison for the primary end point of major adverse cardiac events was consistent with the intention-to-treat comparison. At 1 year, the major adverse cardiac events occurred in 2.8% in the patients who underwent IVUS-guided stent implantation (n = 708) and in 5.9% in those who underwent angiography-guided stent implantation (n = 692) (HR, 0.47 [95% CI, 0.27-0.82]; *P* = .007).

Copyright 2015 American Medical Association. All rights reserved.

Downloaded From: by a MEDICAL LIBRARY YONSEI UNIVERSITY User on 06/15/2018

Table 4. Clinical Outcomes at 1 Year

	IVUS-Guided PCI (n = 700)[a]	Angiography-Guided PCI (n = 700)[a]	Risk Difference (95% CI)	Hazard Ratio (95% CI)[b]	*P* Value[c]
**Primary End Point**					
Major adverse cardiac event[d]	19 (2.9)	39 (5.8)	−2.97 (−5.14 to −0.79)	0.48 (0.28 to 0.83)	.007
**Secondary End Point**					
Cardiac death	3 (0.4)	5 (0.7)	−0.30 (−1.11 to 0.52)	0.60 (0.14 to 2.52)	.48
Target lesion–related myocardial infarction	0	1 (0.1)	−0.15 (−0.45 to 0.14)		.32
Ischemia-driven target lesion revascularization	17 (2.5)	33 (5.0)	−2.39 (−4.43 to −0.36)	0.51 (0.28 to 0.91)	.02
Definite or probable stent thrombosis	2 (0.3)	2 (0.3)	0 (−0.57 to 0.56)	1.00 (0.14 to 7.10)	>.99
Acute	1 (0.1)	1 (0.1)			
Subacute	1 (0.1)	0			
Late	0	1 (0.1)			

Abbreviations: IVUS, intravascular ultrasound; PCI, percutaneous coronary intervention.

[a] Data are expressed as No. of patients (cumulative 1-year Kaplan-Meier event rate percentage).

[b] Derived from Cox proportional hazard regression models.

[c] Calculated using the log-rank test.

[d] Included cardiac death, target lesion–related myocardial infarction, or ischemia-driven target lesion revascularization at 1 year.

Figure 2. Kaplan-Meier Estimates of Occurrence of Primary End Point for All Patients and for Patients Who Underwent IVUS-Guided Stent Implantation

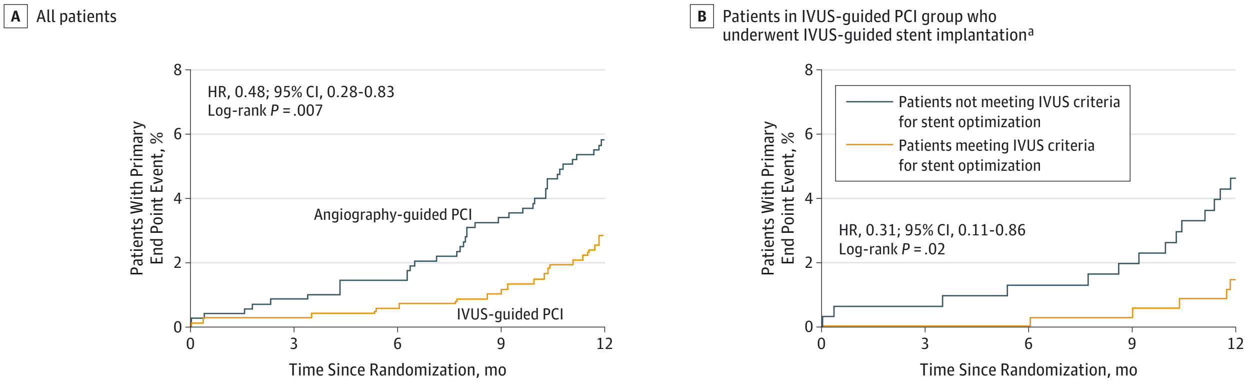

No. at risk	0	3	6	9	12
PCI					
Angiography-guided	700	673	660	643	624
IVUS-guided	700	671	665	654	641

No. at risk	0	3	6	9	12
IVUS criteria					
Not meeting	315	299	297	394	285
Meeting	363	362	345	338	334

Cumulative incidence curves for the primary end point of cardiac death, target lesion–related myocardial infarction, and target lesion revascularization. HR indicates hazard ratio; IVUS, intravascular ultrasound; PCI, percutaneous coronary intervention.

[a] There were 30 patients in the angiography-guided PCI group who underwent IVUS-guided PCI but they are not included in this analysis.

Discussion

In this randomized, multicenter trial of patients with long coronary lesions, the use of IVUS-guided stent implantation was associated with a significant 2.9% absolute reduction and 48% relative reduction in the risk of major adverse cardiac events at 1 year compared with angiography-guided stent implantation. These differences were mainly driven by the reduction of TLR in the IVUS-guided group. Accordingly, our findings suggest better clinical outcomes for major adverse cardiac events with IVUS-guided stent implantation compared with angiography-guided stent implantation, particularly for diffuse long lesions.

Whether IVUS-guided stent implantation will lead to improved clinical outcomes remains uncertain. Even though 4 meta-analyses (data mainly from observational studies) indicated that IVUS-guided stent implantation was associated with better clinical outcomes,[8-11] conclusive evidence regarding the effect of IVUS guidance on the clinical outcomes of patients implanted exclusively with second-generation DESs is limited. For instance, in 1 meta-analysis that included the largest number of DES-treated patients (26 503 total patients from 3 randomized trials and 14 observational studies), the proportion of patients who received second-generations DESs was less than 45% of the overall population. These findings showed that most of IVUS studies were composed of patients treated with first-generation

Copyright 2015 American Medical Association. All rights reserved.

Downloaded From: by a MEDICAL LIBRARY YONSEI UNIVERSITY User on 06/15/2018

DES only or in those treated with both first- and second-generation DESs; a small number randomized studies with a small number of patients were included in the meta-analyses.[9-11] Subsequently, these meta-analyses show significant heterogeneities.[11]

To our knowledge, the current study is the first demonstration of the clinical benefit of IVUS guidance in second-generation DES implantation in an adequately powered randomized clinical trial. There have been 3 previously reported randomized trials comparing the clinical usefulness of IVUS-guided vs angiography-guided stent implantation in the DES era.[12-14] In 2 trials, fewer than 150 patients in each group were included in the study, and the average implanted stent length was less than 24 mm in either the IVUS-guided or angiography-guided groups.[12,13]

Furthermore, the study by Jakabcin et al[12] used first-generation DESs, whereas the DES type was not clearly described in the second study Chieffo et al.[13] In a third randomized study that used second-generation DESs, the average total stent length was 32.4 mm in the IVUS-guided group (n = 269 patients) and 32.3 mm in the angiography-guided group (n = 274 patients).[14] Although the strategy of routine IVUS for DES implantation for diffuse long lesions did not improve the 1-year major adverse cardiac events rates in the intention-to-treat analysis, IVUS use per physician decision was associated with improved results in the per-protocol analysis (4.0% vs 8.1%, respectively; $P < .05$ in the per-protocol analysis).[14,16]

In the present study, a total of 1400 patients were randomized, the average stent length was 39.3 mm, and all patients underwent implantation with a second-generation DES. This large number of patients with lesions longer than in previous randomized trials leads to a sufficiently powered study able to prove the clinical usefulness of guidance with IVUS for second-generation DES implantation. Our results indicate that guidance with IVUS positively affects the clinical cardiovascular outcomes at 1 year compared with guidance with angiography. The clinical benefit of IVUS-guided DES implantation may be attributed to the larger minimal lumen diameter followed by the more frequent adjunct postdilation with a large-sized balloon in the IVUS-guided group.

Consistent with our findings, a larger postprocedural minimal lumen diameter is believed to be a major contributing factor for the prevention of restenosis after DES implantation.[3,28] Supporting this premise, a recent randomized trial of 230 patients with chronic total occlusion lesions revealed that IVUS-guided DES implantation was significantly associated with greater minimal lumen diameter and less late lumen loss (0.28 mm vs 0.46 mm, $P = .03$) with a lower rate of in-stent restenosis (3.9% vs 13.7%, $P = .02$) at 1-year angiographic follow-up.[29] In this study, the proportion of second-generation DESs was 27.8% in IVUS-guided group (n = 115) and 20.0% in angiography-guided group (n = 115).[29]

However, due to the small study population, this trial[29] was not sufficiently powered to make a statistically significant conclusion with respect to the hard end point, similarly to the previously published randomized trials.[12-14] Collectively, the data indicate that the importance of a larger minimal lumen diameter, particularly after long DES implantations, could be essential. Also, in our post hoc analysis for predictors of ischemia-driven TLR, postintervention minimal lumen diameter was an independent predictor of TLR (eTable 2 in Supplement 2).

One prospective, multicenter registry study and several meta-analysis studies with patients treated with both first- and second-generation DESs reported a significant reduction of major adverse cardiac events, including death, nonfatal myocardial infarction, stent thrombosis, or TLR in the patients undergoing IVUS-guided stent implantation compared with those undergoing angiography-guided implantation.[8-11,30] In complex lesion subsets, previous observational IVUS studies also showed that while IVUS-guided implantation was significantly associated with reduced cardiac death or nonfatal myocardial infarction in patients with non–left main bifurcation or left main lesions treated with first-generation DESs, no effect on TLR was observed.[31,32] This finding differs from our current findings, which show no differences in cardiac death, target lesion–related myocardial infarction, or stent thrombosis, and a statistically significant reduction in TLR in the IVUS-guided group compared with the angiography-guided group.

These divergent findings may result from the exclusive use of first-generation DESs or the combined use of first- and second-generation DESs in the previous studies,[8-11,30-32] whereas our study exclusively implanted second-generation DESs. One optical coherence tomographic study reported that the everolimus-eluting stent showed more favorable strut coverage than the first-generation sirolimus-eluting stent.[33] Previous meta-analysis also reported that the lowest rate of stent thrombosis was observed in the everolimus-eluting stent implantation among different types of DES.[34] Improved stent performance with the everolimus-eluting stent may be associated with no statistically significant difference of cardiac death, target lesion–related myocardial infarction, or stent thrombosis between IVUS-guided and angiography-guided DES implantation in the present study.

There are some limitations to the present study. First, there are currently no established defined criteria for DES optimization to prove favorable clinical outcomes. As such, IVUS criteria after PCI for stent optimization for long coronary lesions are arbitrarily defined in this study.[16] Second, it is possible that the angiography-guided procedure used in this study is not completely exclusive of the IVUS-guided technique. The physicians used in this study were proficient in both approaches, and their expert knowledge of IVUS may have unintentionally biased their approach when using angiography guidance.[16] Third, our study does not address cardiac events beyond the 1 year of follow-up. Fourth, due to the different study procedures for (angiography-guided or IVUS-guided) DES implantations, blinding the patients and treating physicians to the treatment was not feasible. However, we minimized the risk for any potential bias by using an end point analysis with precisely defined criteria, using core laboratories, blinding the adjudication by event adjudication committee members, and analyzing the data using intention-to-treat measures. Fifth, the observed overall event rate for the primary end point was lower than anticipated.

Copyright 2015 American Medical Association. All rights reserved.

Downloaded From: by a MEDICAL LIBRARY YONSEI UNIVERSITY User on 06/15/2018

Conclusions

Among patients requiring long coronary stent implantation, the use of IVUS-guided everolimus-eluting stent implantation, compared with angiography-guided stent implantation, resulted in a significantly lower rate of the composite of major adverse cardiac events at 1 year. These differences were primarily due to lower risk of target lesion revascularization.

ARTICLE INFORMATION

Published Online: November 10, 2015. doi:10.1001/jama.2015.15454.

Correction: This article was corrected online February 2, 2016, to fix the incorrect spelling of an author's name.

Author Affiliations: Department of Internal Medicine, Sanggye Paik Hospital, Inje University, Seoul, Korea (S.-J. Hong); Division of Cardiology, Severance Cardiovascular Hospital, Yonsei University College of Medicine, Seoul, Korea (S.-J. Hong, B.-K. Kim, Shin, J.-S. Kim, Ko, Choi, Jang, M.-K. Hong); Department of Preventive Medicine and Biostatistics, Yonsei University College of Medicine, Seoul, Korea (Nam); Dankook University College of Medicine, Cheonan, Korea (T.-S. Kang); Gil Hospital, Gachon University College of Medicine, Incheon, Korea (W.-C. Kang); School of Medicine, Kangwon National University, Chuncheon, Korea (Her, Y. H. Kim); Keimyung University College of Medicine, Daegu, Korea (Hur); Kangnam Severance Hospital, Seoul, Korea (B.-K. Hong, Kwon); Severance Biomedical Science Institute, Yonsei University College of Medicine, Seoul, Korea (Jang, M.-K. Hong); Cardiovascular Research Institute, Yonsei University College of Medicine, Seoul, Korea (Jang, M.-K. Hong).

Author Contributions: Drs B.-K. Kim and M.-K. Hong had full access to all of the data in the study and take responsibility for the integrity of the data and the accuracy of the data analysis. Drs S.-J. Hong and B.-K. Kim contributed equally to this article.
Study concept and design: S.-J. Hong, B.-K. Kim, Shin, Nam, J.-S. Kim, Ko, Choi, T.-S. Kang, W.-C. Kang, Y. H. Kim, Hur, Hong, Kwon, Jang, M.-K. Hong.
Acquisition, analysis, or interpretation of data: S.-J. Hong, B.-K. Kim, Shin, Nam, Ko, Choi, T.-S. Kang, W.-C. Kang, Her, Y. H. Kim, Hur, Hong, Kwon, Jang, M.-K. Hong.
Drafting of the manuscript: S.-J. Hong, B.-K. Kim, Kwon, M.-K. Hong.
Critical revision of the manuscript for important intellectual content: All authors.
Statistical analysis: B.-K. Kim, Shin, Nam.
Obtained funding: M.-K. Hong.
Administrative, technical, or material support: B.-K. Kim, Choi, Her, M.-K. Hong.
Study supervision: B.-K. Kim, Choi, Jang, M.-K. Hong.

Conflict of Interest Disclosures: The authors have completed and submitted the ICMJE Form for Disclosure of Potential Conflicts of Interest and none were reported.

Funding/Support: This study was supported by the Cardiovascular Research Center (Seoul, Korea) and funded by Abbott Vascular Inc.

Role of the Funder/Sponsor: Neither funder/sponsor had any role in the design and conduct of the study; collection, management, analysis, and interpretation of the data; preparation, review, or approval of the manuscript; or decision to submit the manuscript for publication.

Group Information: The IVUS-XPL Trial Investigators were Joo-Young Yang, Yangsoo Jang, Hyuckmoon Kwon, Jung-Han Yoon, Dong Woon Cheon, Myeong-Ki Hong (principal investigator), Seung Whan Lee, Byung-Ok Kim, and Bum-Kee Hong (steering committee); Yangsoo Jang, Hyuckmoon Kwon, Jung-Han Yoon, Dong Woon Cheon, Myeong-Ki Hong, and Byung-Ok Kim (executive committee); Chul-Min Ahn, Hyuck-Jai Chang, Seong-Hoon Choi, and Deok-Kyu Cho (data and safety monitoring board); Eui-Young Choi, Ji-Young Shim, Se-Jung Yoon, and Jang-Young Kim (clinical event adjudication committee).

Clinical Investigators: Sung-Jin Hong, Byeong-Keuk Kim, Dong-Ho Shin, Chung-Mo Nam, Jung-Sun Kim, Young-Guk Ko, Donghoon Choi, Yangsoo Jang, and Myeong Ki Hong (Yonsei University College of Medicine, Seoul, Korea); Bum-Kee Hong and Hyuckmoon Kwon (Gangnam Severance Hospital, Seoul, Korea); Sang-Gon Lee (Ulsan University Hospital, Ulsan, Korea); Jung Han Yoon (Wonju Christian Hospital, Wonju, Korea); Woong-Chol Kang (Gachon Gil University Hospital, Incheon, Korea); Tae-Soo Kang (Dankook University Hospital, Cheonan, Korea); Dong-Woon Jeon (NHIC Ilsan Hospital, Ilsan, Korea); Yun-Hyeong Cho (Myongji Hospital, Goyang, Korea); Jae-Woong Choi (Seoul Eulji Hospital, Seoul, Korea); Sang-Jae Rhee (Wonkwang University Hospital, Iksan, Korea); Rak-Kyeong Choi (Sejong General Hospital, Bucheon, Korea); Sung-Yoon Lee (Inje University Ilsan Paik Hosital, Ilsan, Korea); Seung-Ho Hur (Keimyung University Dongsan Hospital, Daegu, Korea); Won-Ho Kim (Daejeon Eulji University Hospital, Dae-jeon, Korea); Nam-Ho Lee (Hallym University Kangnam Sacred Heart Hospital, Seoul, Korea); Ae-Young Her and Yong Hoon Kim (Kangwon National University Hospital, Chuncheon, Korea); Young-Joon Hong (Chunnam National University Hospital, Kwangju, Korea); Hyun-Hee Choi (Hallym University Chunchun Scared Hospital, Chuncheon, Korea); Jong-Pil Park (Jeonnju Presbyterian Medical Center, Jeonju, Korea); and Sang-Wook Lim (CHA University Medical Center, Seongnam, Korea).

REFERENCES

1. Stone GW, Ellis SG, Cannon L, et al; TAXUS V Investigators. Comparison of a polymer-based paclitaxel-eluting stent with a bare metal stent in patients with complex coronary artery disease: a randomized controlled trial. *JAMA*. 2005;294(10):1215-1223.

2. Stone GW, Ellis SG, Cox DA, et al; TAXUS-IV Investigators. A polymer-based, paclitaxel-eluting stent in patients with coronary artery disease. *N Engl J Med*. 2004;350(3):221-231.

3. Kastrati A, Dibra A, Mehilli J, et al. Predictive factors of restenosis after coronary implantation of sirolimus- or paclitaxel-eluting stents. *Circulation*. 2006;113(19):2293-2300.

4. Daemen J, Wenaweser P, Tsuchida K, et al. Early and late coronary stent thrombosis of sirolimus-eluting and paclitaxel-eluting stents in routine clinical practice: data from a large two-institutional cohort study. *Lancet*. 2007;369(9562):667-678.

5. Hong MK, Mintz GS, Lee CW, et al. Intravascular ultrasound predictors of angiographic restenosis after sirolimus-eluting stent implantation. *Eur Heart J*. 2006;27(11):1305-1310.

6. Fujii K, Mintz GS, Kobayashi Y, et al. Contribution of stent underexpansion to recurrence after sirolimus-eluting stent implantation for in-stent restenosis. *Circulation*. 2004;109(9):1085-1088.

7. Fujii K, Carlier SG, Mintz GS, et al. Stent underexpansion and residual reference segment stenosis are related to stent thrombosis after sirolimus-eluting stent implantation: an intravascular ultrasound study. *J Am Coll Cardiol*. 2005;45(7):995-998.

8. Zhang Y, Farooq V, Garcia-Garcia HM, et al. Comparison of intravascular ultrasound versus angiography-guided drug-eluting stent implantation: a meta-analysis of one randomised trial and ten observational studies involving 19,619 patients. *EuroIntervention*. 2012;8(7):855-865.

9. Klersy C, Ferlini M, Raisaro A, et al. Use of IVUS guided coronary stenting with drug eluting stent: a systematic review and meta-analysis of randomized controlled clinical trials and high quality observational studies. *Int J Cardiol*. 2013;170(1):54-63.

10. Jang JS, Song YJ, Kang W, et al. Intravascular ultrasound-guided implantation of drug-eluting stents to improve outcome: a meta-analysis. *JACC Cardiovasc Interv*. 2014;7(3):233-243.

11. Ahn JM, Kang SJ, Yoon SH, et al. Meta-analysis of outcomes after intravascular ultrasound-guided versus angiography-guided drug-eluting stent implantation in 26,503 patients enrolled in three randomized trials and 14 observational studies. *Am J Cardiol*. 2014;113(8):1338-1347.

12. Jakabcin J, Spacek R, Bystron M, et al. Long-term health outcome and mortality evaluation after invasive coronary treatment using drug eluting stents with or without the IVUS guidance: randomized control trial: HOME DES IVUS. *Catheter Cardiovasc Interv*. 2010;75(4):578-583.

13. Chieffo A, Latib A, Caussin C, et al. A prospective, randomized trial of intravascular-ultrasound guided compared to angiography guided stent implantation in complex coronary lesions: the AVIO trial. *Am Heart J*. 2013;165(1):65-72.

14. Kim JS, Kang TS, Mintz GS, et al. Randomized comparison of clinical outcomes between intravascular ultrasound and angiography-guided drug-eluting stent implantation for long coronary artery stenoses. *JACC Cardiovasc Interv*. 2013;6(4):369-376.

15. Windecker S, Kolh P, Alfonso F, et al; Authors/Task Force members. 2014 ESC/EACTS Guidelines on myocardial revascularization: the Task Force on Myocardial Revascularization of the European Society of Cardiology (ESC) and the European Association for Cardio-Thoracic Surgery (EACTS) developed with the special contribution of the European Association of Percutaneous

Copyright 2015 American Medical Association. All rights reserved.

Downloaded From: by a MEDICAL LIBRARY YONSEI UNIVERSITY User on 06/15/2018

Cardiovascular Interventions (EAPCI). *Eur Heart J*. 2014;35(37):2541-2619.

16. Cho S, Shin DH, Kim JS, et al. Rationale and design: impact of intravascular ultrasound guidance on long-term clinical outcomes of everolimus-eluting stents in long coronary lesions. *Contemp Clin Trials*. 2015;40:90-94.

17. Cutlip DE, Windecker S, Mehran R, et al; Academic Research Consortium. Clinical end points in coronary stent trials: a case for standardized definitions. *Circulation*. 2007;115(17):2344-2351.

18. Thygesen K, Alpert JS, Jaffe AS, et al; Joint ESC/ACCF/AHA/WHF Task Force for the Universal Definition of Myocardial Infarction. Third universal definition of myocardial infarction. *Circulation*. 2012;126(16):2020-2035.

19. Moussa ID, Klein LW, Shah B, et al. Consideration of a new definition of clinically relevant myocardial infarction after coronary revascularization: an expert consensus document from the Society for Cardiovascular Angiography and Interventions (SCAI). *J Am Coll Cardiol*. 2013;62(17):1563-1570.

20. Mauri L, Hsieh WH, Massaro JM, Ho KK, D'Agostino R, Cutlip DE. Stent thrombosis in randomized clinical trials of drug-eluting stents. *N Engl J Med*. 2007;356(10):1020-1029.

21. Mintz GS, Nissen SE, Anderson WD, et al. American College of Cardiology clinical expert consensus document on standards for acquisition, measurement and reporting of intravascular ultrasound studies (IVUS): a report of the American College of Cardiology task force on clinical expert consensus documents. *J Am Coll Cardiol*. 2001;37(5):1478-1492.

22. Turco MA, Ormiston JA, Popma JJ, et al. Reduced risk of restenosis in small vessels and reduced risk of myocardial infarction in long lesions with the new thin-strut TAXUS Liberté stent: 1-year results from the TAXUS ATLAS program. *JACC Cardiovasc Interv*. 2008;1(6):699-709.

23. Kim YH, Park SW, Lee SW, et al; Long-DES-II Study Investigators. Sirolimus-eluting stent versus paclitaxel-eluting stent for patients with long coronary artery disease. *Circulation*. 2006;114(20):2148-2153.

24. Park DW, Kim YH, Song HG, et al. Comparison of everolimus- and sirolimus-eluting stents in patients with long coronary artery lesions: a randomized LONG-DES-III (Percutaneous Treatment of LONG Native Coronary Lesions With Drug-Eluting Stent-III) Trial. *JACC Cardiovasc Interv*. 2011;4(10):1096-1103.

25. Claessen BE, Smits PC, Kereiakes DJ, et al. Impact of lesion length and vessel size on clinical outcomes after percutaneous coronary intervention with everolimus- versus paclitaxel-eluting stents pooled analysis from the SPIRIT (Clinical Evaluation of the XIENCE V Everolimus Eluting Coronary Stent System) and COMPARE (second-generation everolimus-eluting and paclitaxel-eluting stents in real-life practice) randomized trials. *JACC Cardiovasc Interv*. 2011;4(11):1209-1215.

26. Naidu SS, Krucoff MW, Rutledge DR, et al. Contemporary incidence and predictors of stent thrombosis and other major adverse cardiac events in the year after XIENCE V implantation: results from the 8,061-patient XIENCE V United States study. *JACC Cardiovasc Interv*. 2012;5(6):626-635.

27. Oemrawsingh PV, Mintz GS, Schalij MJ, Zwinderman AH, Jukema JW, van der Wall EE; TULIP Study. Thrombocyte activity evaluation and effects of Ultrasound guidance in Long Intracoronary stent Placement. Intravascular ultrasound guidance improves angiographic and clinical outcome of stent implantation for long coronary artery stenoses: final results of a randomized comparison with angiographic guidance (TULIP Study). *Circulation*. 2003;107(1):62-67.

28. Hong SJ, Kim MH, Ahn TH, et al. Multiple predictors of coronary restenosis after drug-eluting stent implantation in patients with diabetes. *Heart*. 2006;92(8):1119-1124.

29. Tian NL, Gami SK, Ye F, et al. Angiographic and clinical comparisons of intravascular ultrasound-versus angiography-guided drug-eluting stent implantation for patients with chronic total occlusion lesions: two-year results from a randomised AIR-CTO study. *EuroIntervention*. 2015;10(12):1409-1417.

30. Witzenbichler B, Maehara A, Weisz G, et al. Relationship between intravascular ultrasound guidance and clinical outcomes after drug-eluting stents: the Assessment of Dual Antiplatelet Therapy with Drug-Eluting Stents (ADAPT-DES) study. *Circulation*. 2014;129(4):463-470.

31. Kim JS, Hong MK, Ko YG, et al. Impact of intravascular ultrasound guidance on long-term clinical outcomes in patients treated with drug-eluting stent for bifurcation lesions: data from a Korean multicenter bifurcation registry. *Am Heart J*. 2011;161(1):180-187.

32. Park SJ, Kim YH, Park DW, et al; MAIN-COMPARE Investigators. Impact of intravascular ultrasound guidance on long-term mortality in stenting for unprotected left main coronary artery stenosis. *Circ Cardiovasc Interv*. 2009;2(3):167-177.

33. Kim JS, Kim JH, Shin DH, et al. Serial randomized comparison of strut coverage of everolimus- and first-generation sirolimus-eluting stents. *Can J Cardiol*. 2015;31(6):723-730.

34. Palmerini T, Biondi-Zoccai G, Della Riva D, et al. Stent thrombosis with drug-eluting and bare-metal stents: evidence from a comprehensive network meta-analysis. *Lancet*. 2012;379(9824):1393-1402.

Copyright 2015 American Medical Association. All rights reserved.

Downloaded From: by a MEDICAL LIBRARY YONSEI UNIVERSITY User on 06/15/2018
